# Identification of *N*-Acyl Hydrazones as New Non-Zinc-Binding MMP-13 Inhibitors by Structure-Based Virtual Screening Studies and Chemical Optimization

**DOI:** 10.3390/ijms241311098

**Published:** 2023-07-04

**Authors:** Doretta Cuffaro, Aleix Gimeno, Bianca Laura Bernardoni, Riccardo Di Leo, Gerard Pujadas, Santiago Garcia-Vallvé, Susanna Nencetti, Armando Rossello, Elisa Nuti

**Affiliations:** 1 Department of Pharmacy, University of Pisa, Via Bonanno 6, 56126 Pisa, Italy; doretta.cuffaro@unipi.it (D.C.); bianca.bernardoni@phd.unipi.it (B.L.B.); riccardo.dileo@phd.unipi.it (R.D.L.); susanna.nencetti@unipi.it (S.N.); armando.rossello@unipi.it (A.R.); 2 Research Group in Cheminformatics & Nutrition, Departament de Bioquímica i Biotecnologia, Universitat Rovira i Virgili, Campus de Sescelades, 43007 Tarragona, Spain; aleix.givi2@gmail.com (A.G.); gerard.pujadas@gmail.com (G.P.); santi.garcia-vallve@urv.cat (S.G.-V.)

**Keywords:** virtual screening, MMP-13 inhibitors, osteoarthritis, *N*-acyl hydrazones, protein-ligand docking

## Abstract

Matrix metalloproteinase 13 plays a central role in osteoarthritis (OA), as its overexpression induces an excessive breakdown of collagen that results in an imbalance between collagen synthesis and degradation in the joint, leading to progressive articular cartilage degradation. Therefore, MMP-13 has been proposed as a key therapeutic target for OA. Here we have developed a virtual screening workflow aimed at identifying selective non-zinc-binding MMP-13 inhibitors by targeting the deep S1′ pocket of MMP-13. Three ligands were found to inhibit MMP-13 in the µM range, and one of these showed selectivity over other MMPs. A structure-based analysis guided the chemical optimization of the hit compound, leading to the obtaining of a new *N*-acyl hydrazone-based derivative with improved inhibitory activity and selectivity for the target enzyme.

## 1. Introduction

**Figure 1:**
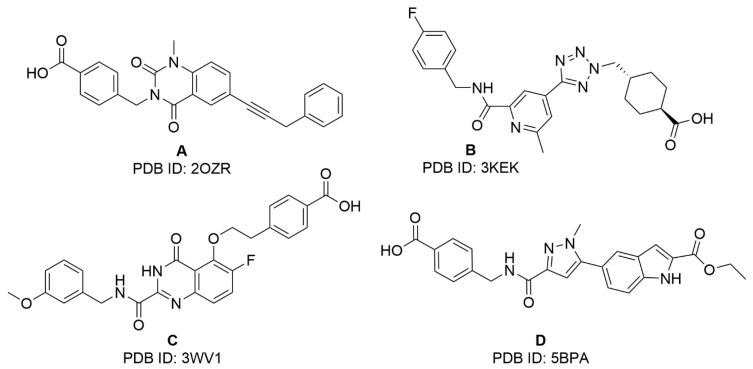
Chemical structures of non-zinc-binding selective MMP-13 inhibitors (**A** [19], **B** [20], **C** [21], and **D** [22]) with reported X-ray crystal structures in complex with the MMP-13 catalytic domain used for the VS set-up.

Osteoarthritis (OA) is the most common form of arthritis [1], affecting half of the elderly population (>65 years) [2]. It is characterized by the progressive degradation of articular collagen and can ultimately result in the prosthetic replacement of joints as they become completely dysfunctional. Matrix metalloproteinase 13 (MMP-13), also known as collagenase-3, plays a central role in the pathology as it is the main enzyme responsible for the cleavage of type II collagen in patients with OA [3]. MMP-13 is significantly overexpressed in the joints and articular cartilage in patients with OA and is not present in normal adult cartilage; therefore, it has been proposed as a key therapeutical target for the treatment of OA [4]. MMP-13 belongs to the MMP family, which consists of 23 zinc-dependent enzymes responsible for the degradation of different extracellular matrix (ECM) components [5]. In addition to tissue remodeling, MMPs are involved in the cleavage of many non-matrix targets, such as cell surface receptors, cytokines, chemokines, cell-cell adhesion molecules, clotting factors, and other proteinases [6]. Despite some broad-spectrum MMP inhibitors (MMPIs) having halted the destruction of cartilage in preclinical assays, they have failed clinical trials as patients developed musculoskeletal syndrome (MSS), possibly resulting from the alteration of the physiological functions of different members of the MMP family [7,8]. Therefore, selectivity is currently considered a priority in the development of MMP inhibitors, even if the high homology among MMP catalytic domains makes it a challenging task. So far, in MMPI design, the catalytic zinc ion chelation has been crucial, leading to inhibitors presenting mainly a hydroxamic acid or carboxylic acid as a zinc-binding group (ZBG) [9,10], with nanomolar activity but poor selectivity. In fact, the presence of a ZBG can negatively affect the selective targeting of a specific MMP, cross-interfering with other metalloproteases such as ADAMs (A Disintegrin and Metalloproteinase) [11,12] and ADAMTSs (A Disintegrin and Metalloproteinase with Thrombospondin Motifs) [13], but also with other zinc metalloenzymes such as carbonic anhydrases [14,15] or histone deacetylases [16,17]. In this regard, the characteristics of the catalytic binding site of MMP-13 are slightly different from those of other MMPs, thus providing an edge in the identification of selective inhibitors for this enzyme. More specifically, an adjacent region to the catalytic site, known as the S1′ pocket, is different in MMP-13 as the loop that delimits the pocket (Ω-loop) is longer and shows more flexibility in MMP-13 than in other MMPs [18]. In particular, the Ω-loop of MMP-13 encloses the so-called S1″ specificity pocket. This allows for an opportunity to identify inhibitors with a different binding mode that is not possible for other enzymes in the MMP family. Several non-zinc chelating inhibitors (Figure 1) exploited this difference in the MMP-13 binding site to achieve selectivity for this enzyme, as shown by their X-ray crystal structures [19,20,21,22]. In the present paper, we used these crystallographic data to design a virtual screening (VS) methodology able to identify MMP-13 inhibitors that can adopt a similar binding mode and therefore achieve selectivity towards MMP-13. Enzymatic assays of a limited selection of candidate compounds (20) allowed us to find three novel hits that were structurally unrelated to the known MMPIs. The most promising compound underwent a hit optimization study in order to improve its activity and selectivity profile, and a novel series of 12 derivatives was synthesized and tested in vitro.

## 2. Results and Discussion

### 2.1. Virtual Screening Studies

**Figure 2:**
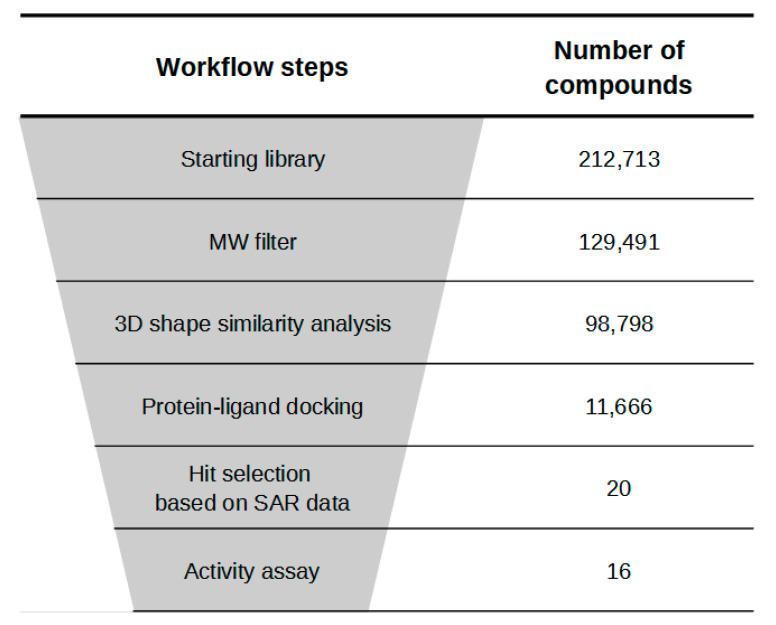
Virtual Screening workflow.

Co-crystallized MMP-13 inhibitors that bind to the S1′ pocket without interacting with the catalytic zinc ion show a similar binding mode [19,20,21,22]. All of them present two common characteristics: (a) they contain two aromatic rings or ring systems at both ends of the molecule (with the exception of the co-crystallized inhibitor in the structure with PDB code: 3KEK [20], which instead presents a cyclohexyl group at one of its ends); and (b) they adopt a characteristic *U* shape, establishing hydrogen bond interactions between the core of the molecule and Thr245, Thr247, or both residues. We used these characteristics to design a VS workflow capable of identifying compounds that bind in a similar manner to the S1′ pocket of MMP-13. The VS workflow, which was applied to an initial library of 212,713 compounds obtained from Specs [23], consisted of four steps that are described in this section (Figure 2).

First, the compounds were filtered by molecular weight (MW); then, a shape-based alignment was performed to keep only the compounds that could adopt a conformation similar to that of the co-crystallized ligands; next, protein-ligand docking was performed on MMP-13; and, finally, compounds were selected based on the interactions with the binding site of MMP-13 that improved MMP-13 inhibitor activity in previously reported structure-activity relationship (SAR) studies. The compounds obtained from the Specs database were filtered by MW in order to discard compounds too small to fulfill the posterior protein-ligand docking constraints and compounds too large compared to the reference ligands used for the subsequent shape-based alignment, therefore reducing the computational time of the subsequent steps. The 300–700 Da range was selected as the filter, taking into account that the compounds used as references in the shape-based alignment step have MWs between 392 and 491 Da. As a result of this first step, 83,222 compounds were filtered out (Figure 2). Next, a maximum of 10 conformations were generated for each compound that survived the MW filter. These conformations were compared to those of selective MMP-13 inhibitors co-crystallized with MMP-13 that bind to the S1′ pocket and do not contain a ZBG. Only conformations similar to those of co-crystallized ligands were kept in order to reduce the computational cost of the VS, as compounds unable to adopt a similar shape to that of the co-crystallized ligands would likely not fit in the S1′ cavity during the protein-ligand docking step. As a result of this step, 30,693 compounds were filtered out (Figure 2). The ligands resulting from the shape similarity filter were docked onto MMP-13 using the crystal structure with PDB code 3WV1 [21], as it contains an inhibitor that binds to the S1′ pocket. In order to discard ligands unable to adopt a similar binding mode in the S1′ pocket to that of previously known selective MMP-13 inhibitors, two positional constraints were defined (one closer to the zinc-binding region and another one deep in the S1′ pocket) to be fulfilled by aromatic atoms. Moreover, it was required that the ligand perform a hydrogen bond interaction with Thr245 or Thr247, as all the co-crystallized inhibitors performed at least one of these interactions with the core of the molecule. In order to select the compounds that performed the appropriate interactions in the S1′ pocket of MMP-13, several SAR studies were analyzed to obtain information regarding which interactions are important to achieve high activity towards MMP-13. In particular, a potent MMP-13 inhibitor should: (1) have a negatively charged ring substituent that can establish a salt bridge interaction with Lys140, which is unique at the bottom of the S1″ side pocket of MMP-13 [21,22,24]; (2) make a π-π interaction with Tyr246 and Phe252 [21]; (3) have a hydrophobic moiety occupying the S1″ pocket [21,22]; (4) have an appropriate linker to join the S1′ pocket with the S1″ side pocket [24]; (5) have a hydrogen or halogen bond acceptor towards Met253 main chain nitrogen [21]; (6) make a hydrophobic interaction with Pro255 [20,24]; (7) have a hydrogen bond acceptor towards the side chain of Thr247 [21,22]; (8) establish hydrophobic contacts in the S1′ pocket [24]; (9) have a hydrogen bond acceptor towards Thr245 main chain nitrogen [24]; (10) have a hydrogen bond donor towards Ala238 main chain carbonyl oxygen [24]; and (11) have an appropriate ring substituent in the region close to the zinc-binding group [20,22].

After docking, the top 10 docked poses for each compound were selected based on their docking scores. The interactions they performed with the protein were carefully inspected in order to select the compounds that accomplished the criteria obtained through the analysis of the above-mentioned SAR data. Ideally, in this step, we would like to obtain a compound that meets the 11 criteria. However, this was not the case, as the compound that accomplished more criteria was compound **1** (Figure S1), with a total of eight. Therefore, this compound and compounds that fulfilled most of the criteria were selected, obtaining 20 compounds for in vitro tests (Figure S1). The structures of these 20 compounds were compared, using their molecular fingerprints, to those of previously reported MMP-13 inhibitors in the Reaxys [25] database. Except for compound **14**, which showed a Tanimoto value of 0.71 with a described MMP-13 inhibitor, the Tanimoto similarity values of the selected compounds with any of the previously reported MMP-13 inhibitors analyzed were at most 0.6. Upon visual inspection, compound **14** was not considered structurally similar to the MMP-13 inhibitor obtained from Reaxys (see Figure S2). Moreover, hit compounds that were structurally similar to previously selected hit compounds were discarded to ensure that the final 20 compounds selected for in vitro tests were structurally different from each other (see Figure S3). Therefore, the hit compounds obtained by this virtual screening methodology proved not only to be different from previously reported MMP-13 inhibitors but also structurally diverse.

After the selection of the 20 hit compounds, they were purchased from Specs, and their activity for MMP-13 was analyzed in vitro. The activity data (% inhibition at 100 μM) were determined by a fluorometric assay on human recombinant enzyme, and four compounds were discarded due to very low solubility in DMSO or high fluorescence emission interfering with the assay. ARP100 [26], a hydroxamate-based MMP inhibitor previously developed by our research group, was used in the same assay conditions as the reference compound. To exclude any possible nonspecific inhibition of MMP-13 due to aggregate formation, we performed all the assays in the presence of 0.05% Brij-35, a nonionic detergent similar to Triton X-100, as indicated by Shoichet et al. [27]. Three ligands, out of the twenty tested, presented a % inhibition >40 at 100 μM and were further characterized. All other compounds were not further investigated (see Figure S1 for chemical structures). In Table 1, are displayed the structures, Specs codes, clogP, and MMP-13% inhibition of the selected ligands.

### 2.2. Enzymatic Assays

After the preliminary screening, compounds **11**, **12**, and **13** (Table 1) displayed the highest MMP-13 inhibitory activity, and their IC_50_ values were calculated (91 μM, 105 μM, and 14.6 μM, respectively). Next, the inhibitory activities of these three compounds towards MMP-1, MMP-2, MMP-9, and MMP-14 were determined (Table 2). The selectivity profile of the three ligands pointed out that compound **13**, with an *N*-acyl hydrazone scaffold, presented low micromolar activity against MMP-13 and a promising selectivity over the other tested MMPs. For this reason, **13** was chosen as the hit compound and underwent an optimization study in order to improve its activity and selectivity profile. The *N*-acyl hydrazone group has been recently introduced in several approved drugs and molecules in clinical trials since it represents a peptide-mimetic subunit endowed with high metabolic stability [29,30].

### 2.3. Structure-Based Hit Optimization

**Figure 3:**
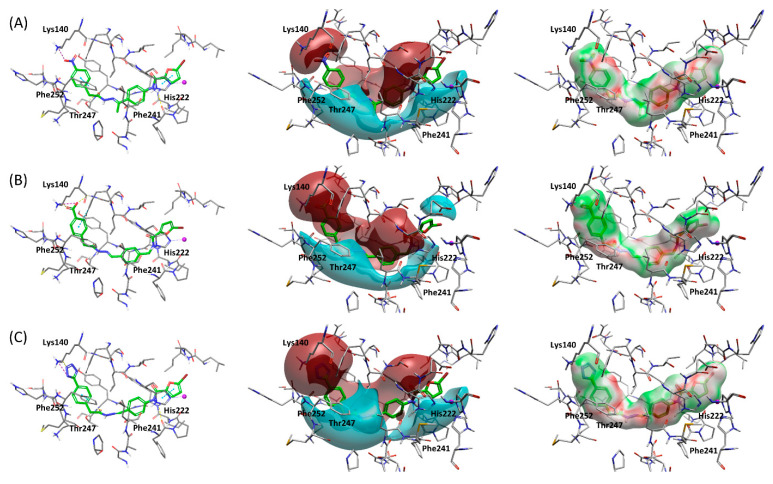
Docked poses of compounds **13**, **13m**, and **13n** (panels (**A**), (**B**), and (**C**), respectively) in the binding site of MMP-13. The first picture in each panel shows the intermolecular interactions predicted by docking (hydrogen bonds are colored in yellow, π stacking interactions are colored in cyan, and salt bridges are colored in magenta). The second picture shows the positive and negative electrostatic field potentials of the compound (colored blue and red, respectively). The third picture shows the molecular surface of the compound colored by the electrostatic complementarity (EC) to the receptor (green: good EC and red: bad EC).

The docked position of compound **13** (Figure 3A and Figure S4A) in the MMP-13 binding site shows the characteristic U shape observed in MMP-13 inhibitors that bind to the S1′ pocket. The compound contains two amide bonds, one on each side, that establish hydrogen bond interactions with Phe241 and Thr247. These hydrogen bond interactions and the planar character of amide bonds were deemed important for the binding mode of the compound, so the amide moieties were not altered during the optimization. At the S1′ pocket, compound **13** establishes a π stacking interaction with Phe252 and a salt bridge interaction with Lys140. Given the strong nature of the electrostatic interaction with Lys140 and the fact that this residue is not present in other MMPs, this region of the molecule should be highly important for both activity and selectivity, and it was further explored in the optimization. At the zinc-binding region, compound **13** establishes a π stacking interaction with His222, but the bromine substituent may cause low solubility. Thus, to increase compound solubility, modifications were proposed in this region as well as to the methyl group present at the center of the molecule.

On the basis of these considerations, we planned to synthesize new derivatives of **13** in order to explore: (i) the *p*-substitution on the benzyl ring (R); (ii) the replacement of the methyl on the *N*-acyl hydrazone moiety with an hydrogen atom (R_1_); (iii) the elongation of the linker between the central phenyl ring and amido group; and (iv) the substitution on the furan nucleus (R_2_) (compounds **13a**–**o**, Table 3). The rigid parts of the molecule have been kept to maintain the degrees of freedom and the conformation of the compounds.

### 2.4. Chemistry

**Scheme 1:**
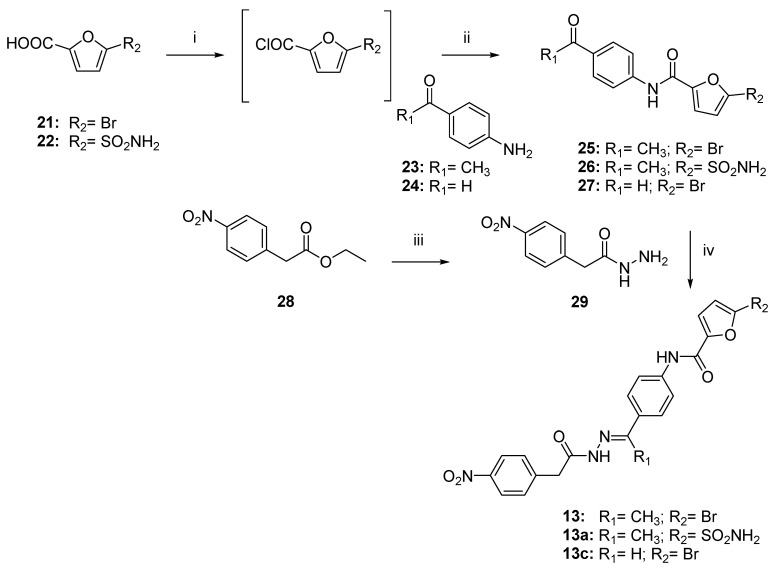
Reagents and conditions: (i) SOCl_2_ or (COCl)_2_, dioxane, 100 °C, 12 h; (ii) pyridine, dioxane, 1 h (**25**: 73%; **26**: 18%; **27**: 73% over two steps); (iii) NH_2_NH_2_, EtOH, 4 h, 82%; and (iv) EtOH, reflux (**13**: 18 h, 86%; **13a**: 5 h, 45%; **13c**: 5 h, 98%).

**Scheme 2:**
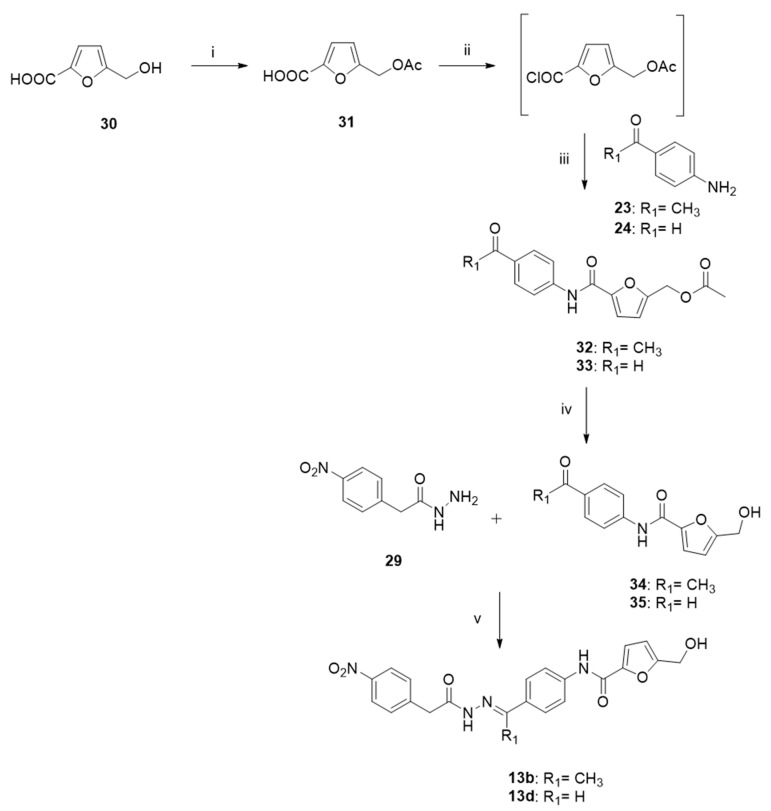
Reagents and conditions: (i) Ac_2_O, Et_2_O, Et_3_N, 3.5 h, 85%; (ii) SOCl_2_, dioxane, 100 °C, 12 h; (iii) pyridine, dioxane, 1 h, rt (**32**: 55%; **33**: 69% over two steps); (iv) NH_3_/MeOH 7N, 1.5 h (**34**: quantitative; **35**: 61%); and (v) EtOH, reflux, 18 h (1**3b**: 77%; **13d**: 95%).

**Scheme 3:**
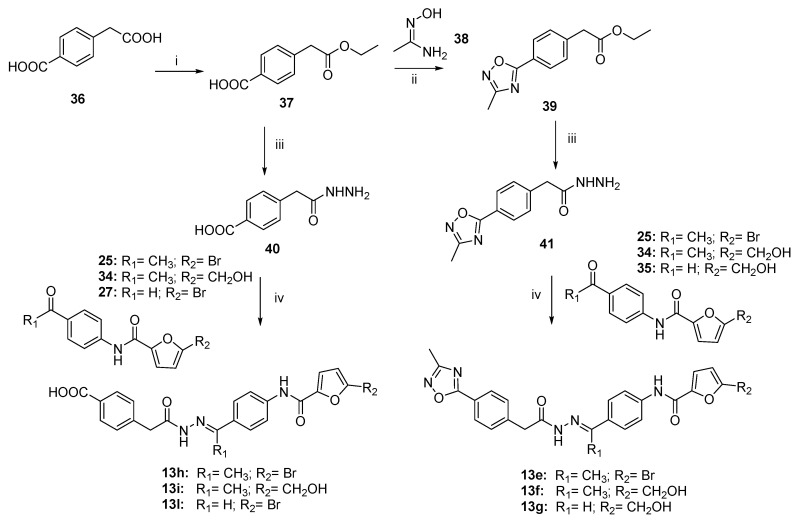
Reagents and conditions: (i) SOCl_2_, EtOH, toluene, 100 °C, o/n, 87%; (ii) 1. SOCl_2_, DCM dry, 4 h; 2. K_2_CO_3_, acetone, o/n; 3. 130 °C, solvent free, 3 h; 36% over three steps; (iii) NH_2_NH_2_, EtOH, reflux (**40**: 6 h, 64%; **41**: 3 h, 89%); and (iv) EtOH, reflux (3–12 h; 43–85%).

**Scheme 4:**
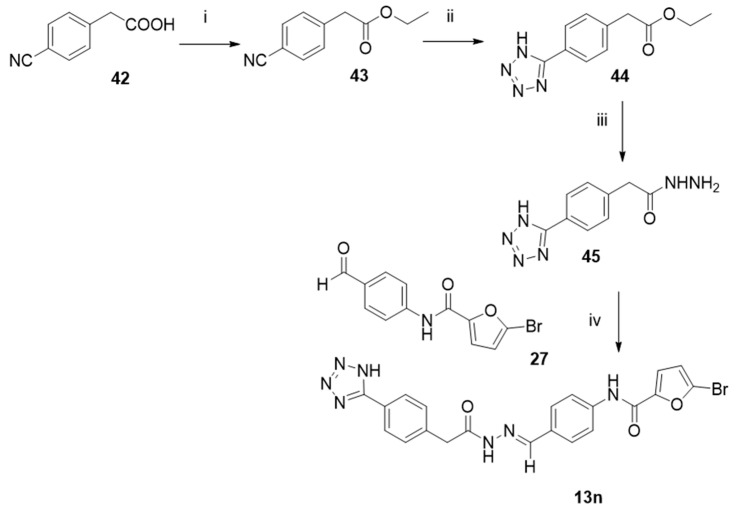
Reagents and conditions: (i) SOCl_2_, EtOH reflux, 2 h, 97%; (ii) Me_3_SnN_3_, THF, 3 days, 69%; (iii) NH_2_NH_2_, EtOH reflux, 4 h, 30%; and (iv) EtOH reflux, 18 h, 73%.

**Scheme 5:**
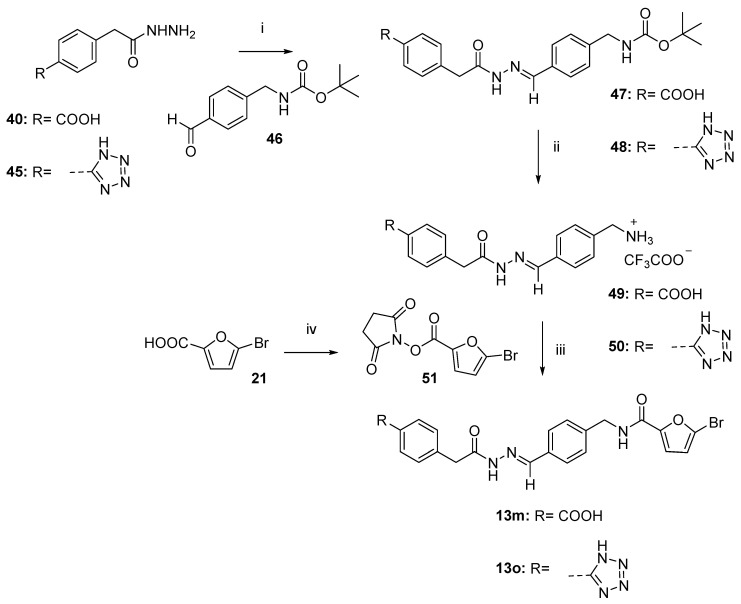
Reagents and conditions: (i) EtOH, reflux, 18 h (**47**: 70%; **48**: 44%); (ii) TFA, DCM dry, 2 h, quantitative; (iii) DIPEA, DMF dry, (**13m**: 40%; **13o**: 41%); and (iv) *N*-hydroxysuccinimide, EDC, DCM dry, 18 h, 71%.

New compounds **13a**–**o** were obtained as described in Scheme 1, Scheme 2, Scheme 3, Scheme 4 and Scheme 5. Hit compound **13** was synthesized to be used as a positive control in the enzymatic assays.

The synthesis of *N*-acyl hydrazones **13**, **13a,** and **13c** is outlined in Scheme 1. The condensation of carboxylic acids **21** and **22** with aromatic amines **23** or **24** was carried out through the intermediate formation of acyl chlorides by treatment with SOCl_2_ or (COCl)_2_, to give the corresponding amides **25**–**27**. Hydrazide **29** was obtained by direct treatment of ethyl ester **28** with hydrazine hydrate in equimolar proportion in EtOH. Finally, the target compounds **13**, **13a**, and **13c** were obtained by conjugation of hydrazide **29** with the proper ketones **25** and **26** or aldehyde **27** in EtOH at reflux. The final compounds were easily isolated by crystallization in the reaction solvent. 

The synthesis of *N*-acyl hydrazones **13b** and **13d** is reported in Scheme 2. The commercially available furoic alcohol **30** was acetylated by treatment with Ac_2_O/Et_3_N to yield the furoic acetate **31**. Analogously to the synthesis described in Scheme 1, the furoic carboxylic acid **31** protected on the alcoholic function was condensed with aromatic amines **23** or **24** by treatment with thionyl chloride, affording amides **32** and **33**. The latter were deprotected by treatment with a methanolic ammonia solution to give the corresponding alcohols. The final compounds **13b** and **13d**, obtained by conjugation of hydrazide **29** with the proper ketone **34** or aldehyde **35** in EtOH, were isolated as pure crystals from the reaction solvent. 

The synthesis of *N*-acyl hydrazones **13e**–**l** is reported in Scheme 3. Dicarboxylic acid **36** was selectively protected as an ethyl ester by controlled treatment with SOCl_2_ in EtOH, affording the monoester **37**. 5-Methyl-1,2,4 oxadiazole derivative **39** was obtained by cyclodehydration reaction from the known amidoxime **38** [31] and ethyl ester **37**. The resulting ethyl esters **37** and **39** were directly transformed into the corresponding hydrazides **40** and **41** by hydrazinolysis. Lastly, the condensation between the hydrazides **40** or **41** with the proper ketone (**25** or **34**) or aldehyde (**27** or **35**) in EtOH afforded the *N*-acyl hydrazones **13h**–**l** and **13e**–**g** purified by crystallization.

The *N*-acyl hydrazone **13n** was prepared following the synthetic path described in Scheme 4. Ethyl ester **43** was obtained from carboxylic acid **42** by esterification with SOCl_2_. Subsequently, 1*H*-tetrazole derivative **44** was obtained by catalytic cyclization promoted by trimethylstannyl azide from aryl nitrile **43.** The target compound **13n** was afforded in high yield by condensation between hydrazide **45** and aldehyde **27**. 

The synthesis of the longest derivatives with n = 1, *N*-acyl hydrazones **13m** and **13o**, is shown in Scheme 5. The *N*-acyl hydrazones **47** and **48** were prepared by condensation between the commercially available aldehyde **46** and the proper hydrazides **40** or **45**. The removal of Boc protection was conducted by acid hydrolysis using an excess of trifluoroacetic acid (TFA) to give the pure trifluoroacetate salts of benzylamines **49** and **50**. The furoic carboxylic acid **21** was activated as an NHS-ester by reaction with *N*-hydroxysuccinimide and EDC as coupling agents. The resulting NHS-ester **51** was coupled with amines **49** or **50** to give the corresponding compounds **13m** or **13o** in high yields.

**Figure 4:**
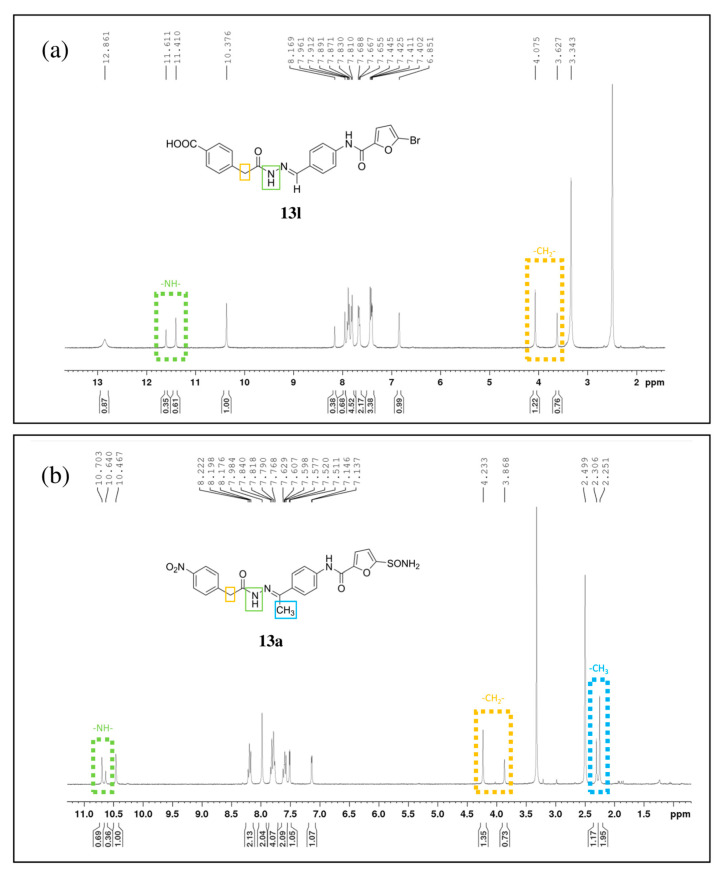
Significative ^1^H NMR spectra (400 MHz, DMSO-*d*_6_) of representative *N*-acyl hydrazones **13l** (**a**) and **13a** (**b**).

Usually, the *N*-acyl hydrazone structure (-C(O)-N-N=C<) is a hybrid between amide and imine functional groups, exhibiting both geometric and conformational stereoisomerism [32]. Rotation along C=N linkage results in the *E*/*Z* stereoisomers, where the *E* conformation is reported to be usually the preferred geometry in solution due to the unfavorable steric restriction of the *Z* form [33,34]. Moreover, the rotation across the amide C(O)-NH bond gives geometrical isomerism as synperiplanar (sp) and antiperiplanar (ap) conformers [35]. For these reasons, all the *N*-acyl hydrazones here reported (**13** and **13a**–**o**) are described in their *E* form and as a mixture of synperiplanar (sp) and antiperiplanar (ap) conformers. As already reported by Munir et al. [35], in all the ^1^H NMR spectra of the synthesized *N*-acyl hydrazones (**13** and **13a**–**o**; see Supporting Information and Experimental section), a particular pattern of two signal sets was detected, corresponding to the mixture of rotational syn- and antiperiplanar conformers. In fact, in the ^1^H NMR spectra resolved in DMSO-*d*_6_, duplicated signals were observed for -CO*NH*N-, -C*H*_2_-, and -N=C(C*H*_3_) protons. In Figure 4, the ^1^H NMR spectra of two representative *N*-acyl hydrazones, **13l** and **13a**, are reported. In both spectra, the chemical shift of the -CO*NH*N- proton (in green) resonated around 10.5–12 ppm, resulting in a double singlet, indicating the syn and the ap forms. Similar behavior is shown by benzylic -C*H_2_*- (in yellow) and methyl imine (-CH_3_) (in blue) protons, which resonate, respectively, at 4.2–3.8 ppm and 2.3–2.25 ppm. For -C(O)N*H*N- doublet-like peaks, the downfield signal was attributed to the antiperiplanar (ap) conformer, whereas the upfield signals were attributed to the synperiplanar (sp) conformer, as established from the literature [36,37,38]. On the contrary, regarding the methylene (-C*H*_2_) proton, the upfield signal is the anti-form and the downfield signal is the syn-isomer, as evidently demonstrated by the integration of the peaks. The ratio between two conformers was calculated considering the integral intensities of the paired peaks, revealing that a DMSO-*d*_6_ solution of these compounds contained *E*-synperiplanar and *E*-antiperiplanar conformers in an approximate 2:1 ratio with slight variation from compound to compound. 

### 2.5. SAR Analysis

All new derivatives **13a**–**o** were tested by a fluorometric assay on recombinant MMP-13 in comparison with hit compound **13**, and their inhibitory activity is reported in Table 3. The replacement of the bromine substituent on the furan ring (R_2_) with a sulfonamido or an alcohol group caused a drop in activity, with derivatives **13a** and **b** displaying an IC_50_ > 100 µM.

In general, all derivatives bearing a hydrogen atom in R_1_ showed improved activity with respect to their *N*-methyl analogues, as can be seen by comparing compound **13d** with compound **13b** and compound **13g** with compound **13f**. 

The introduction of a 1,2,4-oxadiazole ring in R (as in compounds **13e** and **f**) caused a decrease in inhibitory activity for MMP-13 relative to **13**. This heterocycle, known as an ester isostere, is present in many biologically active compounds [39] and was chosen in an attempt to ameliorate the solubility of our original *N*-acyl hydrazone scaffold. Actually, the simultaneous introduction of the oxadiazole ring in R with an alcohol group in R_2_ and a hydrogen in R_1_ caused an improvement in solubility, as can be seen from the logP value calculated for compound **13g** with respect to **13** (Table 4), but did not increase the inhibitory activity. 

On the contrary, the replacement of the nitro group in R with an acidic moiety, such as a carboxylic acid in **13l** or a 1*H*-tetrazole in **13n**, led to improved inhibitory activity relative to **13c**. The incorporation of these acidic moieties introduced more negative electrostatic surfaces close to the residue Lys140, which resulted in better electrostatic complementarities between these compounds and the enzyme at the S1″ pocket, thus increasing their activities (Figure 3C and Figure S4C).

On the basis of these results and in order to increase its inhibitory potency, we decided to further modify **13l** by introducing a methylene spacer between the central phenyl ring and amido group (n = 1). In fact, this modification could allow our hit compound to better interact with Lys140 in the S1″ pocket by establishing the salt bridge necessary for a proper fit with the MMP-13 binding site (Figure 3B and Figure S4B). As expected, compound **13m** resulted in the best of the series, showing an 8-fold increase in inhibitory potency against MMP-13 (IC_50_ = 1.8 µM) relative to **13**.

Of note, the same modification in the tetrazole analogue **13o** caused a drop in activity, probably due to the excessive bulkiness of this substituent, negatively affecting the binding with the enzyme. 1*H*-tetrazole is an aromatic heterocyclic bioisoster of the carboxylic acid group, characterized by a higher lipophilicity and a similar acidity but a different volume [40].

In general, derivatives **13a**–**o** show good predicted ADMET properties (see Table 4). Thus, all of them fulfill the Lipinski rule of five: they have good intestinal absorption and show week/moderate cardiotoxicity. Only high hepatotoxicity is predicted for all of them, and this is necessary to be experimentally tested in future studies and before the next rounds of optimization of **13m** (or other) derivatives.

Finally, the selectivity profile of **13m** was evaluated to verify if the optimization process has led to a loss of selectivity for MMP-13. The results reported in Table 5 show that, with the exception of MMP-2, the selectivity over the other tested enzymes has been maintained or improved with respect to our hit compound **13**. In particular, **13m** displayed a 100-fold selectivity for MMP-13 over MMP-1 and MMP-14.

## 3. Materials and Methods

### 3.1. Chemistry

Melting points were determined on a Leica Galen III Microscope (Leica/Cambridge Instruments, Cambridge, UK) and are uncorrected. ^1^H and ^13^C NMR spectra were recorded on a Bruker Avance III HD 400 MHz spectrometer (Fällander, Switzerland). Chemical shifts (δ) are reported in parts per million, and coupling constants (*J*) are reported in hertz (Hz). ^13^C NMR spectra were fully decoupled. The following abbreviations were used to explain multiplicities: singlet (s), doublet (d), triplet (t), double doublet (dd), broad (br), and multiplet (m). Chromatographic separations were performed on silica gel columns by flash column chromatography (Kieselgel 40, 0.040−0.063 mm, Merck, Darmstad, Germany) or using ISOLUTE Flash Si II cartridges (Biotage) or using an Isolera automatic system (Biotage, Uppsala, Sweden) and SFÄR HC Duo silica cartridges (Biotage, Uppsala, Sweden). Reactions were followed by thin-layer chromatography (TLC) on Merck aluminum silica gel (60 F254) sheets that were visualized under a UV lamp. Evaporation was performed in vacuo (rotating evaporator). Sodium sulfate was always used as the drying agent. Commercially available chemicals were purchased from Merck (Darmstad, Germany). Elemental analysis was used to determine the purity of the target compounds (Table S1). Analytical results are within ±0.4% of the theoretical values. High-resolution ESI-MS spectra were recorded by direct injection at 5 (positive) and 7 (negative) μL min^−1^ flow rates in an Orbitrap high-resolution mass spectrometer (Thermo, San Jose, CA, USA), equipped with a HESI source.

5-(acetoxymethyl)furan-2-carboxylic acid (**31**).

Triethylamine (2 mL, 14.07 mmol, 2 eq) was added to a white solution of commercial 5-(hydroxymethyl)furan-2-carboxylic acid (**30**) (1 g, 7.03 mmol, 1 eq) in Et_2_O (20.3 mL). Lastly, Ac_2_O (0.9 mL, 9.14 mmol, 1.3 eq) was added dropwise at 0 °C, and the reaction mixture was stirred at RT for 3.5 h. The crude was dissolved in HCl 1N (125 mL) and extracted (3 × 125 mL) with Et_2_O. The organic phases were combined, dried over Na_2_SO_4_, filtrated, and evaporated. The pure product **31** was obtained as a white solid (1165 mg). Yield: 90%. ^1^H NMR (400 MHz, DMSO-*d*_6_) δ: 13.15 (br s, 1H); 7.17 (d, *J* = 3.2 Hz, 1H); 6.67 (d, *J* = 3.2 Hz, 1H); 5.08 (s, 2H); and 2.06 (s, 3H). 

#### 3.1.1. General Procedure to Synthesize Conjugates **25**–**27** and **32**–**33**

To a solution of furoic acids **21**, **22**, and **31** (1 eq) in dioxane (1 mL/mmol) or a mixture of DCM and a few drops of DMF, SOCl_2_ (4 eq) was added under a nitrogen atmosphere. The reaction mixture was stirred at 100 °C for 12 h and then evaporated under an inert atmosphere (N_2_). The crude product was dissolved in dioxane, or DCM (1.10 mL/mmol), and pyridine (1 eq) and the commercial aryl amines **23**–**24** (1 eq) were added under a nitrogen atmosphere. The reaction was stirred at RT for 1 h and then diluted with EtOAc. The organic phase was washed with water, NaHCO_3_ saturated solution, and HCl 1N. The organic layer was dried over Na_2_SO_4_, filtered, and evaporated under reduced pressure. The pure product (**25**–**27** and **33**) was afforded either without any further purification or by column chromatography (**32**).

N-(4-acetylphenyl)-5-bromofuran-2-carboxamide (**25**).

The title compound was synthesized as previously reported in the general procedure, starting from commercial furoic acid **21** (500 mg, 2.62 mmol) dissolved in dioxane and using commercial 4-aminoacetophenone **23** (354 mg). After workup, compound **25** was obtained as a yellow/orange solid (565 mg) without any further purification. Yield: 73%. ^1^H NMR (400 MHz, DMSO-*d*_6_) δ: 10.51 (s, 1H); 7.97–7.95 (m, 2H); 7.90–7.87 (m, 2H); 7.43 (d, *J* = 3.2 Hz, 1H); 6.86 (d, *J* = 3.2 Hz, 1H); and 2.54 (s, 3H).

N-(4-acetylphenyl)-5-sulfamoylfuran-2-carboxamide (**26**).

The title compound was synthesized as previously reported in the general procedure, starting from the commercial 5-(aminosulfonyl)-2-furoic acid **22** (100 mg, 0.523 mmol) dissolved in DCM/DMF and using 4-aminoacetophenone **23** (65mg). After workup, the crude was purified by flash chromatography using an ISOLUTE Si II 5 g cartridge (isocratic gradient: CHCl_3_/MeOH 30:1 *v*/*v*) to give the desired product **26** as a white solid (27 mg). Yield: 18%. ^1^H NMR (400 MHz, CDCl_3_) δ: 10.62 (s, 1H); 8.00–7.98 (m, 4H); 7.89–7.87 (m, 2H); 7.55 (d, *J* = 4 Hz, 1H); 7.15 (d, *J* = 3.6 Hz, 1H); and 2.55 (s, 3H).

5-bromo-N-(4-formylphenyl) furan-2-carboxamide (**27**).

The title compound was synthesized as previously reported in the general procedure, starting from the commercial furoic acid **21** (500 mg, 2.62 mmol) dissolved in dioxane and using the commercially available 4-aminobenzaldehyde **24** (317 mg). After workup, compound **26** was obtained as a yellow solid (555 mg) without any further purification. Yield: 72.5%. ^1^H NMR (400 MHz, DMSO-*d*_6_) δ: 10.57 (s, 1H); 9.91 (s, 1H); 7.98–7.97 (m, 2H); 7.91–7.89 (m, 2H); 7.45 (d, *J* = 8.8 Hz, 1H); and 6.87 (d, *J* = 8.8 Hz, 1H).

(5-((4-acetylphenyl)carbamoyl)furan-2-yl)methyl acetate (**32**).

The title compound was synthesized as previously reported in the general procedure, starting from the derivative **31** (1.00 g, 5.43 mmol) dissolved in dioxane and 4-aminoacetophenone **23** (733 mg). After workup, compound **32** was purified by flash chromatography using an ISOLUTE Si II 5 g cartridge (*n*-hexane/EtOAc in a gradient from 40:1 to 1:1 *v*/*v*) to give the desired product **32** as a white solid (896 mg). Yield: 55%. ^1^H NMR (400 MHz, CDCl_3_) δ: 8.22 (br s, 1H); 8.00–7.97 (m, 2H); 7.79–7.76 (m, 2H); 7.24 (d, *J* = 3.6 Hz, 1H); 6.586 (d, *J* = 3.6 Hz, 1H); 5.12 (s, 2H); 2.59 (s, 3H); and 2.12 (s, 3H).

(5-((4-formylphenyl)carbamoyl)furan-2-yl)methyl acetate (**33**).

The title compound was synthesized as previously reported in the general procedure, starting from the derivative **31** (720 mg, 3.90 mmol) dissolved in dioxane and 4-aminoacetophenone **24** (474 mg). After workup, compound **33** was obtained as an orange solid (772 mg) without any further purification. Yield: 69%. ^1^H NMR (400 MHz, DMSO-*d*_6_) δ: 10.55 (s, 1H); 9.91 (s, 1H); 8.00–7.98 (m, 2H); 7.92–7.89 (m, 2H); 7.41 (d, *J* = 3.6 Hz, 1H); 6.75 (d, *J* = 3.6 Hz, 1H); 5.13 (s, 2H); and 2.08 (s, 3H).

N-(4-acetylphenyl)-5-(hydroxymethyl)furan-2-carboxamide (**34**).

To a solution of the ketone **32** (250 mg, 0.83 mmol, 1 eq) in MeOH (3 mL), NH_3_-MeOH 7N (6 mL) was added. The resulting mixture was stirred at RT for 1.5 h under a nitrogen atmosphere. Then, the reaction mixture was concentrated under vacuum conditions and purified by trituration with *n*-hexane to give pure compound **34** as a solid (215 mg). Yield: quantitative. ^1^H NMR (400 MHz, CDCl_3_) δ: 8.22 (br s, 1H); 7.99 (d, *J* = 8.8 Hz, 2H); 7.77 (d, *J* = 8.8 Hz, 2H); 7.24 (d, *J* = 3.6 Hz, 1H); 6.5 (d, *J* = 3.2 Hz, 1H); 4.72 (s, 2H); and 2.59 (s, 3H).

N-(4-formylphenyl)-5-(hydroxymethyl)furan-2-carboxamide (**35**).

To a solution of the aldehyde **33** (100 mg, 0.35 mmol, 1 eq) in MeOH (3.5 mL), NH_3_-MeOH 7N (14.50 mL) was added. The resulting mixture was stirred at RT for 1.5 h under a nitrogen atmosphere. Then, the reaction mixture was concentrated under vacuum conditions and purified by flash chromatography using an ISOLUTE Si II 5 g cartridge (Hexane/EtOAc gradient from 4:1 to 1:2 *v*/*v*) to give pure compound **35** as a white solid (51.9 mg). Yield: 61%. ^1^H NMR (400 MHz, DMSO-*d*_6_) δ: 10.47 (s, 1H); 9.90 (s, 1H), 8.00–7.98 (m, 2H); 7.91–7.89 (m, 2H); 7.38 (d, *J* = 3.6 Hz, 1H); 6.54 (d, *J* = 3.6 Hz, 1H); 5.47 (t, *J* = 5.6 Hz, 1H); and 4.51 (d, *J* = 5.6 Hz, 1H).

4-(2-ethoxy-2-oxoethyl)benzoic acid (**37**).

To a solution of commercial 4-carboxyphenylacetic acid **36** (150 mg, 0.83 mmol, 1 eq) in toluene (1.85 mL) and EtOH (0.15 mL), SOCl_2_ (0.15 mL) was added dropwise under a nitrogen atmosphere. The reaction mixture was stirred at 80 °C overnight. The next day, the reaction mixture appeared as a suspension, and EtOH was added in 0.2 mL portions until the solution was clear. To obtain the pure product **37** (150.1 mg), the resulting crude was concentrated under vacuum conditions and purified by trituration with *n*-hexane. Yield: 87%. ^1^H NMR (400 MHz, DMSO-*d*_6_) δ: 12.92 (s, 1H), 7.90–7.88 (m, 2H), 7.40–7.38 (m, 2H), 4.08 (q, *J* = 7.2 Hz, 2H), 3.76 (s, 2H), and 1.18 (t, *J* = 7.2 Hz, 3H).

(E, Z)-N’-hydroxyacetimidamide (**38**).

The title compound was afforded as described in Fortuna et al. [31]. Yield: 57%. ^1^H NMR (400 MHz, DMSO-*d*_6_) δ: 8.60 (br s, 1H), 5.34 (br s, 2H), and 1.61 (s, 3H).

Ethyl 2-(4-(3-methyl-1,2,4-oxadiazol-5-yl)phenyl)acetate (**39**).

Compound **37** (429 mg, 2.06 mmol, 1 eq) was dissolved in DCM (2.75 mL) and added to SOCl_2_ (0.45 mL, 4.12 mmol, 3 eq) under a nitrogen atmosphere. The reaction mixture was stirred at RT for 4 h and concentrated under a nitrogen flux. Then the resulting crude was diluted with acetone and added dropwise to a solution of amidoxime **38** (167.7 mg, 2.27 mmol, 1.1 eq) and K_2_CO_3_ (569 mg, 4.12 mmol, 2 eq) in acetone (3 mL). The resulting mixture was stirred at RT overnight under a nitrogen atmosphere, and then the solvents were removed under vacuum conditions. In order to remove any remaining salt, the crude was treated with H_2_O, and the solid was filtrated under vacuum conditions and subsequentially warmed up to 130 °C solvent-free for 3 h. The crude was purified by flash chromatography using an ISOLUTE Si II 20 g cartridge (petroleum ether/EtOAc gradient from 30:1 to 15:1 *v*/*v*) to give pure compound **39** as a white solid (181 mg). Yield: 36%. ^1^H NMR (400 MHz, DMSO-*d*_6_) δ: 8.08–8.06 (m, 2H), 7.46–7.44 (m, 2H), 4.17 (q, *J* = 7.2 Hz, 2H), 3.69 (s, 2H), 2.47 (s, 3H), and 1.26 (t, *J* = 7.2 Hz, 3H).

Ethyl 2-(4-cyanophenyl)acetate (**43**).

Commercially available 2-(4-cyanophenyl)acetic acid (**42**) (100 mg, 0.62 mmol, 1 eq) was first dissolved in EtOH (0.6 mL) and then added to SOCl_2_ (0.06 mL, 0.81 mmol, 1.3 eq). The reaction was stirred at 65 °C for 2 h and subsequentially evaporated. Purification of the crude was achieved by trituration with *n*-hexane, obtaining pure **43** as a white solid (113.3 mg). Yield: 97%. ^1^H NMR (400 MHz, CDCl_3_) δ: 7.80–7.78 (m, 2H); 7.49–7.47 (m, 2H); 4.08 (q, *J* = 7.2 Hz, 2H); 3.80 (s, 2H); and 1.18 (t, *J* = 6.8 Hz, 3H).

Ethyl 2-(4-(1H-tetrazol-5-yl)phenyl)acetate (**44**).

To a solution of compound **43** (113 mg, 0.6 mmol, 1 eq) in THF dry (1 mL), azido trimethyltin (IV) (124.71 mg, 0.6 mmol, 1 eq) was added under a nitrogen atmosphere. The reaction mixture was maintained at 70 °C for 3 days. After that, the solution was diluted with HCl 1N (25 mL) and extracted with EtOAc (3 × 25 mL). The organic phases were then dried over Na_2_SO_4_, filtrated, and evaporated under vacuum conditions. The resulting crude was purified by flash chromatography using an ISOLUTE Si II 5 g cartridge (*n*-hexane/EtOAc in a gradient from 40:1 to 1:1 *v*/*v*) to give the desired product **44** as a white solid (96.1 mg). Yield: 69%. ^1^H NMR (400 MHz, DMSO-*d*_6_) δ: 7.99–7.97 (m, 2H); 7.51–7.49 (m, 2H); 4.08 (q, *J* = 7.2 Hz, 2H); 3.78 (s, 2H); and 1.19 (t, *J* = 7.2 Hz, 3H). 

#### 3.1.2. General Procedure to Synthesize Hydrazides **29**, **40**–**41**, and **45**

Hydrazine hydrate (10 eq) was added to a solution of ethyl esters **28**, **37**, **39**, and **44** (1 eq) in EtOH (3 mL/mmol). The resulting mixture was stirred at 80 °C for 4–6 h under a nitrogen atmosphere and evaporated. The crude purification was conducted in different ways, depending on the substrate.

2-(4-nitrophenyl) aceto-hydrazide (**29**).

The title compound was synthesized as described in the general procedure, starting with commercial ethyl 2-(4-nitrophenyl) acetate **28** (93.3 mg, 0.40 mmol) and stirring the solution for 4 h. The resulting mixture was purified by crystallization in MeOH at 0 °C. The crystals were dissolved in EtOAc and then washed with H_2_O (1 × 50 mL). The organic phases were combined, dried over Na_2_SO_4_, filtrated, and evaporated. The pure product **29** was achieved as a white solid (134.8 mg). Yield: 82%. ^1^H NMR (400 MHz, DMSO-*d*_6_) δ: 9.31 (s, 1H); 8.19–8.17 (m, 2H); 7.54–7.52 (m, 2H); 4.25 (d, *J* = 3.6 Hz, 2H); and 3.52 (s, 2H).

4-(2-hydrazineyl-2-oxoethyl)benzoic acid (**40**).

The title compound was synthesized as reported in the general procedure, starting with commercial ethyl 2-(4-nitrophenyl) acetate **37** (250 mg, 1.20 mmol) and stirring the solution for 6 h. The resulting crude was purified by flash chromatography (Isolera Biotage automated chromatographer) using SFÄR HC Duo 10g (gradient CH_2_Cl_2_/MeOH 25:1 *v*/*v*), yielding pure compound **40** (148 mg). Yield: 64%. ^1^H NMR (400 MHz, DMSO-*d*_6_) δ: 9.25 (br s, 1H); 7.87–7.85 (m, 2H); 7.37–7.35 (m, 2H); and 3.42 (s, 2H).

2-(4-(3-methyl-1,2,4-oxadiazol-5-yl)phenyl)acetohydrazide (**41**).

The title compound was synthesized as reported in the general procedure, starting with the commercial compound **39** (45 mg, 0.18 mmol) and stirring the solution for 3 h. After evaporation of the solvent, the pure product **41** was achieved as a yellow solid (33.4 mg) without any further purification. Yield: 89%. ^1^H NMR (400 MHz, DMSO-*d*_6_) δ: 9.30 (s, 1H); 8.03–8.01 (m, 2H); 7.51–7.49 (m, 2H); 4.27 (brs, 1H); 3.47 (s, 2H); and 2.41 (s, 3H).

5-(4-(2-(Hydrazineyloxy)-2-oxoethyl)phenyl)-1H-tetrazole (**45**).

The title compound was synthesized as previously reported in the general procedure, starting with the derivative **44** (93.3 mg, 0.40 mmol) and stirring the solution for 4 h. The crude was purified by flash chromatography using an ISOLUTE C18 5 g cartridge (H_2_O/MeOH in a gradient of 8:1 to 1:10 *v*/*v*) to obtain pure compound **45** (26.3 mg) as a white solid. Yield: 30%. ^1^H NMR (400 MHz, DMSO-*d*_6_) δ: 9.27 (s, 1H); 7.95–7.93 (m, 2H); 7.43–7.41 (m, 2H); and 3.42 (s, 2H). 

#### 3.1.3. General Procedure to Synthesize *N*-Acyl Hydrazones **13**, **13a**–**l**, **13n**, and **47**–**48**

Hydrazides **29**, **40**–**41**, and **45** (1 eq) were dissolved in EtOH (7.75 mL/mmol), and carbonyl derivatives **25**–**27**, **34**–**35**, and **46** (1 eq) were added under a nitrogen atmosphere. The reaction was heated at 80 °C for 1–72 h, then cooled to room temperature and concentrated in vacuo. The crude was dissolved in EtOH, and the pure desired products **13**, **13a**–**l**, **13n**, and **47**–**48** were afforded as solids by crystallization in EtOH at 0 °C.

(E)-5-bromo-N-(4-(1-(2-(2-(4-nitrophenyl)acetyl)hydrazineylidene)ethyl)phenyl)furan-2-carboxamide (**13**).

The title compound was synthesized as reported in the general procedure, starting with hydrazide **29** (95 mg, 0.487 mmol) and ketone **25** (150 mg). The reaction was stirred overnight. Pure product **13** was collected by crystallization as an orange solid (203 mg). Yield: 86%. Mp: 206–209 °C. ^1^H NMR (400 MHz, DMSO-*d*_6_) δ: 10.71 _sp_, 10.64 _ap_ (2s, 1H); 10.35 _(sp+ap)_ (s, 1H); 8.22–8.18 _(sp+ap)_ (m, 2H); 7.82–7.78 _(sp+ap)_ (m, 4H); 7.63–7.57 _(sp+ap)_ (m, 2H); 7.40 _(sp+ap)_ (d, *J* = 3.6 Hz, 1H); 6.85 _(sp+ap)_ (d, *J* = 3.6 H z, 1H); 4.23 _sp_, 3.87 _ap_ (2s, 2H); 2.24 _ap_, 2.22 _sp_ (2s, 3H). ^13^C NMR (100 MHz, DMSO-*d*_6_) δ: 172.6; 155.6; 149.5; 146.8; 145.0; 139.8; 133.8; 131.4; 130.9; 130.0; 127.3; 127.1; 125.1; 123.9; 123.7; 120.4; 118.9; 117.8; 114.8; 79.6; 41.5; 13.9. HRMS (ESI, *m*/*z*) calculated for C_21_H_17_BrN_4_O_5_ [M−H]^−^ 483.03096; found: 483.03085.

(E)-N-(4-(1-(2-(2-(4-nitrophenyl)acetyl)hydrazineylidene)ethyl)benzyl)-5-sulfamoylfuran-2-carboxamide (**13a**).

The title compound was synthesized as reported in the general procedure, starting with hydrazide **29** (19 mg, 0.17 mmol) and aldehyde **27** (28 mg). The reaction was stirred for 5 h. Pure product **13a** was collected by crystallization as a slightly pink powder (21.2 mg). Yield: 45%. Mp: 255–258 °C. ^1^H NMR (400 MHz, DMSO-*d*_6_) δ: 10.70 _sp_, 10.64 _ap_ (2s, 1H); 10.47 _(sp+ap)_ (s, 1H); 8.22–8.18 _(sp+ap)_ (m, 2H); 7.98 _(sp+ap)_ (s, 2H); 7.84–7.77 _(sp+ap)_ (m, 4H); 7.63–7.58 _(sp+ap)_ (m, 2H); 7.51 _(sp+ap)_ (d, *J* = 3.6 Hz, 1H); 7.14 _(sp+ap)_ (d, *J* = 3.6 H z, 1H); 4.23 _sp_, 3.87 _ap_ (2s, 2H); 2.31 _ap_, 2.25 _sp_ (2s, 3H). ^13^C NMR (100 MHz, DMSO-*d*_6_) δ: 172.6; 166.4; 155.9; 154.2; 152.1; 148.8; 147.8; 146.8; 146.7; 144.8; 144.7; 139.6; 139.4; 134.2; 134.1; 131.4; 131.0; 127.4; 127.1; 123.9; 123.7; 120.5; 120.3; 115.3; 114.6; 41.0; 14.5; 14.0. HRMS (ESI, *m*/*z*) calculated for C_21_H_19_N_5_O_7_S [M−H]^−^: 484.09324; found: 484.09366.

(E)-5-(hydroxymethyl)-N-(4-(1-(2-(2-(4nitrophenyl)acetyl)hydrazineylidene)ethyl) phenyl) furan-2-carboxamide (**13b**).

The title compound was synthesized as reported in the general procedure, starting with hydrazide **29** (33.2 mg, 0.17 mmol) and ketone **34** (44 mg). The reaction was stirred overnight. Pure product **13b** was collected by crystallization as a light orange solid (57 mg). Yield: 77%. Mp: 238–241 °C. ^1^H NMR (400 MHz, DMSO-*d*_6_) δ: 10.67 _sp_, 10.61 _ap_ (2s, 1H); 10.20 _(sp+ap)_ (s, 1H); 8.21–8.16 _(sp+ap)_ (m, 2H); 7.79–7.77 _(sp+ap)_ (m, 4H); 7.62–7.56 _(sp+ap)_ (m, 2H); 7.30 _(sp+ap)_ (d, *J* = 3.6 Hz, 1H); 6.50 _(sp+ap)_ (d, *J* = 3.6 H z, 1H); 5.43 _(sp+ap)_ (t, *J* = 6 Hz, 1H); 4.48 _(sp+ap)_ (d, *J* = 6 Hz, 2H); 4.22 _sp_, 3.85 _ap_ (2s, 2H); 2.29 _ap_, 2.23 _sp_ (2s, 3H). ^13^C NMR (100 MHz, DMSO-*d*_6_) δ: 172.2; 165.9; 158.7; 156.3; 151.9; 147.5; 146.4; 146.2; 144.4; 144.2; 139.7; 139.5; 133.2; 133.1; 131.0; 130.5; 129.3; 126.9; 126.6; 123.4; 123.2; 119.8; 119.7; 119.4; 115.7; 109.0; 55.9; 40.6; 14.1; 13.5. HRMS (ESI, *m*/*z*) calculated for C_22_H_20_N_4_O_6_ [M−H]^−^: 435.13101; found: 435.13129.

(E)-5-bromo-N-(4-((2-(2-(4-nitrophenyl)acetyl)hydrazineylidene)methyl)phenyl)furan-2-carboxamide (**13c**).

The title compound was synthesized as reported in the general procedure, starting with hydrazide **29** (150 mg, 0.51 mmol) and aldehyde **26** (100 mg). The reaction was stirred for 5 h at 80 °C. Pure product **13c** was collected by crystallization as a yellowish solid (237.1 mg). Yield: 98%. Mp: 277–279 °C. ^1^H NMR (400 MHz, DMSO-*d*_6_) δ: 11.64 _ap_, 11.46 _sp_ (2s, 1H); 10.38 _(sp+ap)_ (s, 1H); 8.22–8.18 _(sp+ap)_ (m, 3H); 7.98 _(sp+ap)_ (s, 1H); 7.83–7.81 _(sp+ap)_ (m, 2H); 7.70–7.66 _(sp+ap)_ (m, 2H); 7.61–7.59 _(sp+ap)_ (m, 2H); 7.40 _(sp+ap)_ (d, *J* = 3.6 Hz, 1H); 6.84 _(sp+ap)_ (d, *J* = 3.6 Hz, 1H); 4.18 _sp_, 3.73 _ap_ (2s, 2H). ^13^C NMR (400 MHz, DMSO-*d*_6_) δ: 171.2; 165.3; 155.1; 149.1; 149.0; 146.5; 146.4; 146.2; 144.1; 143.7; 142.9; 140.0; 139.8; 130.9; 130.5; 129.6; 127.7; 127.4; 125.6; 123.4; 123.2; 120.3; 117.5; 114.4; 40.8. HRMS (ESI, *m*/*z*) calculated for C_20_H_15_BrN_4_O_5_ [M−H]^−^: 469.01475; found: 469.01541.

(E)-5-(hydroxymethyl)-N-(4-((2-(2-(4-nitrophenyl)acetyl)hydrazineylidene)methyl)phenyl) furan-2-carboxamide (**13d**).

The title compound was synthesized as previously reported in the general procedure, starting from hydrazide **29** (41.3 mg, 0.21 mmol) and aldehyde **35** (51.45 mg). The reaction was stirred overnight. Pure product **13d** was collected by crystallization as a light-yellow solid (85.4 mg). Yield: 95%. Mp: 246–248 °C. ^1^H NMR (400 MHz, DMSO-*d*_6_) δ: 11.64 _ap_, 11.47 _sp_ (2s, 1H); 10.26 _(sp+ap)_ (s, 1H); 8.22–8.17 _(sp+ap)_ (m, 3H); 8.18 _(sp+ap)_ (s, 1H); 7.97 _(sp+ap)_ (s, 1H); 7.85–7.83 _(sp+ap)_ (m, 2H); 7.69–7.65 _(sp+ap)_ (m, 2H); 7.61–7.59 _(sp+ap)_ (m, 2H); 7.32 _(sp+ap)_ (d, *J* = 3.2 Hz, 1H); 6.52 _(sp+ap)_ (d, *J* = 3.2 Hz, 1H); 5.45 _(sp+ap)_ (t, *J* = 6 Hz, 1H); 4.50 _(sp+ap)_ (d, *J* = 6 Hz, 2H); 4.18 _sp_, 3.73 _ap_ (2s, 2H). ^13^C NMR (100 MHz, DMSO-*d*_6_) δ: 171.6; 165.8; 159.2; 156.8; 147.1; 146.9; 146.7; 144.6; 144.2; 143.5; 140.8; 140.6; 131.4; 131.0; 130.8; 129.8; 128.1; 127.9; 123.9; 123.8; 123.7; 120.6; 116.2; 109.5; 56.3; 41.3. HRMS (ESI, *m*/*z*) calculated for C_21_H_18_N_4_O_6_ [M−H]^−^: 421.11536; found: 421.11545.

(E)-5-bromo-N-(4-(1-(2-(2-(4-(3-methyl-1,2,4-oxadiazol-5-yl)phenyl)acetyl)hydrazineylidene)ethyl)phenyl)furan-2-carboxamide (**13e**).

The title compound was synthesized as reported in the general procedure, starting with hydrazide **41** (45 mg, 0.22 mmol) and ketone **25** (67 mg). The reaction mixture was stirred overnight. Pure product **13e** (49.4 mg) was afforded as a white solid by crystallization in EtOH at 0 °C. Yield: 43%. Mp: 252–255 °C. ^1^H NMR (400 MHz, DMSO-*d*_6_) δ: 10.66 _sp_, 10.62 _ap_ (2s, 1H); 10.34 _(sp+ap)_ (s, 1H); 8.06–8.01 _(sp+ap)_ (m, 2H); 7.78–7.72 _(sp+ap)_ (m, 5H); 7.58–7.53 _(sp+ap)_ (m, 2H); 7.39 _(sp+ap)_ (d, *J* = 3.6 Hz, 1H); 6.84 _(sp+ap)_ (d, *J* = 3.2 Hz, 1H); 4.17 _sp_, 3.81 _ap_ (2s, 2H); 2.40 _(sp+ap)_ (s, 3H); 2.29 _ap_, 2.23 _sp_ (2s, 3H). ^13^C NMR (100 MHz, DMSO-*d*_6_) δ: 175.2; 173.2; 172.9; 168.1; 155.5; 152.1; 149.6; 147.7; 142.2; 139.6; 133.9; 131.2; 129.8; 128.1; 127.9; 126.0; 122.2; 120.4; 117.8; 114.8; 55.4; 41.3; 14.5; 13.9; 11.7. HRMS (ESI, *m*/*z*) calculated for C_24_H_20_BrN_5_O_4_ [M−H]^−^: 520.06259; found: 520.06262.

(E)-5-(hydroxymethyl)-N-(4-(1-(2-(2-(4-(3-methyl-1,2,4-oxadiazol-5-yl)phenyl)acetyl)hydrazineylidene)ethyl)phenyl)furan-2-carboxamide (**13f**).

The title compound was synthesized as reported in the general procedure, starting with hydrazide **41** (40 mg, 0.196 mmol) and ketone **34** (50 mg). The reaction mixture was stirred for 3 days. Pure product **13f** (73 mg) was afforded as a white solid by crystallization in EtOH at 0 °C. Yield: 80%. Mp: 243–246 °C. ^1^H NMR (400 MHz, DMSO-*d*_6_) δ: 10.64 _sp_, 10.61 _ap_ (2s, 1H); 10.21 _(sp+ap)_ (s, 1H); 8.07–8.02 _(sp+ap)_ (m, 2H); 7.80–7.78 _(sp+ap)_ (m, 5H); 7.60–7.54 _(sp+ap)_ (m, 2H); 7.31 _(sp+ap)_ (d, *J* = 3.2 Hz, 1H); 6.51 _(sp+ap)_ (d, *J* = 3.2 Hz, 1H); 5.43 _(sp+ap)_ (t, *J* = 6 Hz, 1H); 4.50 _(sp+ap)_ (d, *J* = 6 Hz, 2H); 4.20 _sp_, 3.81 _ap_ (2s, 2H); 2.41 _(sp+ap)_ (s, 3H); 2.30 _ap_, 2.24 _sp_ (2s, 3H). ^13^C NMR (100 MHz, DMSO-*d*_6_) δ: 174.7; 172.4; 167.6; 166.2; 158.6; 156.3; 147.3; 146.3; 141.7; 139.4; 133.2; 130.7; 130.3; 127.7; 126.8; 126.5; 121.7; 121.58; 119.8; 115.6; 109.0; 55.9; 40.8; 14.0; 13.5; 11.25. HRMS (ESI, *m*/*z*) calculated for C_25_H_23_N_5_O_5_ [M−H]^−^: 472.16264; found: 472.16287.

(E)-5-(hydroxymethyl)-N-(4-((2-(2-(4-(3-methyl-1,2,4-oxadiazol-5-yl)phenyl)acetyl) hydrazono)methyl)phenyl)furan-2-carboxamide (**13g**).

The title compound was synthesized as reported in the general procedure, starting with hydrazide **41** (26.4 mg, 0.129 mmol) and aldehyde **35** (31.7 mg). The reaction mixture was stirred at 80 °C for 1.5 h. Pure product **13g** (38.1 mg) was afforded as a yellow solid by crystallization in EtOH at 0 °C. Yield: 57%. Mp: 232–235 °C. ^1^H NMR (400 MHz, DMSO-*d*_6_) δ: 11.64 _ap_, 11.44 _sp_ (2s, 1H), 10.26 _(sp+ap)_ (s, 1H); 8.17 _(sp+ap)_ (s, 1H); 8.07–8.03 _(sp+ap)_ (m, 3H); 7.97 _(sp+ap)_ (s, 1H); 7.85–7.83_(sp+ap)_ (m, 2H); 7.69–7.65 _(sp+ap)_ (m, 2H); 7.57–7.55 _(sp+ap)_ (m, 2H); 7.32 _(sp+ap)_ (d, *J* = 2.8 Hz, 1H); 6.52 _(sp+ap)_ (d, *J* = 2.8 Hz, 1H);0 5.46 _(sp+ap)_ (brs, 1H); 4.50 _(sp+ap)_ (s, 2H); 4.13 _sp_, 3.67 _ap_ (2s, 2H); 2.41 _(sp+ap)_ (s, 3H). ^13^C NMR (100 MHz, DMSO-*d*_6_) δ: 175.2; 175.1; 171.9; 168.1; 166.1; 159.2; 156.8; 147.0; 146.7; 143.4; 142.0; 141.7; 140.8; 140.6; 131.1; 130.7; 129.8; 128.2, 128.1; 127.8; 122.3, 122.1; 120.7; 120.6; 116.2; 109.5; 56.3; 41.6; 11.7. HRMS (ESI, *m*/*z*) calculated for C_24_H_21_N_5_O_5_ [M−H]^−^: 458.14699; found: 458.14728.

(E)-4-(2-(2-(1-(4-(5-bromofuran-2-carboxamido)phenyl)ethylidene)hydrazineyl)-2-oxoethyl)benzoic acid (**13h**).

The title compound was synthesized as reported in the general procedure, starting with hydrazide **40** (50 mg, 0.257 mmol) and ketone **25** (79.4 mg). The reaction mixture was stirred overnight. Pure product **13h** (106 mg) was afforded as a yellowish solid by crystallization in EtOH at 0 °C. Yield: 85%. Mp: 292–295 °C. ^1^H NMR (400 MHz, DMSO-*d*_6_) δ: 12.82 _(sp+ap)_ (brs, 1H); 10.62 _sp_, 10.59 _ap_ (2s, 1H); 10.34 _(sp+ap)_ (s, 1H); 7.96–7.87 _(sp+ap)_ (m, 2H); 7.79–7.78 _(sp+ap)_ (m, 3H); 7.46–7.39 _(sp+ap)_ (m, 2H); 6.87–6.84 _(sp+ap)_ (m, 1H); 4.13 _sp_, 3.79 _ap_ (2s, 2H); 2.29 _ap_, 2.23 _sp_ (2s, 3H). ^13^C NMR (100 MHz, DMSO-*d*_6_) δ: 172.6; 167.3; 166.4; 155.1; 151.6; 149.2; 147.2; 141.3; 139.3; 133.5; 129.7; 129.3; 128.9; 126.8; 126.6; 125.5; 119.9; 117.4; 114.4; 40.80; 14.0; 13.5. HRMS (ESI, *m*/*z*) calculated for C_22_H_18_BrN_3_O_5_ [M−H]^−^: 482.03571; found: 482.03586.

(E)-4-(2-(2-(1-(4-(5-(hydroxymethyl)furan-2-carboxamido)phenyl)ethylidene)hydrazineyl)-2-oxoethyl)benzoic acid (**13i**).

The title compound was synthesized as reported in the general procedure, starting with hydrazide **40** (50 mg, 0.257 mmol) and ketone **34** (79.4 mg). The reaction mixture was stirred for 3 h. Pure product **13i** was afforded as a solid by crystallization in EtOH at 0 °C. Yield: 62%. Mp: 232–235 °C. ^1^H NMR (400 MHz, DMSO-*d*_6_) δ: 10.56 _(sp+ap)_ (s, 1H); 10.20 _(sp+ap)_ (s, 1H); 7.94–7.76 _(sp+ap)_ (m, 5H); 7.43–7.31_(sp+ap)_ (m, 3H); 6.53–6.51 _(sp+ap)_ (m, 1H); 5.43 _(sp+ap)_ (brs. 1H); 4.50 _(sp+ap)_ (s, 2H); 4.11 _sp_, 3.74 _ap_ (2s, 2H); 2.29 _ap_, 2.23 _sp_ (2s, 3H). ^13^C NMR: (100 MHz, DMSO-*d*_6_) δ: 173.0; 159.1; 157.9; 156.7; 147.7; 146.8; 141.7; 140.6; 140.1; 139.9; 133.7; 130.6; 130.1; 129.7; 127.5; 127.0; 126.9; 120.2; 120.1; 116.1; 109.5; 56.4; 14.5. HRMS (ESI, *m*/*z*) calculated for C_23_H_21_N_3_O_6_ [M−H]^−^: 434.13576; found: 434.13580.

(E)-4-(2-(2-(4-(5-bromofuran-2-carboxamido)benzylidene)hydrazineyl)-2-oxoethyl)benzoic acid (**13l**).

The title compound was synthesized as reported in the general procedure, starting with hydrazide **40** (43.5 mg, 0.224 mmol) and aldehyde **27** (65.5 mg). The reaction mixture was stirred overnight. Pure product **13l** (89.2 mg) was afforded as a yellowish solid by crystallization in EtOH at 0 °C. Yield: 85%. Mp: 307–310 °C. ^1^H NMR (400 MHz, DMSO-*d*_6_) δ: 12.86 _(sp+ap)_ (brs, 1H); 11.61 _ap_, 11.41 _sp_ (2s, 1H); 10.38 _(sp+ap)_ (s, 1H); 8.17 _ap_, 7.96 _sp_ (2s, 1H); 7.91–7.81 _(sp+ap)_ (m, 5H); 7.69–7.65 _(sp+ap)_ (m, 2H); 7.44–7.40 _(sp+ap)_ (m, 3H); 6.85–6.84 _(sp+ap)_ (m, 1H); 4.07 _sp_, 3.62 _ap_ (2s, 2H).^13^C NMR (100 MHz, DMSO-*d*_6_) δ: 171.6; 167.2; 165.8; 155.1; 149.1; 141.1; 140.9; 140.0; 139.8; 129.7; 129.3; 128.9; 127.6; 127.4; 125.6; 120.3; 117.5; 114.4; 41.1. HRMS (ESI, *m*/*z*) calculated for C_21_H_16_BrN_3_O_5_ [M−H]^−^: 468.02006; found: 468.02017.

(E)-N-(4-((2-(2-(4-(1H-tetrazol-5-yl)phenyl)acetyl)hydrazineylidene)methyl)phenyl)-5-bromofuran-2-carboxamide (**13n**).

The title compound was synthesized as reported in the general procedure, starting with hydrazide **45** (22.8 mg, 0.1045 mmol) and aldehyde **27** (30.7 mg). The reaction mixture was stirred overnight. Pure product **13n** (37.4 mg) was afforded as a yellowish solid by crystallization in EtOH at 0 °C. Yield: 73%. Mp: 262–265 °C. ^1^H NMR (400 MHz, DMSO-*d*_6_) δ: 11.61 _ap_, 11.40 _sp_ (2s, 1H); 10.37 _(sp+ap)_ (s, 1H); 8.19 _ap_, 8.00 _sp_ (2s, 1H); 8.00–7.96 _(sp+ap)_ (m, 3H); 7.84–7.81 _(sp+ap)_ (m, 2H); 7.70–7.66 _(sp+ap)_ (m, 2H); 7.54–7.52 _(sp+ap)_ (m, 2H); 7.40 _(sp+ap)_ (d, *J* = 3.2 Hz, 1H); 6.85 40 _(sp+ap)_ (d, *J* = 3.2 Hz, 1H); 4.09 _sp_, 3.64 _ap_ (2s, 2H). ^13^C NMR (100 MHz, DMSO-*d*_6_) δ: 171.7; 165.9; 155.5; 155.1; 149.1; 146.3; 142.7; 139.9; 139.7; 138.9; 138.7; 130.4; 130.0; 129.7; 129.6; 127.6; 127.3; 127.1; 126.7; 125.5; 123.2; 122.8; 120.3; 119.9; 117.4; 114.3; 41.0. HRMS (ESI, *m*/*z*) calculated for C_21_H_16_N_7_O_3_Br [M−H]^−^: 492.04252; found: 492.04276.

(E)-4-(2-(2-(4-(((tert-butoxycarbonyl)amino)methyl)benzylidene)hydrazineyl)-2-oxoethyl)benzoic acid (**47**).

The title compound was synthesized as reported in the general procedure, starting with hydrazide **40** (57.8 mg, 0.30 mmol) and aldehyde **46** (70 mg). The reaction was stirred overnight. Pure product **47** was collected by crystallization as a white solid (86.7 mg). Yield: 70%. ^1^H NMR (400 MHz, DMSO-*d*_6_) δ: 13.00 _(sp+ap)_ (brs, 1H); 11.59 _ap_, 11.39 _sp_ (2s, 1H); 8.19 _sp_, 7.98 _ap_ (2s, 1H); 7.91–7.86 _(sp+ap)_ (m, 2H); 7.64–7.62 _(sp+ap)_ (m, 2H); 7.43–7.41 _(sp+ap)_ (m, 3H); 7.30–7.28 _(sp+ap)_ (m, 2H); 4.14 _(sp+ap)_ (d, *J* = 5.6 Hz, 2H); 4.07 _sp_, 3.63 _ap_ (2s, 2H); 1.39 _(sp+ap)_ (s, 9H).

(tert-butyl (E)-(4-((2-(2-(4-(1H-tetrazol-5 yl)phenyl)acetyl)hydrazineylidene)methyl)benzyl) carbamate (**48**).

The title compound was synthesized as reported in the general procedure, starting with hydrazide **45** (80 mg, 0.37 mmol) and aldehyde **46** (86 mg). The reaction was stirred overnight. Since crystallization failed, the crude compound was purified by flash chromatography (gradient CHCl_3_/MeOH 10:1 to 8:1 *v*/*v*), yielding pure **48** (71 mg) as a yellow solid. Yield: 44%. ^1^H NMR (400 MHz, DMSO-*d*_6_) δ: 11.64 _ap_, 11.44 _sp_ (2s, 1H); 8.32 _sp_, 8.19 _ap_ (2s, 1H); 7.99–7.94 _(sp+ap)_ (m, 2H); 7.66–7.62 _(sp+ap)_ (m, 2H); 7.51–7.44 _(sp+ap)_ (m, 3H); 7.30–7.28 _(sp+ap)_ (m, 2H); 4.14 _(sp+ap)_ (d, *J* = 5.6 Hz, 2H); 4.07 _sp_, 3.63 _ap_ (2s, 2H); 1.39 _(sp+ap)_ (s, 9H).

(E)-(4-((2-(2-(4-carboxyphenyl)acetyl)hydrazineylidene)methyl)phenyl)methanaminium 2,2,2-trifluoroacetate (**49**).

A suspension of **47** (139 mg, 0.338 mmol, 1 eq) in DCM dry (5.6 mL) was cooled at 0 °C and added to TFA (0.039 mL, 5.07 mmol, 15 eq). The reacting solution was stirred at RT for 2 h. Then, the reacting mixture was co-evaporated with toluene in vacuo to achieve pure compound **49** as a white solid without any further purification. Yield: quantitative. ^1^H NMR (400 MHz, DMSO-*d*_6_) δ: 12.90 _(sp+ap)_ (brs, 1H); 11.71 _ap_, 11.49 _sp_ (2s, 1H); 8.20 _(sp+ap)_ (brs, 3H); 8.00 _sp_, 7.91 _ap_ (2s, 1H); 7.89–7.86 _(sp+ap)_ (m, 2H); 7.75–7.73 _(sp+ap)_ (m, 2H); 7.52–7.50 _(sp+ap)_ (m, 2H); 7.45–7.42 _(sp+ap)_ (m, 2H); 4.09–4.08 _(sp+ap)_ (m, 3H); 3.63 _ap_ (s, 2H). ^19^F (376 MHz, DMSO-*d*_6_) δ: −73.46.

(E)-(4-((2-(2-(4-(1H-tetrazol-5-yl)phenyl)acetyl)hydrazono)methyl)phenyl) methanaminium 2,2,2-trifluoroacetate (**50**).

The title compound was synthesized as reported for compound **49**, starting with derivative **48** (150 mg, 0.478 mmol). After workup, compound **50** was obtained as a white solid (71 mg) without any further purification. Yield: quantitative. ^1^H NMR (400 MHz, DMSO-*d*_6_) δ: 11.80 _ap_, 11.50 _sp_ (2s, 1H); 8.23 _sp_ (1s, 1H); 8.18 _(sp+ap)_ (brs, 3H); 8.01–7.96 _(sp+ap)_ (m, 2H); 7.77–7.71 _(sp+ap)_ (m, 2H); 7.55–7.49 _(sp+ap)_ (m, 3H); 4.11–4.07 _(sp+ap)_ (m, 3H); 3.66 _ap_ (s, 2H). ^19^F (376 MHz, DMSO-*d*_6_) δ: −73.46.

2,5-dioxopyrrolidin-1-yl 5-bromofuran-2-carboxylate (**51**).

To a suspension of commercial compound **21** (300 mg, 1.57 mmol, 1 eq) in DCM (3 mL), *N*-hydroxysuccinimide (271 mg, 2.36 mmol, 1.5 eq) and EDC (451 mg, 2.36 mmol, 1.5 eq) were subsequently added. The reacting mixture was stirred at RT overnight, then diluted with CHCl_3_. The organic phase was washed with water (2 × 50 mL) and a NaHCO_3_-saturated solution (2 × 50 mL). The organic layer was dried over Na_2_SO_4_, filtered, and evaporated under reduced pressure. The obtained crude was purified by flash chromatography (isocratic gradient: CHCl_3_ 100% *v*/*v*) using an ISOLUTE Flash Si II 10 g cartridge to give compound **51** (319 mg) as a white solid. Yield: 71%. ^1^H NMR (400 MHz, CDCl_3_) δ: 7.42 (d, *J* = 3.6 Hz, 1H); 6.57 (d, *J* = 3.6 Hz, 1H); 2.89 (s, 4H). 

#### 3.1.4. General Procedure to Synthesize Final Compounds **13m**,**o**

To a solution of bromofuranosyl derivative 51 (1 eq) in DMF dry (4.2 mL/mmol), DIPEA (2 eq) was added. Lastly, a solution of trifluoroacetate salt 49 or 50 (1 eq) in DMF dry (8.4 mL/mmol) was added dropwise to the reacting mixture. The reaction was stirred for 2 h and then diluted with EtOAc.

(E)-4-(2-(2-(4-((5-bromofuran-2-carboxamido)methyl)benzylidene)hydrazineyl)-2-oxoethyl)benzoic acid (**13m**).

The title compound was synthesized as reported in the general procedure, starting with **51** (34 mg, 0.118 mmol) and trifluoroacetate **49** (50 mg, 0.118 mmol). After dilution with EtOAc (50 mL), the organic phase was washed with water (2 × 50 mL), and brine (1 × 50 mL). The water phase was acidified using HCl 37% until precipitation of a white solid, which was filtered under low pressure to yield pure compound **13m** as a white powder (23 mg). Yield: 40%. Mp: 245–248 °C. ^1^H NMR (400 MHz, DMSO-*d*_6_) δ: 12.82 _(sp+ap)_ (brs, 1H); 11.61 _ap_, 11.42 _sp_ (2s, 1H); 9.02 _(sp+ap)_ (t, *J* = 6 Hz, 1H); 8.19 _ap_, 7.97 _sp_ (2s, 1H); 7.91–7.86 _(sp+ap)_ (m, 2H); 7.66–7.63 _(sp+ap)_ (m, 2H); 7.44–7.41 _(sp+ap)_ (m, 2H); 7.36–7.34 _(sp+ap)_ (m, 2H); 7.18–7.17 _(sp+ap)_ (m, 1H); 6.77–6.76 _(sp+ap)_ (m, 1H); 4.43 _(sp+ap)_ (d, *J* = 6 Hz, 2H); 4.06 _sp_, 3.62 _ap_ (2s, 2H). ^13^C NMR (100 MHz, DMSO-*d*_6_) δ: 171.6; 167.1; 165.8; 156.6; 149.4; 146.4; 142.8; 141.2; 141.0; 140.9; 140.7; 132.7; 129.5; 129.2; 129.1; 129.0; 128.8; 127.6; 127.0; 126.7; 124.4; 115.9; 113.9; 41.7; 41.0. HRMS (ESI, *m*/*z*) calculated for C_22_H_18_N_3_O_5_Br [M−H]^−^: 482.03571, found: 482.03589.

(E)-N-(4-((2-(2-(4-(1H-tetrazol-5-yl)phenyl)acetyl)hydrazineylidene)methyl)benzyl)-5-bromofuran-2-carboxamide (**13o**).

The title compound was synthesized as reported in the general procedure, starting with **51** (48 mg, 0.167 mmol) and trifluoroacetate **50** (75 mg, 0.167 mmol). After dilution with EtOAc (50 mL), the organic phase was washed with water (2 × 50 mL). The water phase was acidified using HCl 37% and extracted with EtOAc (2 × 50 mL). The organic layers were dried over Na_2_SO_4_, filtered, and evaporated under vacuum conditions. The pure final compound **13o** was obtained as a pinkish powder (35 mg). Yield: 41%. Mp: 245–248 °C. ^1^H NMR (400 MHz, DMSO-*d*_6_) δ: 11.65 _ap_, 11.45 _sp_ (2s, 1H); 10.56 _(sp+ap)_ (t, *J* = 5.6 Hz, 1H); 8.19 _ap_ (s, 1H); 8.00–7.95 _(sp+ap)_ (m, 3H); 7.68–7.63 _(sp+ap)_ (m, 2H); 7.53–7.51 _(sp+ap)_ (m, 2H); 7.37–7.35 _(sp+ap)_ (m, 2H); 7.18 _(sp+ap)_ (d, *J* = 3.2 Hz, 1H); 6.77 _(sp+ap)_ (d, *J* = 3.2 Hz, 1H); 4.43 _(sp+ap)_ (d, *J* = 5.6 Hz, 2H); 4.08 _sp_, 3.64 _ap_ (2s, 2H). ^13^C NMR (100 MHz, DMSO-*d*_6_) δ: 172.8; 171.8; 166.1; 156.7; 149.6; 146.5; 142.9; 141.4; 141.1; 138.8; 138.6; 132.9; 130.4; 130.1; 127.8; 127.7; 127.1; 126.8; 124.6; 116.1; 114.0; 41.8; 41.0. HRMS (ESI, *m*/*z*) calculated for C_22_H_17_N_7_O_3_Br [M−H]^−^: 506.05817, found: 506.05801. 

The images of the ^1^H, ^13^C NMR, and HRMS spectra are represented in Supplementary Materials.

### 3.2. Biological Assays

Recombinant human MMP-14 catalytic domain was a kind gift from Prof. Gillian Murphy (Department of Oncology, University of Cambridge, UK). Pro-MMP-1, pro-MMP-2, pro-MMP-9, and pro-MMP-13 were purchased from Merck Millipore (Burlington, MA, USA). *p*-Aminophenylmercuric acetate (APMA) was from Sigma-Aldrich (St. Louis, MO, USA). 

#### 3.2.1. Enzyme Activation

Proenzymes were activated immediately prior to use (APMA 2 mM for 1 h at 37 °C for MMP-2, APMA 2 mM for 2 h at 37 °C for MMP-1, APMA 1 mM for 30 min at 37 °C for MMP-13, and APMA 1 mM for 1 h at 37 °C for MMP-9).

#### 3.2.2. Enzyme Inhibition Assays

For assay measurements, the purchased compound stock solutions (10 mM in DMSO) were further diluted for each MMP in the fluorometric assay buffer (FAB: Tris 50 mM, pH = 7.5, NaCl 150 mM, CaCl_2_ 10 mM, Brij 35 0.05%, and DMSO 1%), following the protocol already reported [41,42]. Activated enzyme (final concentration 0.56 nM for MMP-2, 0.3 nM for MMP-13, 1.3 nM for MMP-9, 1 nM for MMP-14cd, and 2.0 nM for MMP-1) and inhibitor solutions were incubated in the assay buffer for 3 h at 25 °C. After the addition of a 200 μM solution of the fluorogenic substrate Mca-Lys-Pro-Leu-Gly-Leu-Dap(Dnp)-Ala-Arg-NH_2_ (Merck Millipore) in DMSO (final concentration 2 μM), the hydrolysis was monitored every 15 sec for 15 min, recording the increase in fluorescence (λ_ex_ = 325 nm, λ_em_ = 400 nm) using a SpectraMax Gemini XPS (Molecular Devices, Sunnyvale, CA, USA) plate reader. The assays were performed in triplicate with a total volume of 200 μL per well in 96-well microtitre plates (Corning, black, NBS, Turin, Italy). The MMP inhibition activity was expressed in relative fluorescent units (RFU). The percentage of inhibition was calculated from control reactions without the inhibitor. The inhibitory effect of the tested compounds was routinely estimated at a concentration of 100 μM on MMP-13. Those derivatives found to be active were tested at additional concentrations, and IC_50_ was determined using at least five concentrations of the inhibitor causing an inhibition between 10% and 90%, using the formula: V_i_/V_o_ = 1/(1 + [I]/IC_50_), where V_i_ is the initial velocity of substrate cleavage in the presence of the inhibitor at concentration [I] and V_o_ is the initial velocity in the absence of the inhibitor. Results were analyzed using SoftMax Pro software version 5.4.3 (Molecular Devices, Sunnyvale, CA, USA) and Prism Software version 5.0 (GraphPad Software, Inc., La Jolla, CA, USA).

### 3.3. Computational Methods

#### 3.3.1. Shape-Based Similarity

Conformations were generated using Omega [43,44] with default parameters and requiring a maximum of 10 conformations. The co-crystallized inhibitors used as references in the shape comparison corresponded to the ligands of the crystal structures with the following PDB codes: 2OW9 [19], 2OZR [19], 3KEC [20], 3KEJ [20], 3KEK [20], 3WV1 [21], and 5BPA [22]. Shape similarity between the library compounds and the reference compounds was calculated with ROCS [45,46] using the ShapeTanimoto coefficient, a value between 0 and 1 calculated by the following equation:

ShapeTanimoto_f,g_ = O_f,g_/(I_f_ + I_g_ − O_f,g_)

in which the I terms are the self-volume overlaps of each molecule, while the O term is the overlap between the two functions [47].

Conformations with a ShapeTanimoto value lower than 0.5 for any of the reference compounds were discarded.

#### 3.3.2. Ligand Setup for Docking

Before docking, ligand molecules were prepared with LigPrep [48] with default parameter values except for the following options: (a) respect chiralities from input geometry when generating stereoisomers; (b) use Epik [49] for ionization and tautomerization; (c) use 7.0 as an effective pH; and (d) use 2.0 as a pH tolerance for generated structures.

#### 3.3.3. Protein Preparation

After verifying the fitting of the coordinates of the residues in the binding site relative to their corresponding electron density map with VHELIBS, the B chain of the crystal structure with PDB code 3WV1 was prepared by using Maestro’s Protein Preparation Wizard [50] through the following procedure: (a) align to 1ROS, chain A; (b) remove original hydrogens; (c) cap termini; (d) generate ionization and tautomeric states of the ligand with Epik; (e) assign hydrogen bonds at pH 7 with PROPKA; (f) use force field OPLS_2005 to minimize the structure at 0.30 Å; and (g) remove all water molecules from the structure.

#### 3.3.4. Grid Preparation 

The grid for protein-ligand docking was generated with Maestro [51] by using default parameter values except for the following settings: (a) the grid center coordinates were (46.0, 80.0, and −1.0); (b) halogens were included as acceptors; (c) the inner box size was (10, 10, and 10); (d) the outer box size was (30, 30, and 30); (e) hydrogen bond constraints were defined on the backbone nitrogen of the residues Thr245 and Thr247 as well as the side-chain oxygen of the residue Thr245; and (f) two positional constraints with a radius of 2 Å were defined on the coordinates (46.3, 80.1, and −7.5) and (51.2, 80.5, and 4.6), respectively.

#### 3.3.5. Molecular Docking

Protein-ligand docking was performed with Glide [52] by using default parameter values except for the following settings: (a) SP precision; (b) enhance planarity of conjugated π groups; (c) include halogens as acceptors; (d) write out at most 10 poses per ligand; (e) include 50 poses per ligand in post-docking minimization; (g) require the accomplishment of both positional constraints by aromatic atoms; and (f) require the accomplishment of one hydrogen bond constraint. 

The first picture in each panel of Figure 3 was obtained with Maestro [51], and the second and third pictures were obtained with Flare [28]. The docked poses were predicted with GlideXP [52].

## 4. Conclusions

In order to obtain potent and selective MMP-13 inhibitors devoid of a ZBG, we developed a virtual screening workflow aimed at identifying compounds that target the S1′ pocket of MMP-13, a region in the MMP binding site that has been shown to be different for MMP-13 with respect to other MMPs. For this purpose, we first applied a MW filter to discard compounds unlikely to survive subsequent filters. Next, we used a shape-based similarity analysis to restrict the initial library of compounds to those able to adopt the characteristic *U* shape adopted by co-crystallized non-zinc-binding selective MMP-13 inhibitors. Then, we performed protein-ligand docking simulations to predict the binding modes of these compounds. Finally, we analyzed previously reported SAR studies to identify MMP-13 inhibitor interactions with the protein that are important for activity, and we selected the docked poses obtained in the protein-ligand docking according to these criteria. The bioactivity assays on isolated enzymes identified three putative hit compounds capable of inhibiting MMP-13 in the μM range, one of which displayed at least 4-fold selectivity over MMP-1, MMP-2, MMP-9, and MMP-14. Then, a structure-based optimization of the *N*-acyl hydrazone hit compound **13** guided the synthesis of a series of 12 new derivatives. Among these, a carboxylate derivative (**13m**) was found to selectively inhibit MMP-13 with an IC_50_ = 1.8 µM. A docking study showed that the presence of an acidic moiety in R introduced more negative electrostatic surfaces close to the residue Lys140, which resulted in better electrostatic interaction between this compound and the enzyme at the S1″ pocket, thus increasing its activity. The next round of optimization from hit to lead will be necessary to further develop this interesting class of new compounds.

## Figures and Tables

**Table 1:** Structure and MMP-13 inhibitory activity of the selected compounds.

Compound	Specs Code	Inhibition % *^a^* (100 μM)	clogP *^b^*
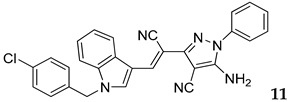	AG-205/37007400	54.2	5.6
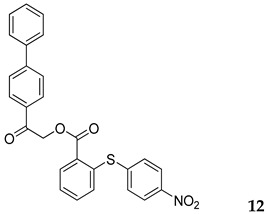	AK-968/13281769	48.7	6.6
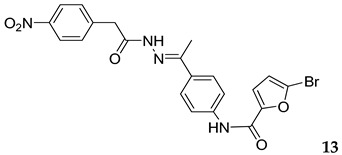	AK-968/15605222	63.1	4.3

*^a^* Percent inhibition of MMP-13 was observed at a 100 µM concentration of the test compounds. Assays were performed in duplicate, % inhibition was determined, and average values are present. *^b^* Calculated the n-octanol/water partition coefficient [28].

**Table 2:** In vitro *^a^* activity (IC_50_ µM values) of the three selected compounds towards different MMPs.

Compound	MMP-1	MMP-2	MMP-9	MMP-13	MMP-14
11	99 ± 8.0	60 ± 4.6	66 ± 4.6	91 ± 8.5	94 ± 5.5
12	75 ± 4	47 ± 1.6	72 ± 4.8	105 ± 5.8	67 ± 4.6
13	91 ± 0.8	99 ± 6	68 ± 3	14.6 ± 1.6	63 ± 5.4

*^a^* Assays were run in triplicate. The final values given here are the mean ± SD of three independent experiments.

**Table 3:** Chemical structure and inhibitory activity of compound **13** and its derivatives **13a**–**o**.

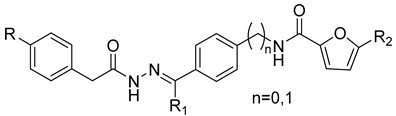					
Compound	R	R_1_	R_2_	n	IC_50_ (µM) *^a^*
**13**	NO_2_	Me	Br	0	14.6 ± 1.6
**13a**	NO_2_	Me	-SO_2_NH_2_	0	>100
**13b**	NO_2_	Me	-CH_2_OH	0	>100
**13c**	NO_2_	H	Br	0	20 ± 1.2
**13d**	NO_2_	H	-CH_2_OH	0	40 ± 3.5
**13e**	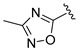	Me	Br	0	>100
13f	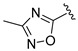	Me	-CH_2_OH	0	120 ± 7.6
**13g**	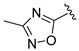	H	-CH_2_OH	0	33 ± 1.9
**13h**	-COOH	Me	Br	0	15 ± 3.6
**13i**	-COOH	Me	-CH_2_OH	0	49 ± 3.6
**13l**	-COOH	H	Br	0	11.4 ± 1.0
**13m**	-COOH	H	Br	1	1.8 ± 0.5
**13n**	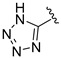	H	Br	0	2.7 ± 0.2
**13o**	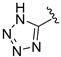	H	Br	1	163 ± 8.0

*^a^* Assays were run in triplicate. The final values given here are the mean ± SD of three independent experiments.

**Table 4:** Absorption, distribution, metabolism, excretion, and toxicity (ADMET) properties of compound **13** and its derivatives **13a**–**o**.

Compound	Accomplish Lipinski’s Rule? *^a^*	LogP *^a^*	Molecular Mass *^a^*	Hydrogen Bond Aceptors (HBA) *^a^*	Hydrogen Bond Donors (HBD) *^a^*	Rotatable Bonds *^a^*	BBB Permeability (Cross if > 0.3 and Poorly if <−1) *^a^*	CNS Permeability (Penetrate > −2 and Unable <−3) *^a^*	CaCo_2_ Permeability (high > 0.90) *^a^*	Intestinal Absorption (Poor < 30%) *^a^*	Skin Permeability (low > −2.5) *^a^*	HERG I Inibition *^a^*	HERG II Inibition *^a^*	Hepatotoxicity *^a^*	Cardiotoxicity *^b^*	Druglikeness (Traded Drugs > 0) *^c^*	Mutagenic *^c^*	Tumorigenic *^c^*	Reproductive Effects *^c^*	Irritant *^c^*
**13**	Yes	4.28550	485.294	7	2	7	−0.648	−2.187	0.630	88.652	−2.745	No	Yes	Yes	Non-cardiotoxic	−3.0971	No	No	No	No
**13a**	Yes	2.17040	485.478	8	3	8	−1.217	−3.044	0.365	71.804	−2.737	No	Yes	Yes	Non-cardiotoxic	−5.8165	No	No	No	No
**13b**	Yes	3.01530	436.424	7	3	8	−0.991	−2.647	0.015	79.570	−2.744	No	Yes	Yes	Non-cardiotoxic	−0.015854	No	No	No	No
**13c**	Yes	3.89540	471.267	6	2	7	−0.670	−2.279	0.631	87.820	−2.746	No	Yes	Yes	Non-cardiotoxic	−2.2885	No	No	No	No
**13d**	Yes	2.62520	422.397	7	3	8	−0.969	−2.738	0.019	78.738	−2.744	No	Yes	Yes	Non-cardiotoxic	0.74757	No	No	No	No
**13e**	Yes	4.73572	522.359	7	2	7	−0.755	−2.229	0.630	94.590	−2.751	No	Yes	Yes	Non-cardiotoxic	2.4995	No	No	No	No
**13f**	Yes	3.67972	474.497	6	4	8	−1.410	−2.724	0.143	77.682	−2.736	No	Yes	Yes	Non-cardiotoxic	1.4725	No	No	No	No
**13g**	Yes	3.28962	460.470	6	4	8	−1.388	−2.815	0.148	76.850	−2.736	No	Yes	Yes	Non-cardiotoxic	2.2082	No	No	No	No
**13h**	Yes	2.74080	483.298	6	2	7	−0.548	−2.553	0.694	63.609	−2.732	No	No	Yes	Non-cardiotoxic	2.0347	No	No	No	No
**13i**	Yes	1.47060	434.428	7	3	8	−0.873	−3.012	0.278	54.527	−2.733	No	No	Yes	Non-cardiotoxic	5.1125	No	No	No	No
**13l**	Yes	2.35070	469.271	6	2	7	−0.569	−2.644	0.695	62.777	−2.732	No	No	Yes	Non-cardiotoxic	2.8355	No	No	No	No
**13m**	Yes	2.02840	483.298	6	2	8	−0.547	−2.786	0.670	60.411	−2.732	No	No	Yes	Weak or moderate	3.1821	No	No	No	No
**13n**	Yes	2.79640	493.301	7	2	7	−1.006	−2.747	0.658	83.391	−2.745	No	Yes	Yes	Weak or moderate	0.87219	No	No	No	No
**13o**	Yes	2.47410	507.328	7	2	8	−0.98	−2.888	0.632	81.025	−2.745	No	Yes	Yes	Weak or moderate	1.2118	No	No	No	No

*^a^* Calculated with pkCSM (https://biosig.lab.uq.edu.au/pkcsm/ and https://pubmed.ncbi.nlm.nih.gov/25860834/ accessed on 19 June 2023). *^b^* Calculated with Pred-hERG 4.2 (www.predherg.labmol.com.br, accessed on 19 June 2023). *^c^* Calculated with DataWarrior 5.5.0 (https://openmolecules.org/datawarrior/ and https://pubmed.ncbi.nlm.nih.gov/25558886/ accessed on 19 June 2023).

**Table 5:** In vitro *^a^* activity (IC_50_ µM values) of **13m** against a panel of MMPs.

Compound	MMP-1	MMP-2	MMP-9	MMP-13	MMP-14
**13m**	177 ± 20	3.6 ± 0.4	50 ± 3.2	1.8 ± 0.5	>200
**13**	91 ± 0.8	99 ± 6	68 ± 3	14.6 ± 1.6	63 ± 5.4

*^a^* Assays were run in triplicate. The final values given here are the mean ± SD of three independent experiments.

## Data Availability

All data are reported in the manuscript and in the Supplementary Material and are available from the corresponding authors upon request.

## Supplementary Materials

The following supporting information can be downloaded at: https://www.mdpi.com/article/10.3390/ijms241311098/s1.

## References

[1] Arabelovic S., McAlindon T.E. (2005). Considerations in the Treatment of Early Osteoarthritis. Curr. Rheumatol. Rep..

[2] Li H., Wang D., Yuan Y., Min J. (2017). New Insights on the MMP-13 Regulatory Network in the Pathogenesis of Early Osteoarthritis. Arthritis Res. Ther..

[3] Takaishi H., Kimura T., Dalal S., Okada Y., D’Armiento J. (2008). Joint Diseases and Matrix Metalloproteinases: A Role for MMP-13. Curr. Pharm. Biotechnol..

[4] Troeberg L., Nagase H. (2012). Proteases Involved in Cartilage Matrix Degradation in Osteoarthritis. Biochim. Biophys. Acta.

[5] Ågren M.S., Auf dem Keller U. (2020). Matrix Metalloproteinases: How Much Can They Do?. Int. J. Mol. Sci..

[6] Vandenbroucke R.E., Libert C. (2014). Is There New Hope for Therapeutic Matrix Metalloproteinase Inhibition?. Nat. Rev. Drug Discov..

[7] Cathcart J.M., Cao J. (2015). MMP Inhibitors: Past, Present and Future. Front. Biosci..

[8] Xie X.-W., Wan R.-Z., Liu Z.-P. (2017). Recent Research Advances in Selective Matrix Metalloproteinase-13 Inhibitors as Anti-Osteoarthritis Agents. ChemMedChem.

[9] Nuti E., Cuffaro D., Bernardini E., Camodeca C., Panelli L., Chaves S., Ciccone L., Tepshi L., Vera L., Orlandini E. (2018). Development of Thioaryl-Based Matrix Metalloproteinase-12 Inhibitors with Alternative Zinc-Binding Groups: Synthesis, Potentiometric, NMR, and Crystallographic Studies. J. Med. Chem..

[10] Nuti E., Rossello A., Cuffaro D., Camodeca C., Van Bael J., van der Maat D., Martens E., Fiten P., Pereira R.V.S., Ugarte-Berzal E. (2020). Bivalent Inhibitor with Selectivity for Trimeric MMP-9 Amplifies Neutrophil Chemotaxis and Enables Functional Studies on MMP-9 Proteoforms. Cells.

[11] Camodeca C., Cuffaro D., Nuti E., Rossello A. (2019). ADAM Metalloproteinases as Potential Drug Targets. Curr. Med. Chem..

[12] Pece R., Tavella S., Costa D., Varesano S., Camodeca C., Cuffaro D., Nuti E., Rossello A., Alfano M., D’Arrigo C. (2022). Inhibitors of ADAM10 Reduce Hodgkin Lymphoma Cell Growth in 3D Microenvironments and Enhance Brentuximab-Vedotin Effect. Haematologica.

[13] Cuffaro D., Ciccone L., Rossello A., Nuti E., Santamaria S. (2022). Targeting Aggrecanases for Osteoarthritis Therapy: From Zinc Chelation to Exosite Inhibition. J. Med. Chem..

[14] Supuran C.T. (2021). Novel Carbonic Anhydrase Inhibitors. Future Med. Chem..

[15] Cuffaro D., Nuti E., Rossello A. (2020). An Overview of Carbohydrate-Based Carbonic Anhydrase Inhibitors. J. Enzyme Inhib. Med. Chem..

[16] Li G., Tian Y., Zhu W.-G. (2020). The Roles of Histone Deacetylases and Their Inhibitors in Cancer Therapy. Front. Cell Dev. Biol..

[17] Nencetti S., Cuffaro D., Nuti E., Ciccone L., Rossello A., Fabbi M., Ballante F., Ortore G., Carbotti G., Campelli F. (2021). Identification of Histone Deacetylase Inhibitors with (Arylidene)Aminoxy Scaffold Active in Uveal Melanoma Cell Lines. J. Enzyme Inhib. Med. Chem..

[18] Fabre B., Ramos A., de Pascual-Teresa B. (2014). Targeting Matrix Metalloproteinases: Exploring the Dynamics of the S1′ Pocket in the Design of Selective, Small Molecule Inhibitors. J. Med. Chem..

[19] Johnson A.R., Pavlovsky A.G., Ortwine D.F., Prior F., Man C.-F., Bornemeier D.A., Banotai C.A., Mueller W.T., McConnell P., Yan C. (2007). Discovery and Characterization of a Novel Inhibitor of Matrix Metalloprotease-13 That Reduces Cartilage Damage in Vivo without Joint Fibroplasia Side Effects. J. Biol. Chem..

[20] Schnute M.E., O’Brien P.M., Nahra J., Morris M., Howard Roark W., Hanau C.E., Ruminski P.G., Scholten J.A., Fletcher T.R., Hamper B.C. (2010). Discovery of (Pyridin-4-Yl)-2H-Tetrazole as a Novel Scaffold to Identify Highly Selective Matrix Metalloproteinase-13 Inhibitors for the Treatment of Osteoarthritis. Bioorg. Med. Chem. Lett..

[21] Nara H., Sato K., Naito T., Mototani H., Oki H., Yamamoto Y., Kuno H., Santou T., Kanzaki N., Terauchi J. (2014). Discovery of Novel, Highly Potent, and Selective Quinazoline-2-Carboxamide-Based Matrix Metalloproteinase (MMP)-13 Inhibitors without a Zinc Binding Group Using a Structure-Based Design Approach. J. Med. Chem..

[22] Taylor S.J., Abeywardane A., Liang S., Muegge I., Padyana A.K., Xiong Z., Hill-Drzewi M., Farmer B., Li X., Collins B. (2011). Fragment-Based Discovery of Indole Inhibitors of Matrix Metalloproteinase-13. J. Med. Chem..

[23] Specs. http://www.specs.net.

[24] Nara H., Sato K., Naito T., Mototani H., Oki H., Yamamoto Y., Kuno H., Santou T., Kanzaki N., Terauchi J. (2014). Thieno[2,3-d]Pyrimidine-2-Carboxamides Bearing a Carboxybenzene Group at 5-Position: Highly Potent, Selective, and Orally Available MMP-13 Inhibitors Interacting with the S1″ Binding Site. Bioorg. Med. Chem..

[25] Reaxys. https://www.reaxys.com/.

[26] Rossello A., Nuti E., Orlandini E., Carelli P., Rapposelli S., Macchia M., Minutolo F., Carbonaro L., Albini A., Benelli R. (2004). New N-Arylsulfonyl-N-Alkoxyaminoacetohydroxamic Acids as Selective Inhibitors of Gelatinase A (MMP-2). Bioorg. Med. Chem..

[27] McGovern S.L., Helfand B.T., Feng B., Shoichet B.K. (2003). A Specific Mechanism of Nonspecific Inhibition. J. Med. Chem..

[28] Flare^TM^, v6.1.0.

[29] Thota S., Rodrigues D.A., de Sena murteira Pinheiro P., Lima L.M., Fraga C.A.M., Barreiro E.J. (2018). N-Acylhydrazones as Drugs. Bioorg. Med. Chem. Lett..

[30] Socea L.-I., Barbuceanu S.-F., Pahontu E.M., Dumitru A.-C., Nitulescu G.M., Sfetea R.C., Apostol T.-V. (2022). Acylhydrazones and Their Biological Activity: A Review. Molecules.

[31] Fortuna C.G., Bonaccorso C., Bulbarelli A., Caltabiano G., Rizzi L., Goracci L., Musumarra G., Pace A., Palumbo Piccionello A., Guarcello A. (2013). New Linezolid-like 1,2,4-Oxadiazoles Active against Gram-Positive Multiresistant Pathogens. Eur. J. Med. Chem..

[32] Palla G., Predieri G., Domiano P., Vignali C., Turner W. (1986). Conformational Behaviour and E/Z Isomerization of N-Acyl and N-Aroylhydrazones. Tetrahedron.

[33] Syakaev V.V., Podyachev S.N., Buzykin B.I., Latypov S.K., Habicher W.D., Konovalov A.I. (2006). NMR Study of Conformation and Isomerization of Aryl- and Heteroarylaldehyde 4-Tert-Butylphenoxyacetylhydrazones. J. Mol. Struct..

[34] Wyrzykiewicz E., Błaszczyk A., Turowska-Tyrk I. (2000). N-(E)-2-Stilbenyloxymethylenecarbonyl Substituted Hydrazones of Ortho, Meta and Para Hydroxybenzaldehydes. Bull. Pol. Acad. Sci. Chem..

[35] Munir R., Javid N., Zia-Ur-Rehman M., Zaheer M., Huma R., Roohi A., Athar M.M. (2021). Synthesis of Novel N-Acylhydrazones and Their C-N/N-N Bond Conformational Characterization by NMR Spectroscopy. Molecules.

[36] Tian B., He M., Tang S., Hewlett I., Tan Z., Li J., Jin Y., Yang M. (2009). Synthesis and Antiviral Activities of Novel Acylhydrazone Derivatives Targeting HIV-1 Capsid Protein. Bioorg. Med. Chem. Lett..

[37] Kuodis Z., Rutavičius A., Matijoška A., Eicher-Lorka O. (2007). Synthesis and Isomerism of Hydrazones of 2-(5-Thioxo-4,5-Dihydro-1,3,4-Thiadiazol-2-Ylthio)Acetohydrazide. Cent. Eur. J. Chem..

[38] Litvinov I.A., Kataeva O.N., Ermolaeva L.V., Vagina G.A., Troepol’skaya T.V., Naumov V.A. (1991). Crystal and Molecular Structure of Aroyl- and Acetylhydrazones of Acet- and Benzaldehydes. Russ. Chem. Bull..

[39] Pitasse-Santos P., Sueth-Santiago V., Lima M.E.F. (2018). 1,2,4- and 1,3,4-Oxadiazoles as Scaffolds in the Development of Antiparasitic Agents. J. Braz. Chem. Soc..

[40] Bredael K., Geurs S., Clarisse D., De Bosscher K., D’hooghe M. (2022). Carboxylic Acid Bioisosteres in Medicinal Chemistry: Synthesis and Properties. J. Chem..

[41] Cuffaro D., Camodeca C., D’Andrea F., Piragine E., Testai L., Calderone V., Orlandini E., Nuti E., Rossello A. (2018). Matrix Metalloproteinase-12 Inhibitors: Synthesis, Structure-Activity Relationships and Intestinal Absorption of Novel Sugar-Based Biphenylsulfonamide Carboxylates. Bioorg. Med. Chem..

[42] Cuffaro D., Nuti E., Gifford V., Ito N., Camodeca C., Tuccinardi T., Nencetti S., Orlandini E., Itoh Y., Rossello A. (2019). Design, Synthesis and Biological Evaluation of Bifunctional Inhibitors of Membrane Type 1 Matrix Metalloproteinase (MT1-MMP). Bioorg. Med. Chem..

[43] Hawkins P.C.D., Skillman A.G., Warren G.L., Ellingson B.A., Stahl M.T. (2010). Conformer Generation with OMEGA: Algorithm and Validation Using High Quality Structures from the Protein Databank and Cambridge Structural Database. J. Chem. Inf. Model..

[44] Hawkins P.C.D., Skillman A.G., Warren G.L., Ellingson B.A., Stahl M.T. (2018). OMEGA, v.3.0.1.2.

[45] Hawkins P.C.D., Skillman A.G., Nicholls A. (2007). Comparison of Shape-Matching and Docking as Virtual Screening Tools. J. Med. Chem..

[46] (2017). ROCS, Version 3.2.2.2.

[47] Shape Theory—Applications. https://docs.eyesopen.com/applications/rocs/theory/shape_shape.html.

[48] (2016). Schrödinger Release 2016-3: LigPrep.

[49] (2016). Schrödinger Release 2016-3: Epik.

[50] (2016). Schrödinger Release 2016-3: Schrödinger Suite 2016-3 Protein Preparation Wizard.

[51] (2016). Schrödinger Release 2016-3: Maestro.

[52] (2016). Schrödinger Release 2016-3: Glide.

