# Supplementary material for: Identification of N-Acyl Hydrazones as New Non-Zinc-Binding MMP-13 Inhibitors by Structure-Based Virtual Screening Studies and Chemical Optimization
Source: Int J Mol Sci. 2023 Jul 4;24(13):11098. doi: 10.3390/ijms241311098 (PMC10342305; doi:10.3390/ijms241311098)

SUPPORTING INFORMATION FOR

Identification of N-Acyl Hydrazones as New Non-Zinc-Binding MMP-13 Inhibitors by Structure-Based Virtual Screening Studies and Chemical Optimization

Content:

I. Combustion data analysis S2

II. Supplementary figures S3

III. References S10

IV. Representative NMR spectra S11

V. Representative HRMS spectra S25

Table S1. Combustion analysis data of final compounds 13, 13a-o.

Compound	Formula	Calculated			Found		
		% C	% H	% N	% C	% H	% N
**13**	$C_{21}H_{17}BrN_4O_5$	51.97	3.53	11.55	51.99	3.54	11.58
**13a**	$C_{21}H_{19}N_5O_7S$	51.96	3.95	14.43	51.98	3.99	14.45
**13b**	$C_{22}H_{20}N_4O_6$	60.55	4.62	12.84	60.58	4.64	12.87
**13c**	$C_{20}H_{15}BrN_4O_5$	50.97	3.21	11.89	51.00	3.23	11.92
**13d**	$C_{21}H_{18}N_4O_6$	59.71	4.30	13.26	59.72	4.34	13.28
**13e**	$C_{24}H_{20}BrN_5O_4$	55.19	3.86	13.41	55.21	3.87	13.44
**13f**	$C_{25}H_{23}N_5O_5$	63.42	4.90	14.79	63.45	4.92	14.80
**13g**	$C_{24}H_{21}N_5O_5$	62.74	4.61	15.24	62.75	4.64	15.26
**13h**	$C_{22}H_{18}BrN_3O_5$	54.56	3.75	8.68	51.57	3.77	8.70
**13i**	$C_{23}H_{21}N_3O_6$	63.44	4.86	9.65	63.45	4.89	9.67
**13l**	$C_{21}H_{16}BrN_3O_5$	53.63	3.43	8.94	53.65	3.47	8.95
**13m**	$C_{22}H_{18}BrN_3O_5$	54.56	3.75	8.68	54.59	3.77	8.69
**13n**	$C_{21}H_{16}BrN_7O_3$	51.03	3.26	19.84	51.05	3.29	19.86
**13o**	$C_{22}H_{18}BrN_7O_3$	51.98	3.57	19.29	52.01	3.58	19.31

II. Supplementary figures

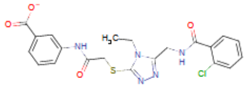

1 (AN-698/42116838)

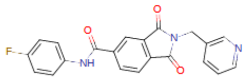

2 (AN-979/41971859)

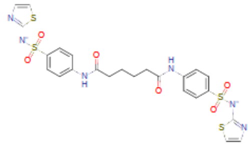

3 (AN-329/12244631)

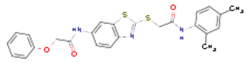

4 (AQ-088/42013452)

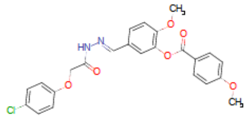

5 (AH-487/40686365)

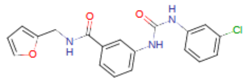

6 (AN-329/43448965)

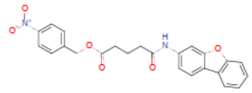

7 (AT-057/43445751)

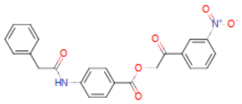

8 (AK-918/11884030)

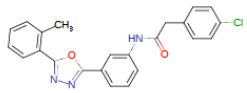

9 (AN-979/42723005)

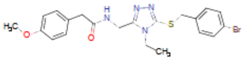

10 (AR-196/42726991)

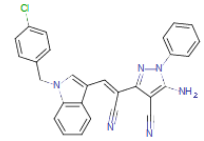

11 (AG-205/37007400)

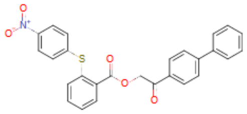

12 (AK-968/13281769)

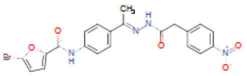

13 (AK-968/15605222)

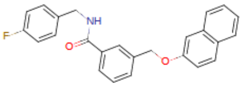

14 (AK-968/41024534)

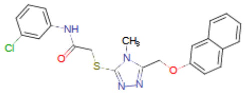

15 (AG-690/40752079)

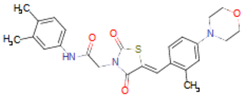

16 (AH-487/41658805)

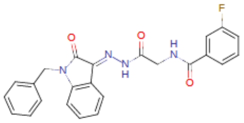

17 (AK-968/36929067)

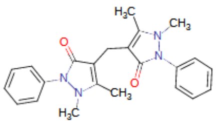

18 (AF-407/31882031)

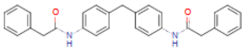

19 (AN-329/33523009)

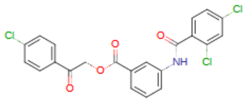

20 (AK-918/11884148)

Figure S1. 2D structures of the 20 compounds selected for *in vitro* test. Each compound is identified with its Specs ID number. MarvinSketch[1] was used to draw the structures. The protonation state of each compound corresponds to the protonation state of the docked pose selected for that compound.

A)

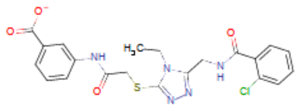

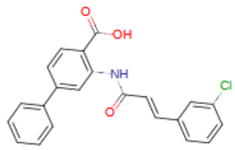

1 (AN-698/42116838) Reaxys RN: 12718029

Tanimoto: 0.39

B)

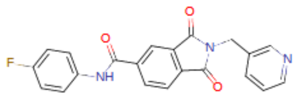

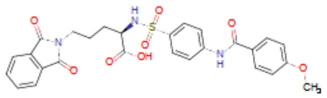

2 (AN-979/41971859) Reaxys RN: 25580841

Tanimoto: 0.45

C)

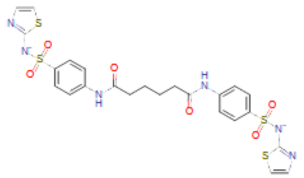

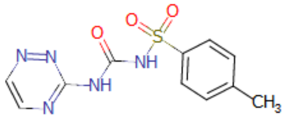

3 (AN-329/12244631) Reaxys RN: 26373808

Tanimoto: 0.32

D)

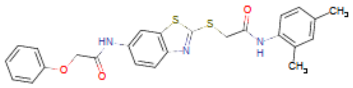

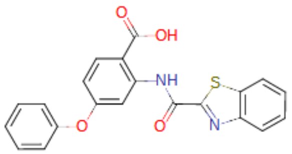

4 (AQ-088/42013452) Reaxys RN: 12718003

Tanimoto: 0.41

E)

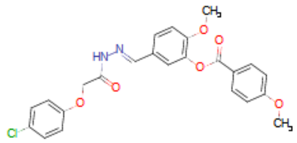

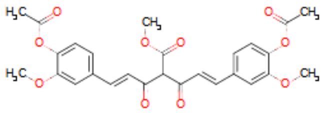

5 (AH-487/40686365) Reaxys RN: 20888062

Tanimoto: 0.42

F)

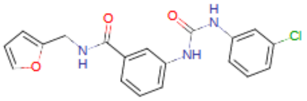

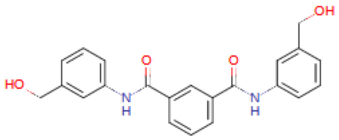

6 (AN-329/43448965) Reaxys RN: 13925759

Tanimoto: 0.49

G)

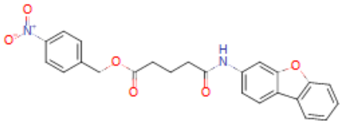

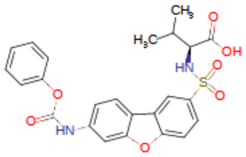

7 (AT-057/43445751) Reaxys RN: 25593517

Tanimoto: 0.42

H)

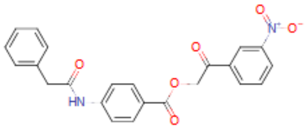

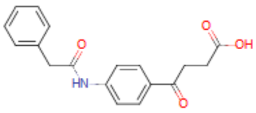

8 (AK-918/11884030) Reaxys RN: 13914187

Tanimoto: 0.54

I)

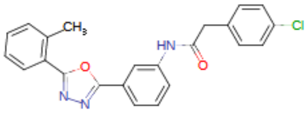

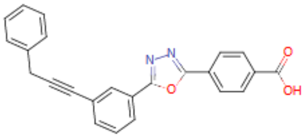

9 (AN-979/42723005) Reaxys RN: 11809753

Tanimoto: 0.49

J)

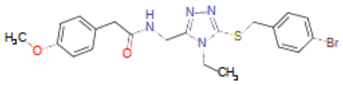

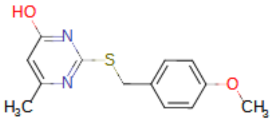

10 (AR-196/42726991) Reaxys RN: 799987

Tanimoto: 0.31

K)

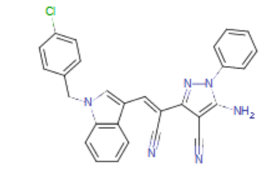

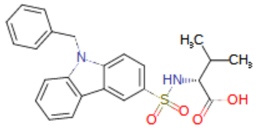

11 (AG-205/37007400) Reaxys RN: 26364583

Tanimoto: 0.43

L)

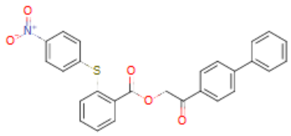

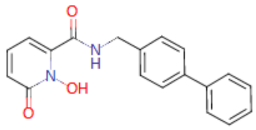

12 (AK-968/13281769) Reaxys RN: 19675912

Tanimoto: 0.38

M)

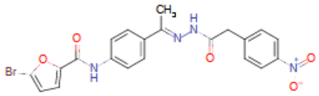

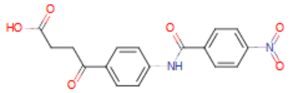

13 (AK-968/15605222) Reaxys RN: 13917890

Tanimoto: 0.43

N)

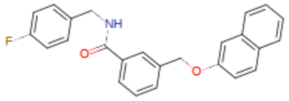

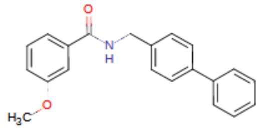

14 (AK-968/41024534) Reaxys RN: 24511368

Tanimoto: 0.71

O)

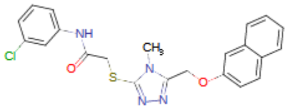

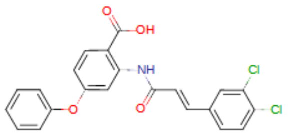

15 (AG-690/40752079) Reaxys RN: 12718197

Tanimoto: 0.47

P)

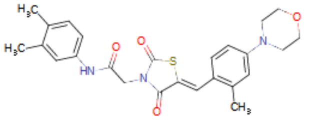

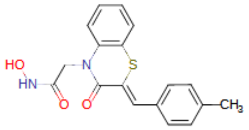

16 (AH-487/41658805) Reaxys RN: 11408221

Tanimoto: 0.39

Q)

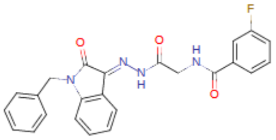

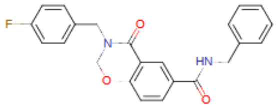

17 (AK-968/36929067) Reaxys RN: 12071045

Tanimoto: 0.44

R)

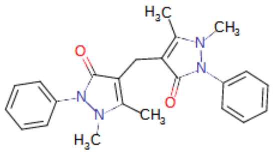

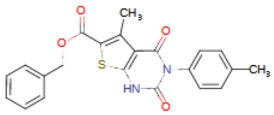

18 (AF-407/31882031) Reaxys RN: 24317044

Tanimoto: 0.25

S)

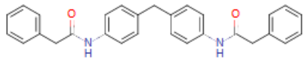

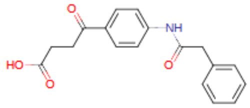

19 (AN-329/33523009) Reaxys RN: 13914187

Tanimoto: 0.47

T)

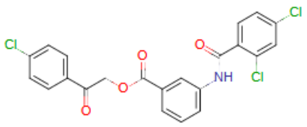

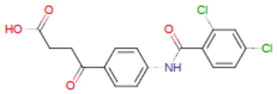

20 (AK-918/11884148) Reaxys RN: 13919256

Tanimoto: 0.60

Figure S2. In each panel, a hit compound is represented in 2D, together with the most similar active compound found in the Reaxys[2] database, which is labeled with its Reaxys Registry Number. The Tanimoto similarity value resulting from the comparison of the OpenEyePath[3] fingerprint of both compounds is shown below. MarvinSketch[1] was used to draw the structures.

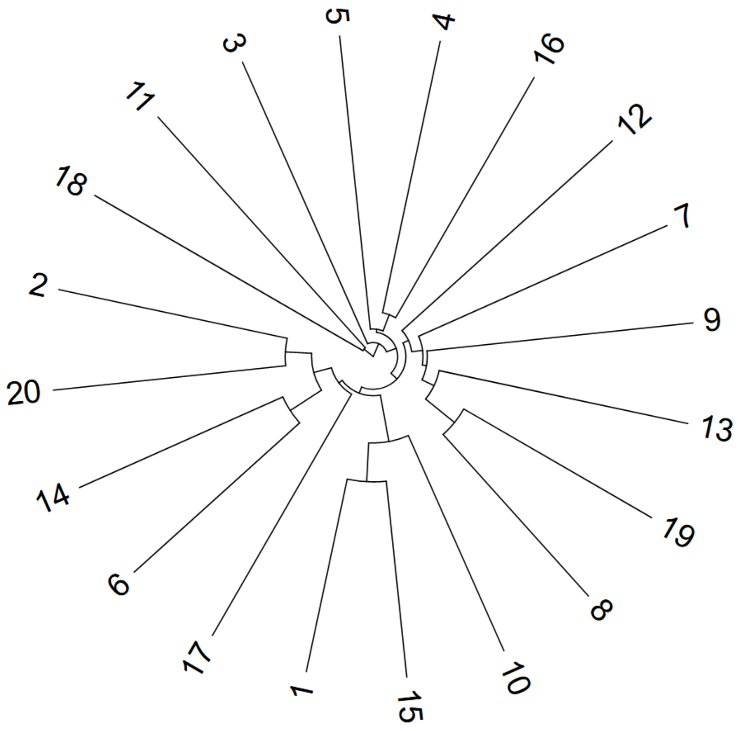

Figure S3. Dendogram based on fingerprints showing the structural diversity of the 20 selected compounds. The fingerprint used to obtain the distance matrix was the OpenEyePath fingerprint.[3] iTOL[4] was used to draw the dendogram.

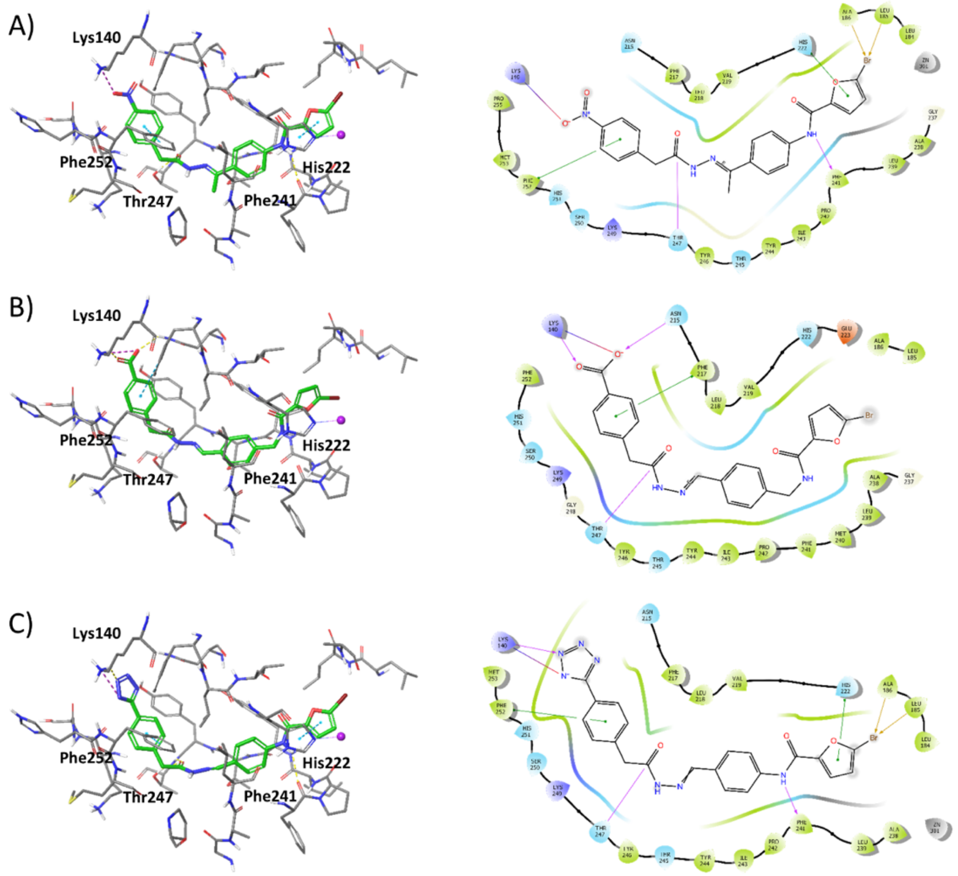

Figure S4. Protein-ligand interactions performed by the docked poses of compounds 13, 13m and 13n (panels A, B and C, respectively) with MMP-13. The first picture in each panel shows the intermolecular interactions predicted by docking in 3D (hydrogen bonds are colored in yellow, π stacking interactions are colored in cyan, and salt bridges are colored in magenta). The second picture shows the intermolecular interactions predicted by docking in 2D (hydrogen bonds, halogen bonds, π stacking interactions and salt bridges are represented by magenta arrows, yellow arrows, green lines and red and blue lines, respecively; positively charged, negatively charged, polar and hydrophobic residues are colored in blue, red, cyan and green, respectively). Figure obtained with Maestro (Schrödinger Release 2016-3: Maestro, Schrödinger, LLC, New York, NY, 2016).

III. References

[1] Marvin 16.10.10.0, 2016, ChemAxon http://www.chemaxon.com.

[2] Reaxys, https://www.reaxys.com/

[3] OpenEye scientific software, OEChem, 2013.

[4] Letunic, I.; Bork, P. Interactive Tree of Life (ITOL) v3: An Online Tool for the Display and Annotation of Phylogenetic and Other Trees. Nucleic Acids Res. 2016, 44 (W1), W242-5.

IV. Representative NMR spectra

^{1}H 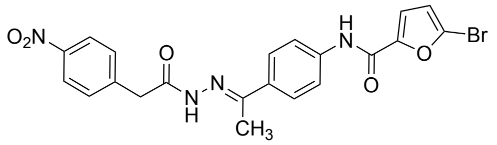 NMR of compound **13** (400MHz, DMSO-d_6)

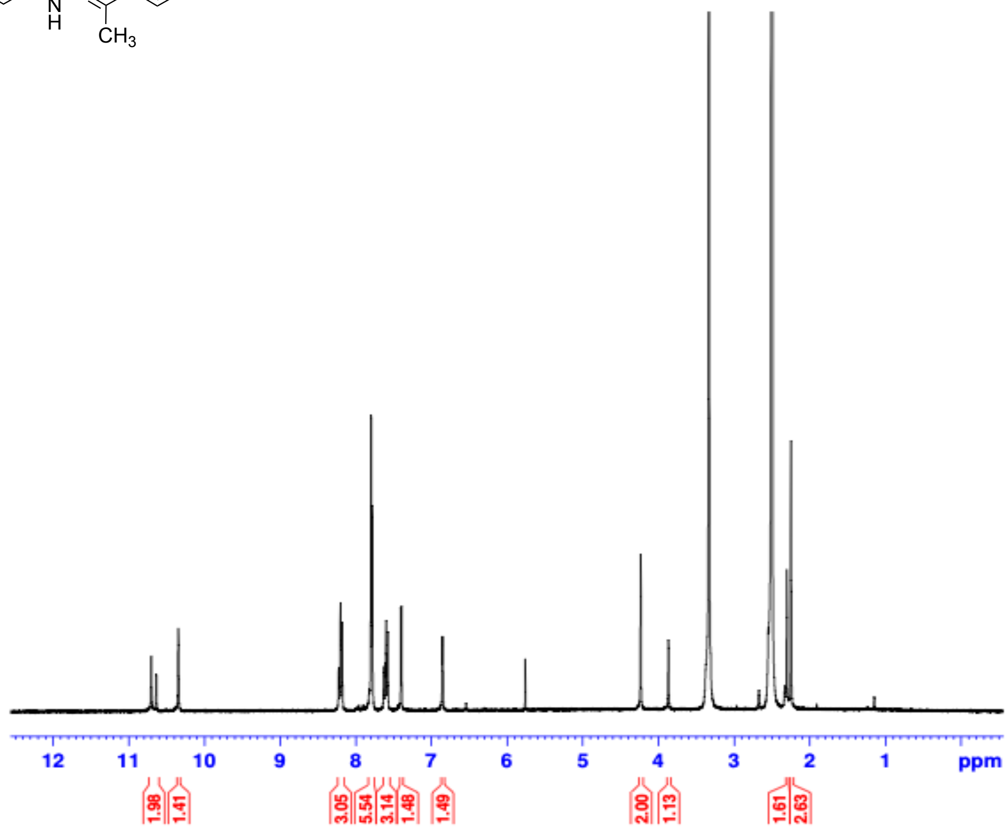

^{13}C NMR of compound **13** (100MHz, DMSO-d_6)

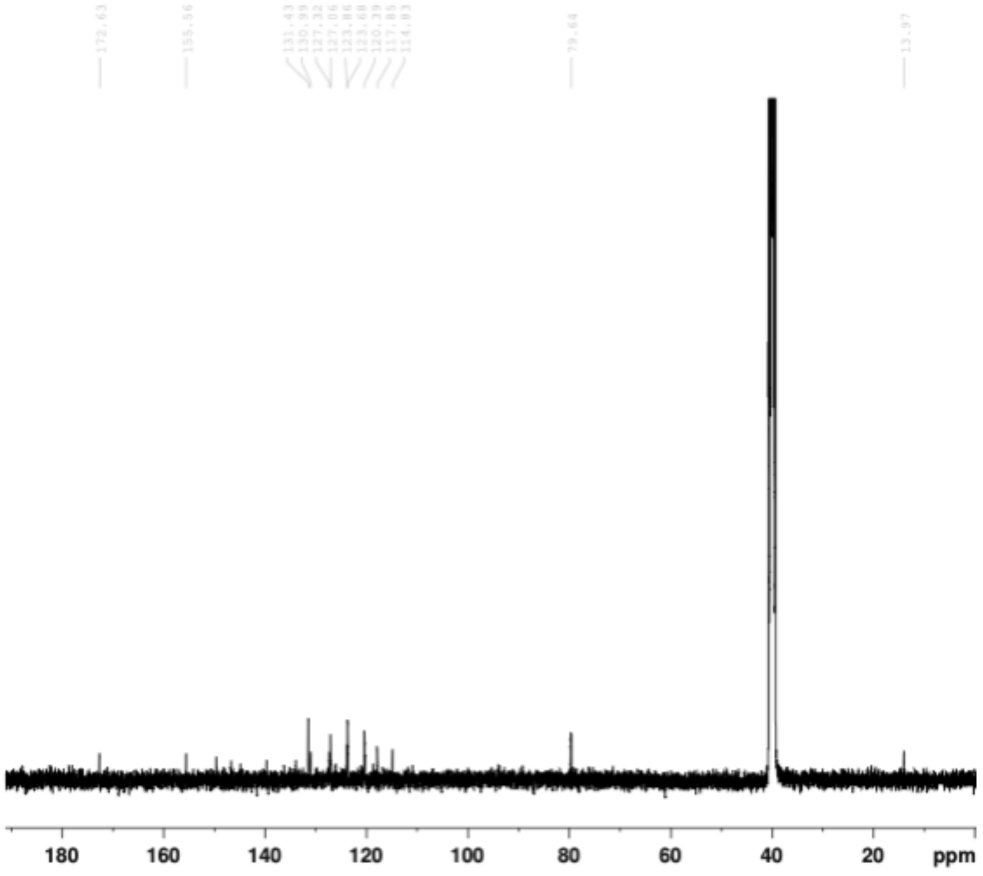

^{1}H NMR of compound **13a** (400MHz, DMSO-*d6*)

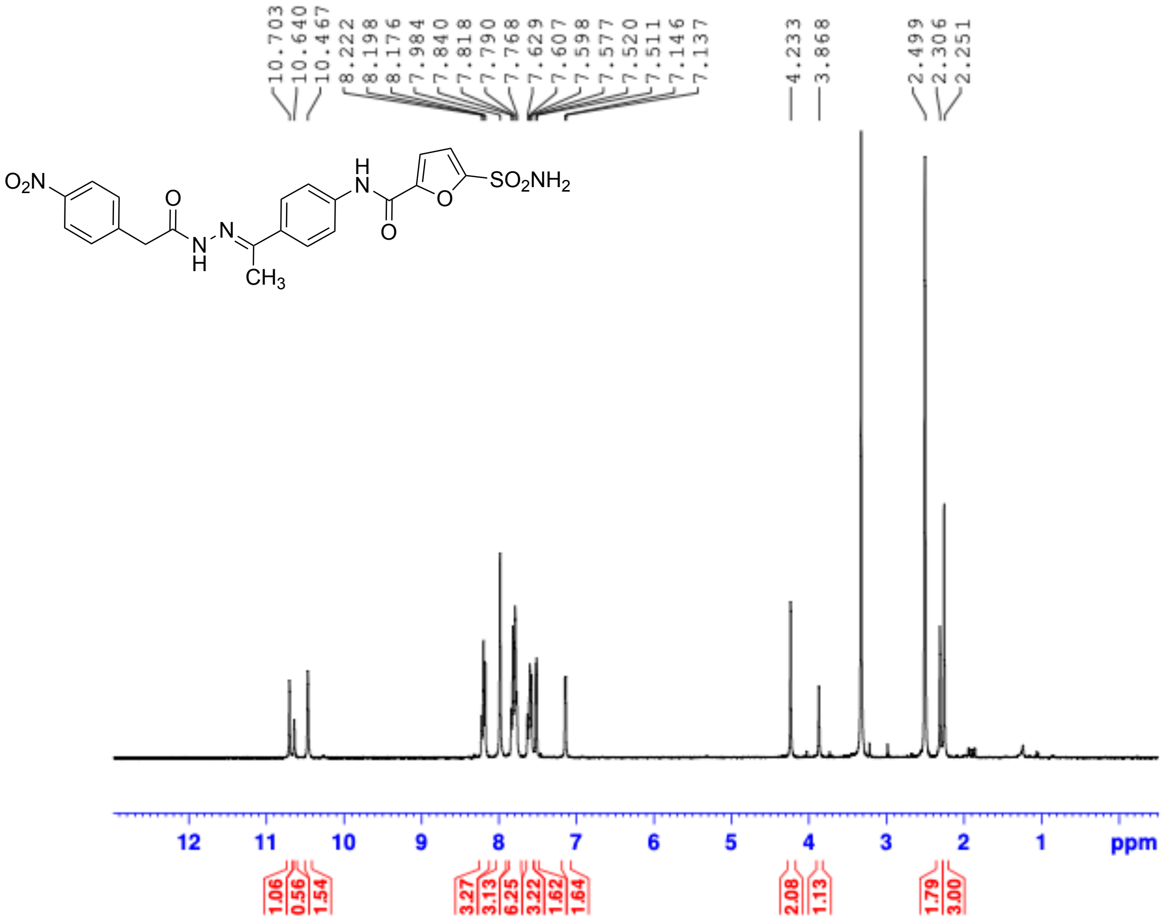

[13] C NMR of compound **13a** (100MHz, DMSO-d_6)

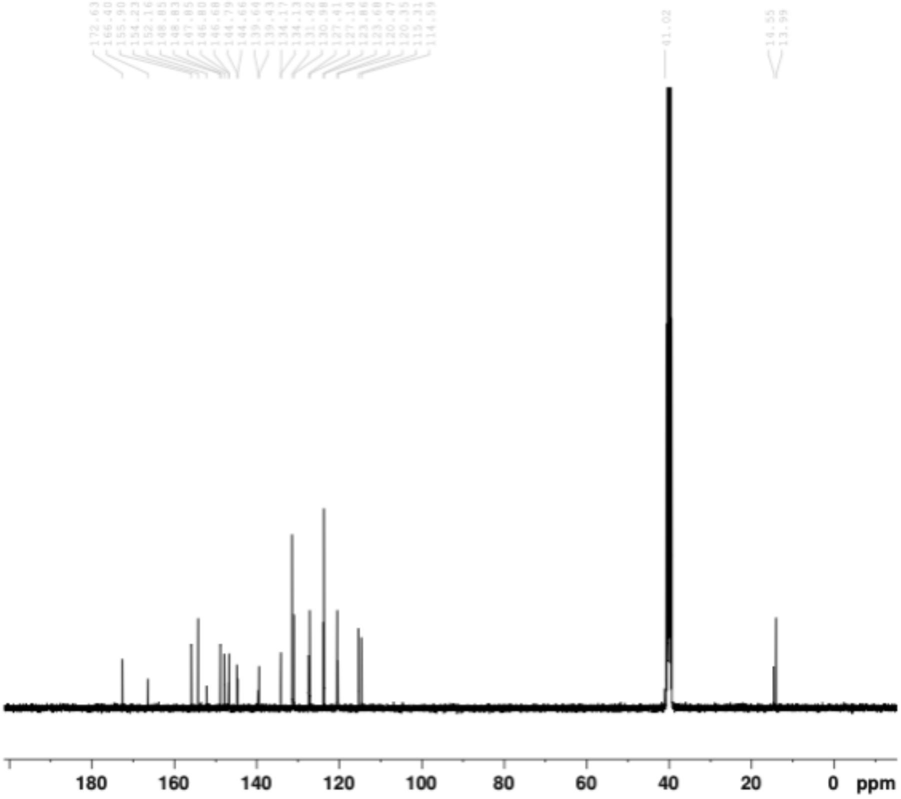

^{1}H NMR of compound **13b** (400MHz, DMSO-d_6)

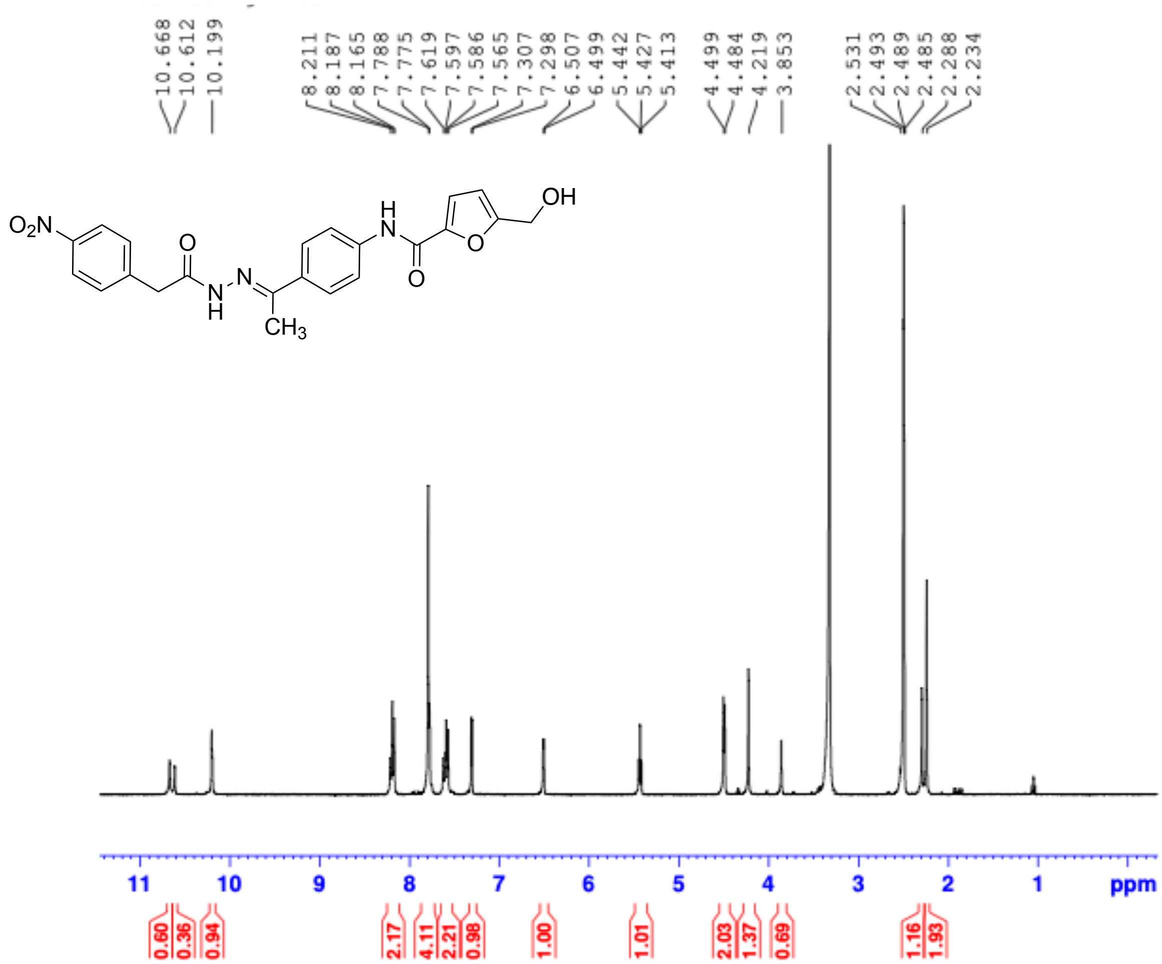

[13] C NMR of compound **13b** (100MHz, DMSO-d_6)

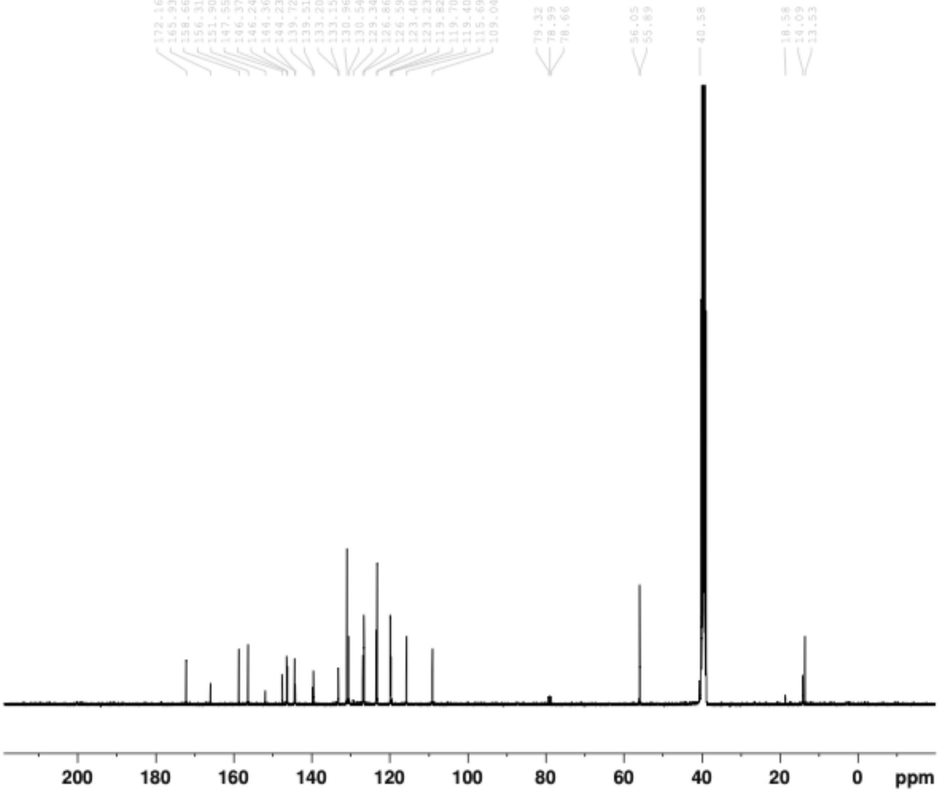

^{1}H NMR of compound **13c** (400MHz, DMSO-d_6)

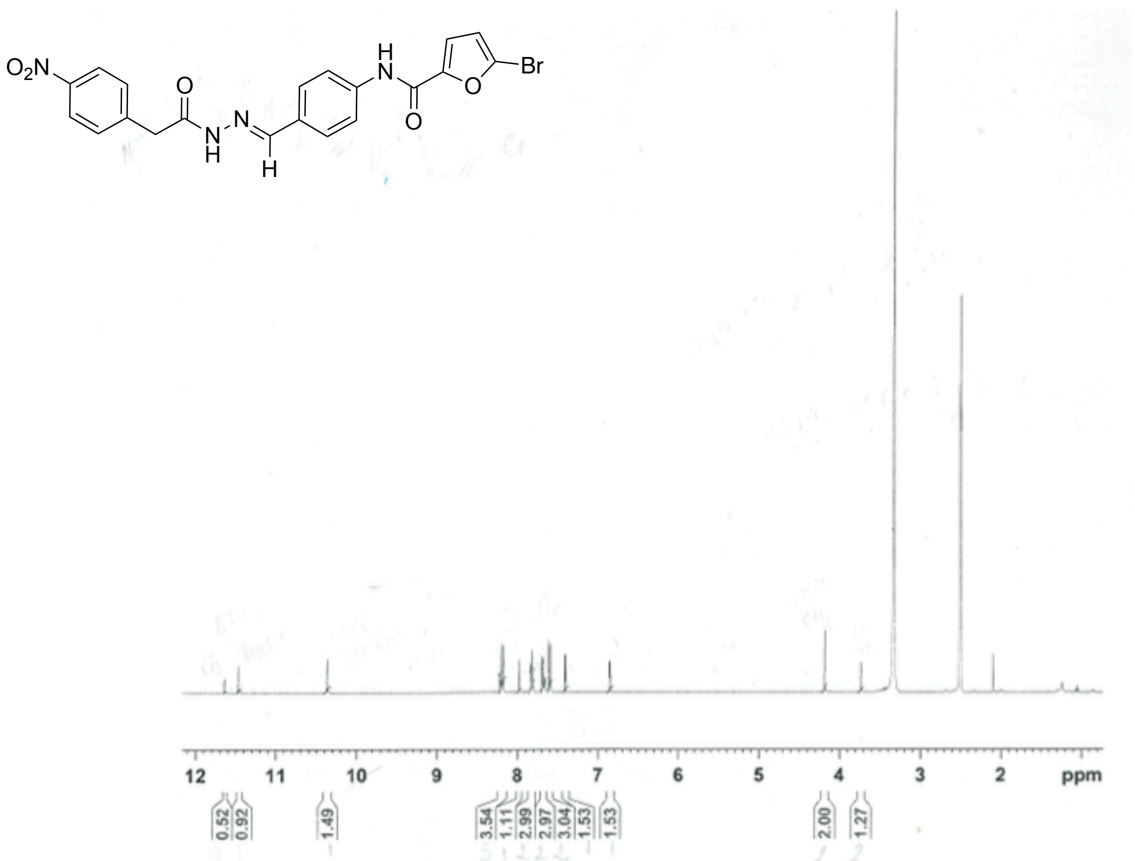

^{13}C NMR of compound **13c** (100MHz, DMSO-d_6)

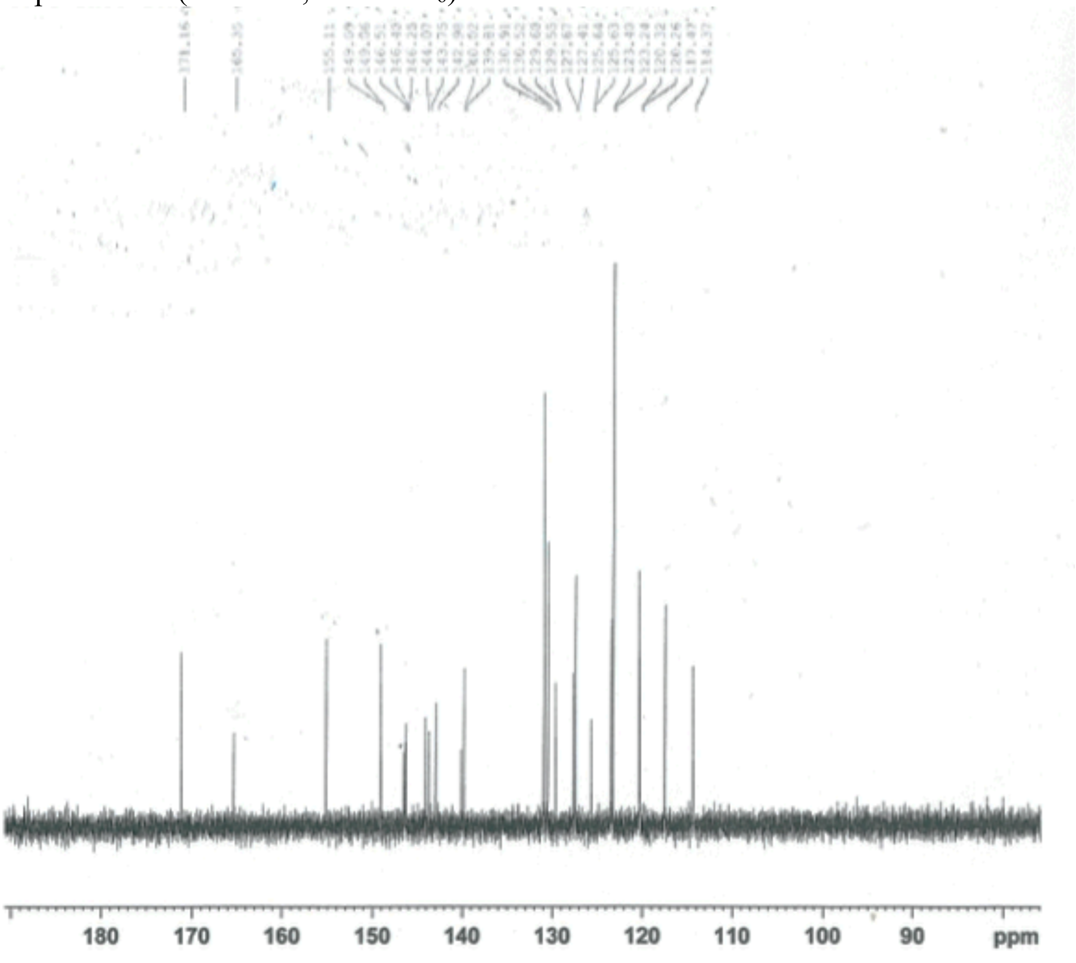

^{1}H NMR of compound **13d** (400MHz, DMSO-d_6)

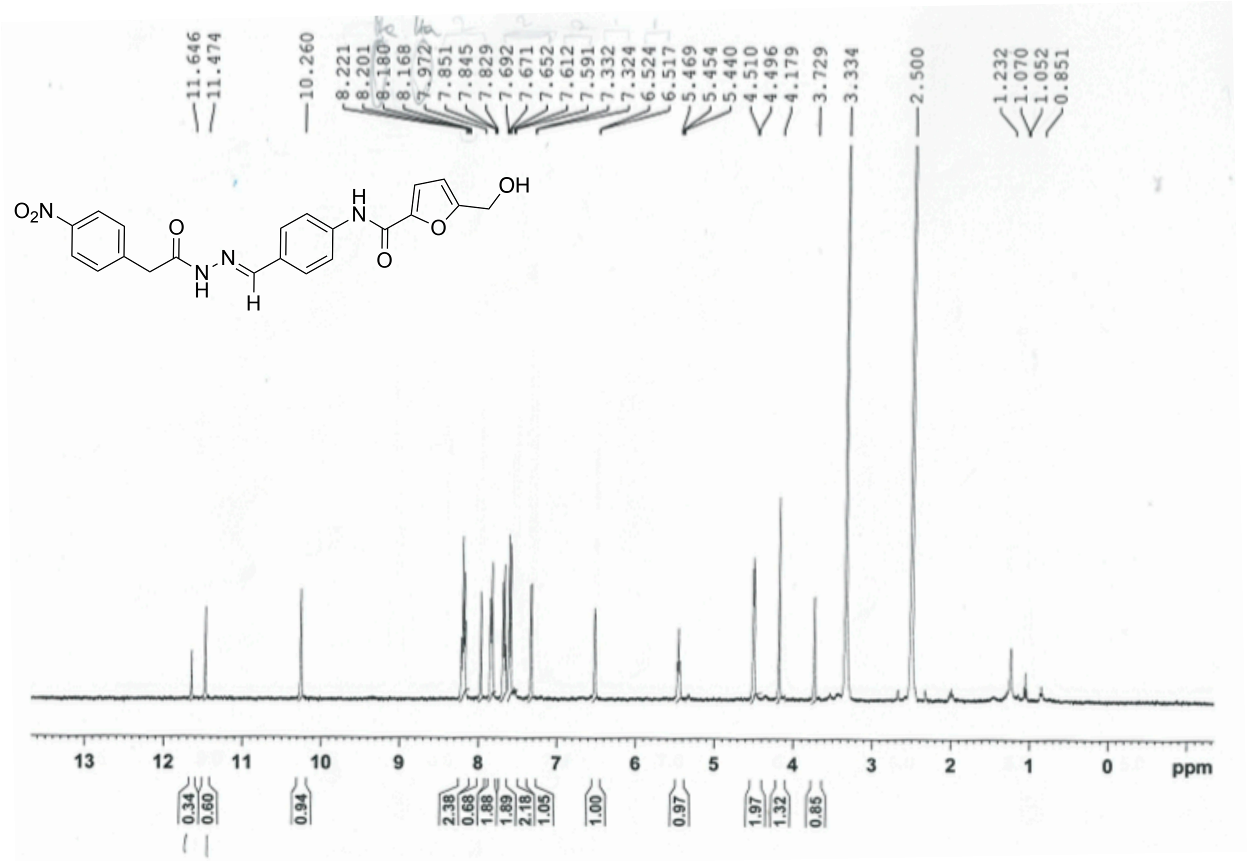

^{13}C NMR of compound **13d** (100MHz, DMSO-d_6)

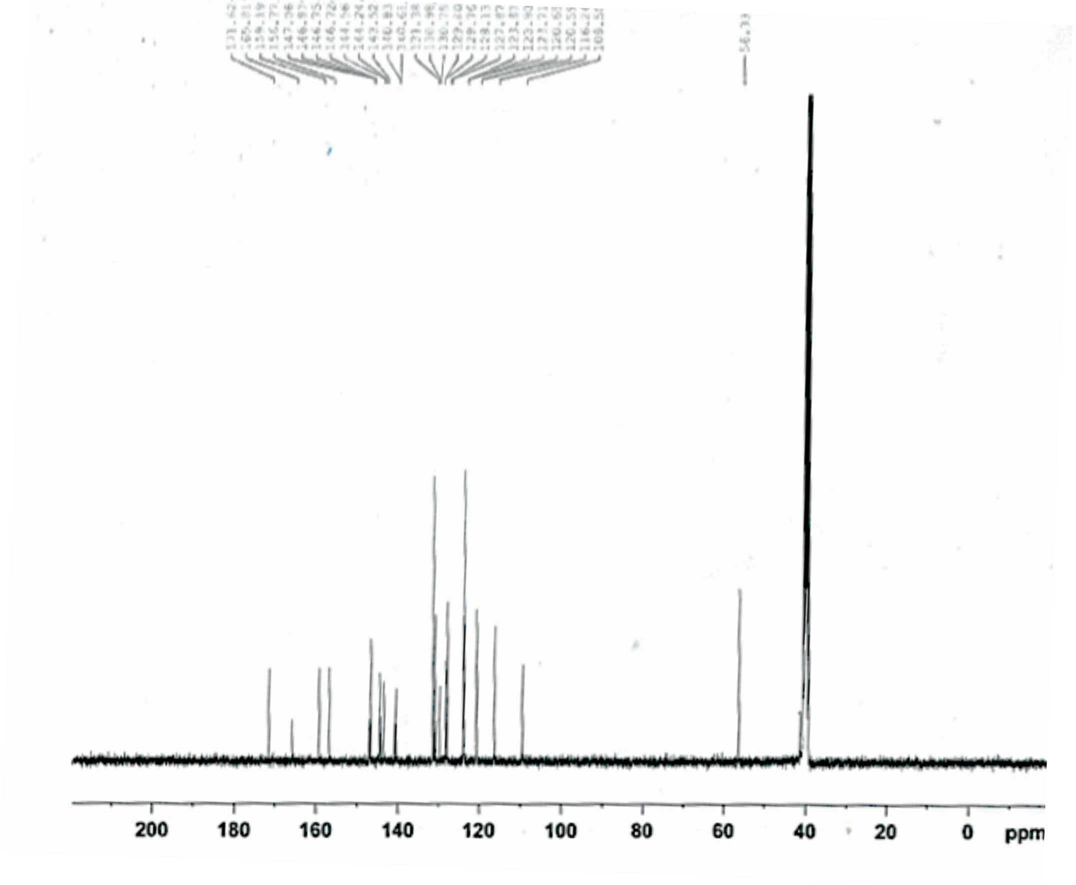

^{1}H NMR of compound **13e** (400MHz, DMSO-d_6)

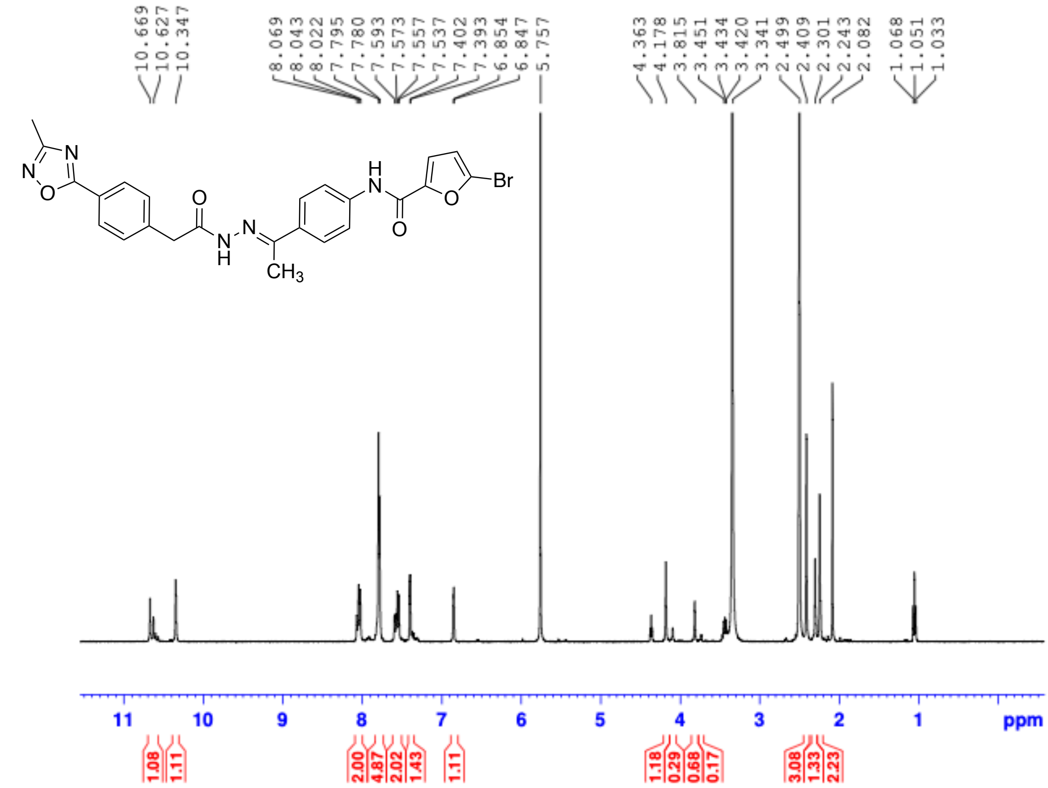

^{13}C NMR of compound **13e** (100MHz, DMSO-d_6)

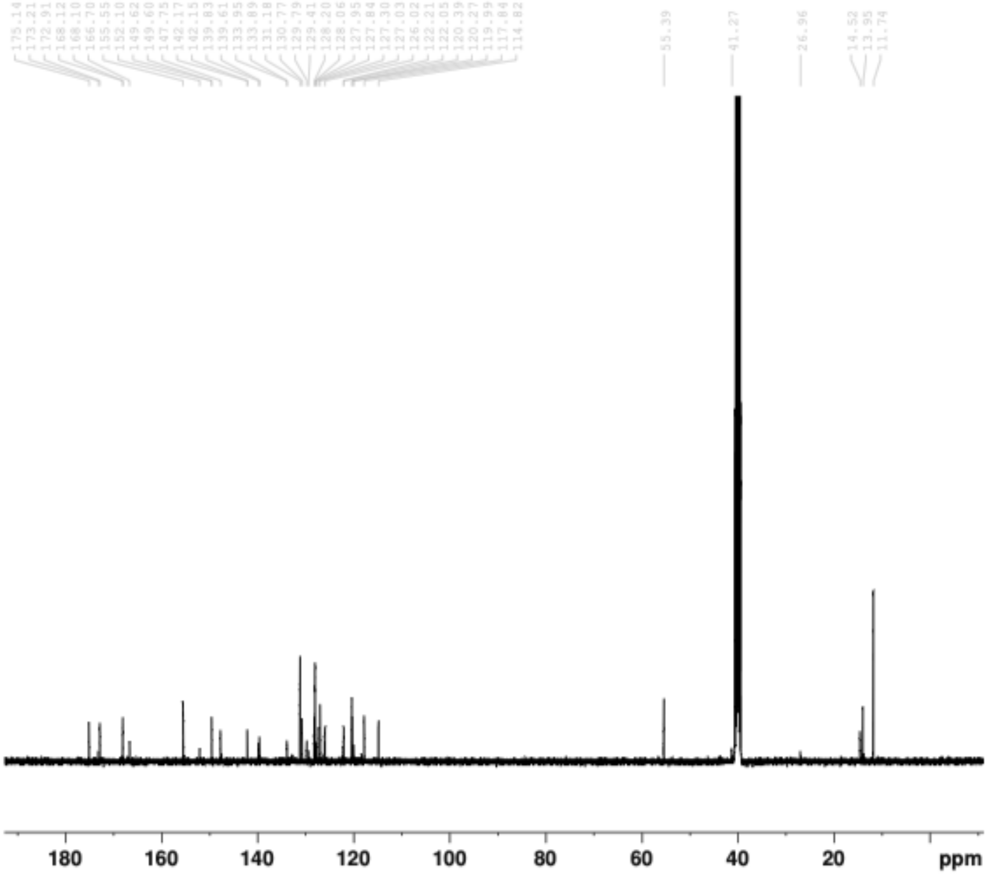

^{1}H NMR of compound **13f** (400MHz, DMSO-d_6)

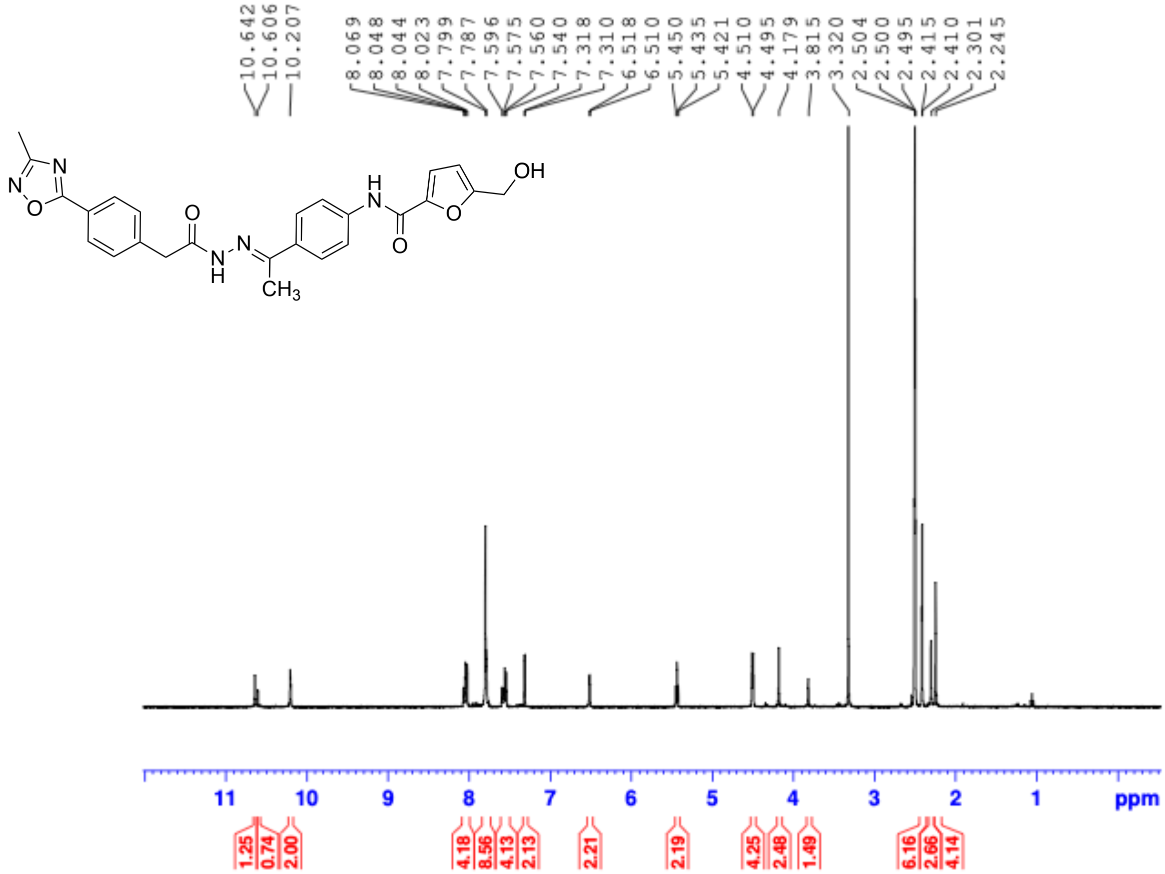

[13] C NMR of compound **13f** (100MHz, DMSO-d_6)

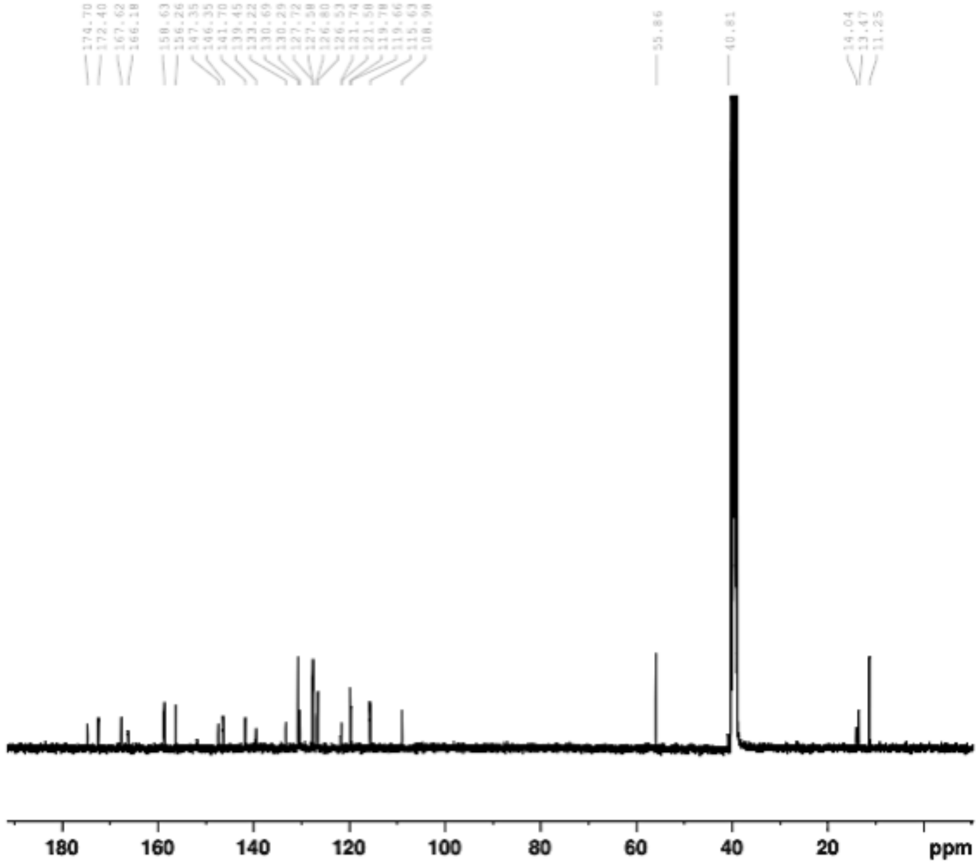

^{1}H NMR of compound **13g** (400MHz, DMSO-d_6)

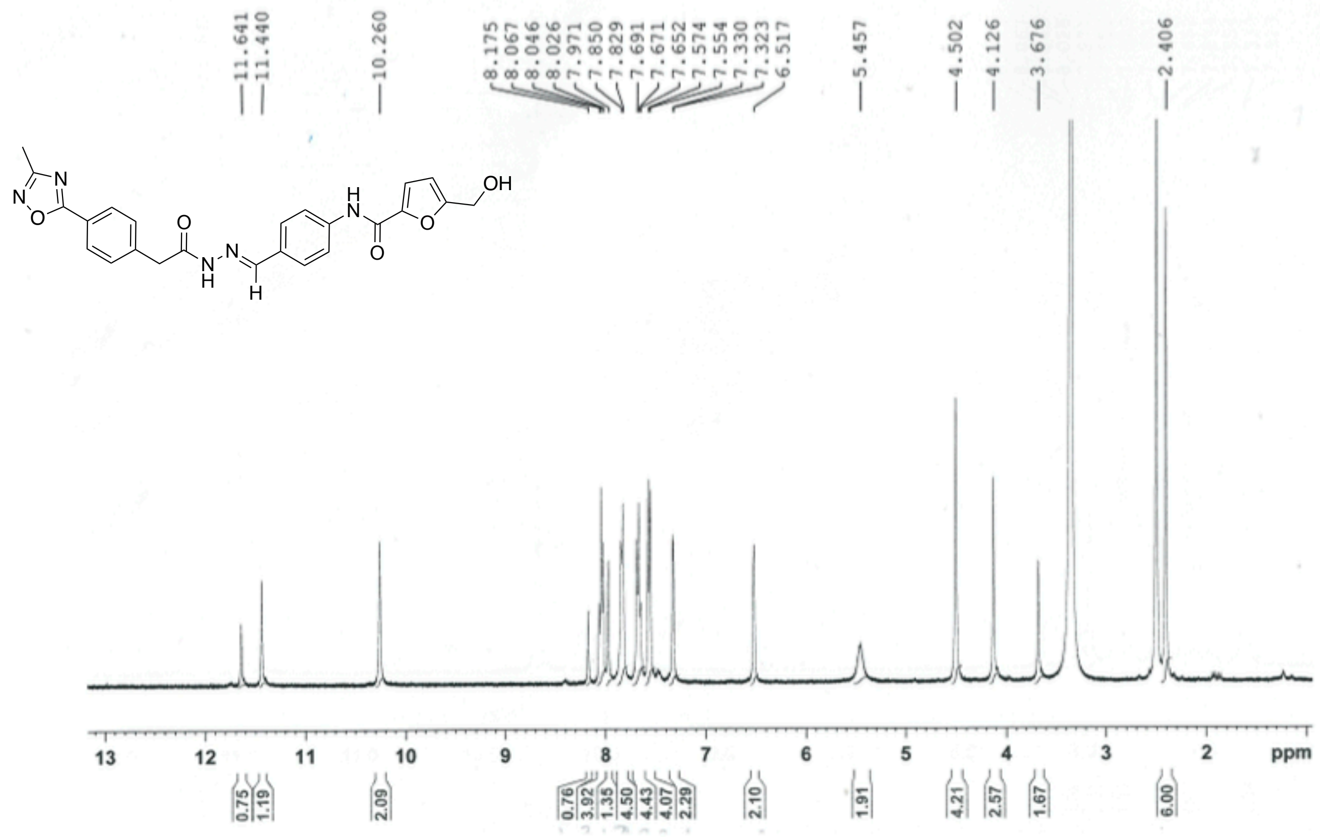

^{13}C NMR of compound **13g** (100MHz, DMSO-d_6)

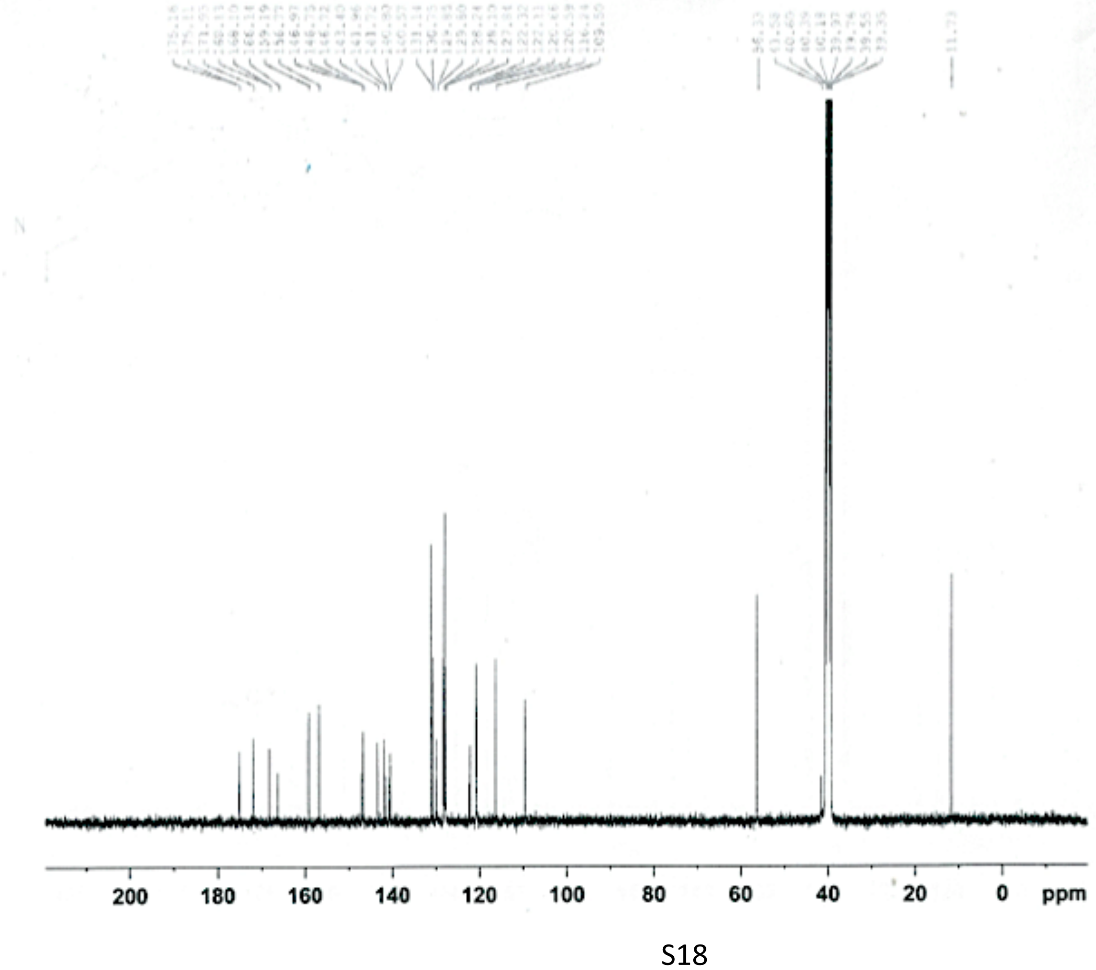

^{1}H NMR of compound **13h** (400MHz, DMSO-d_6)

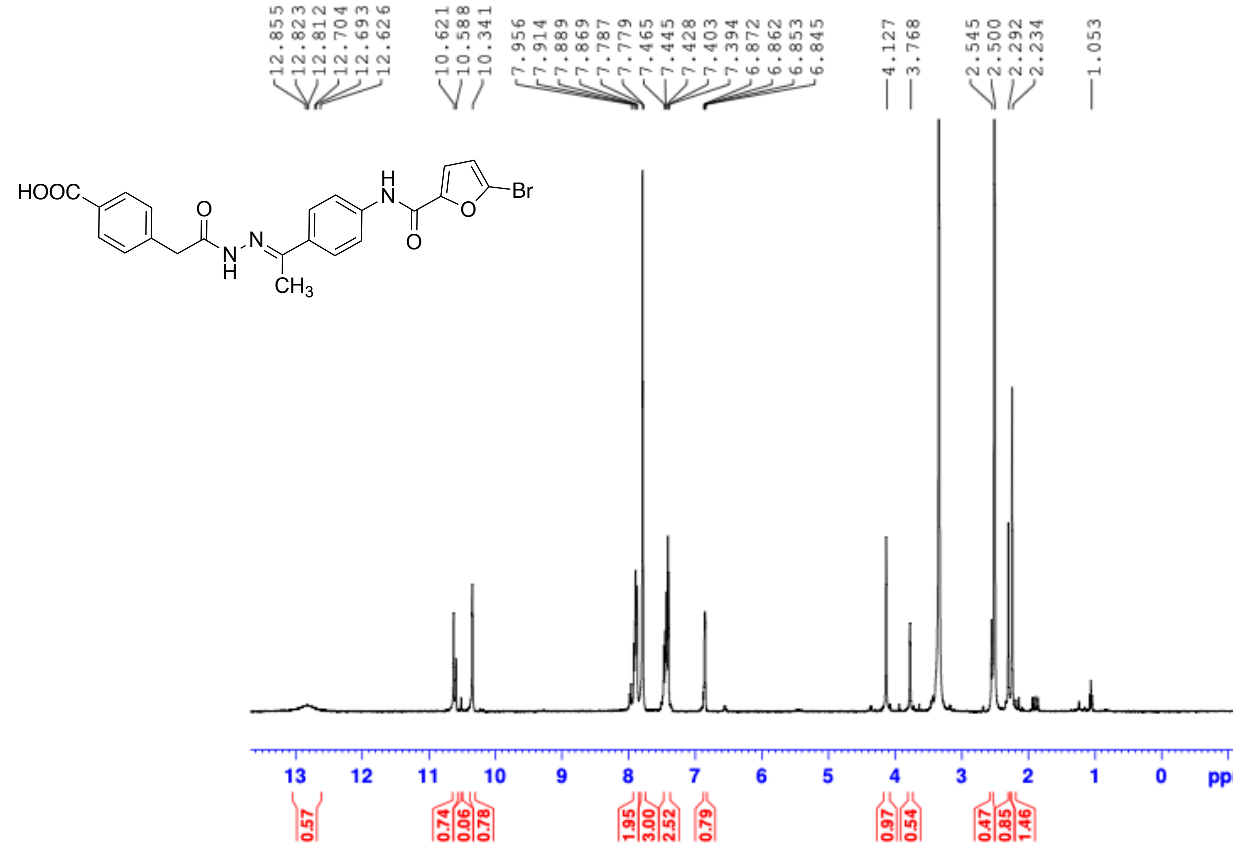

[13] C NMR of compound **13h** (100MHz, DMSO-d_6)

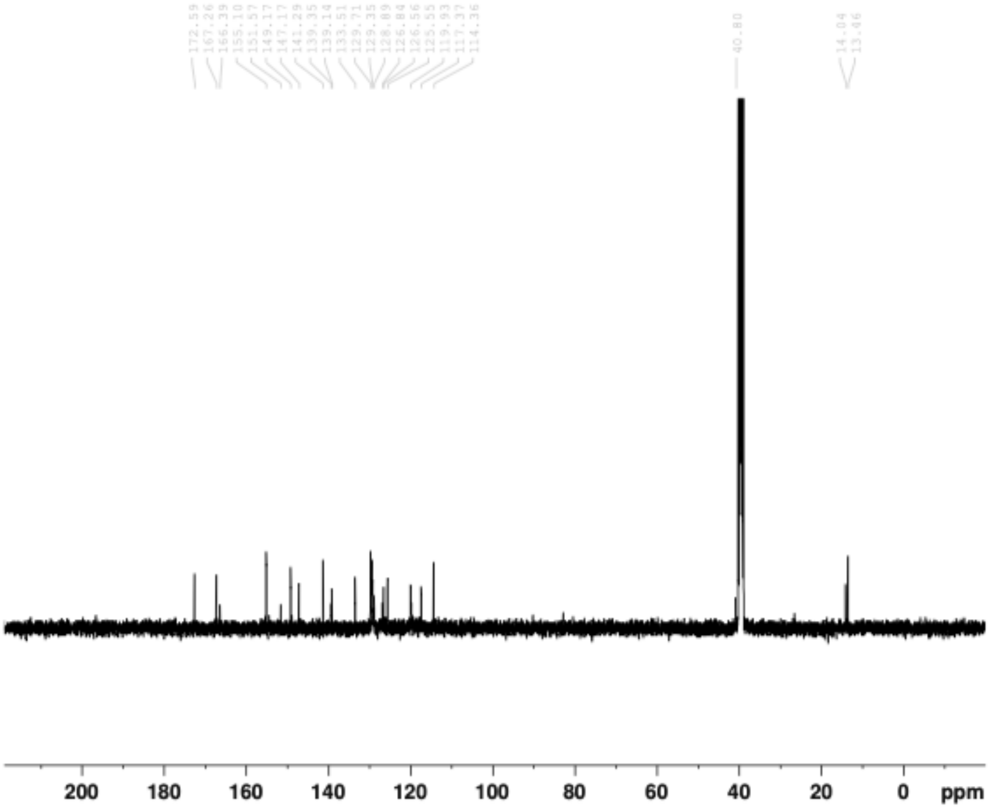

^{1}H NMR of compound **13i** (400MHz, DMSO-d_6)

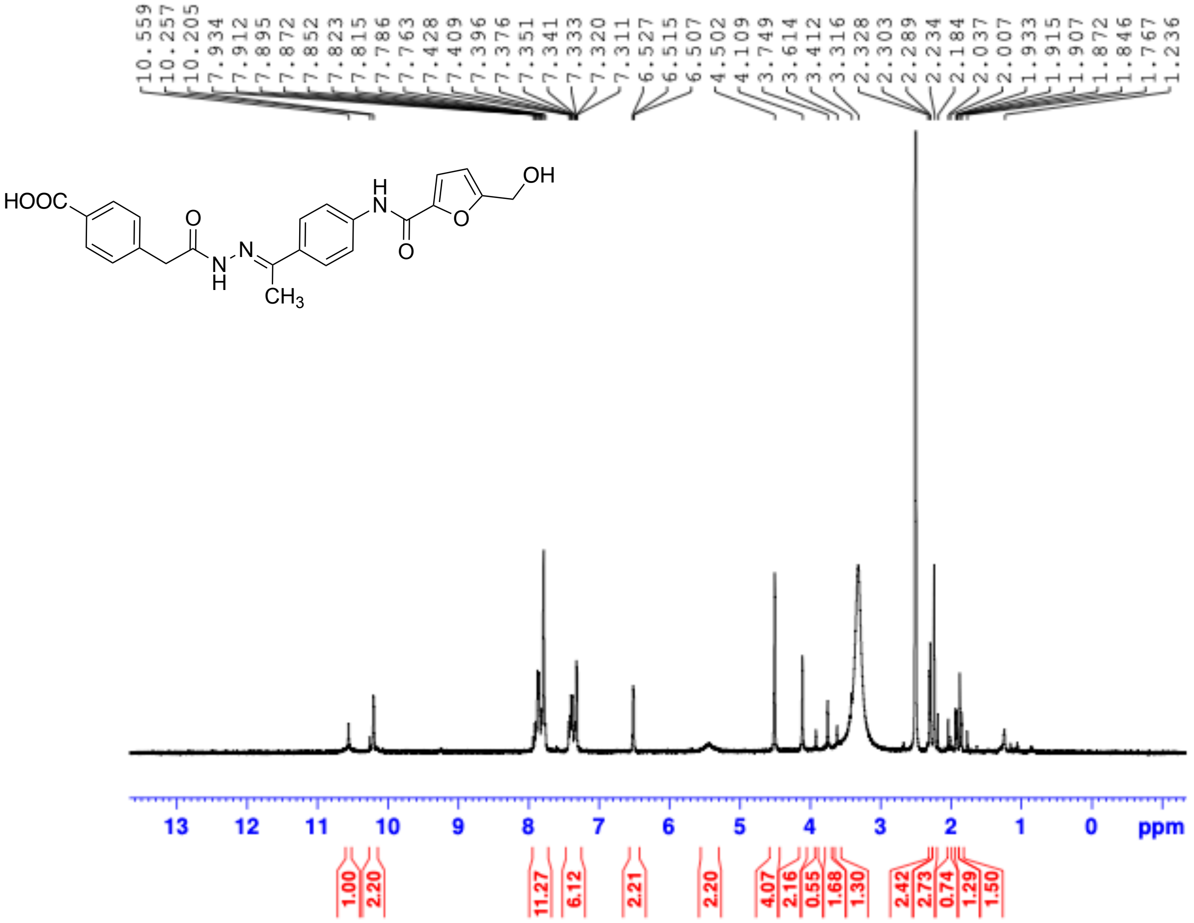

^{13}C NMR of compound **13i** (100MHz, DMSO-d_6)

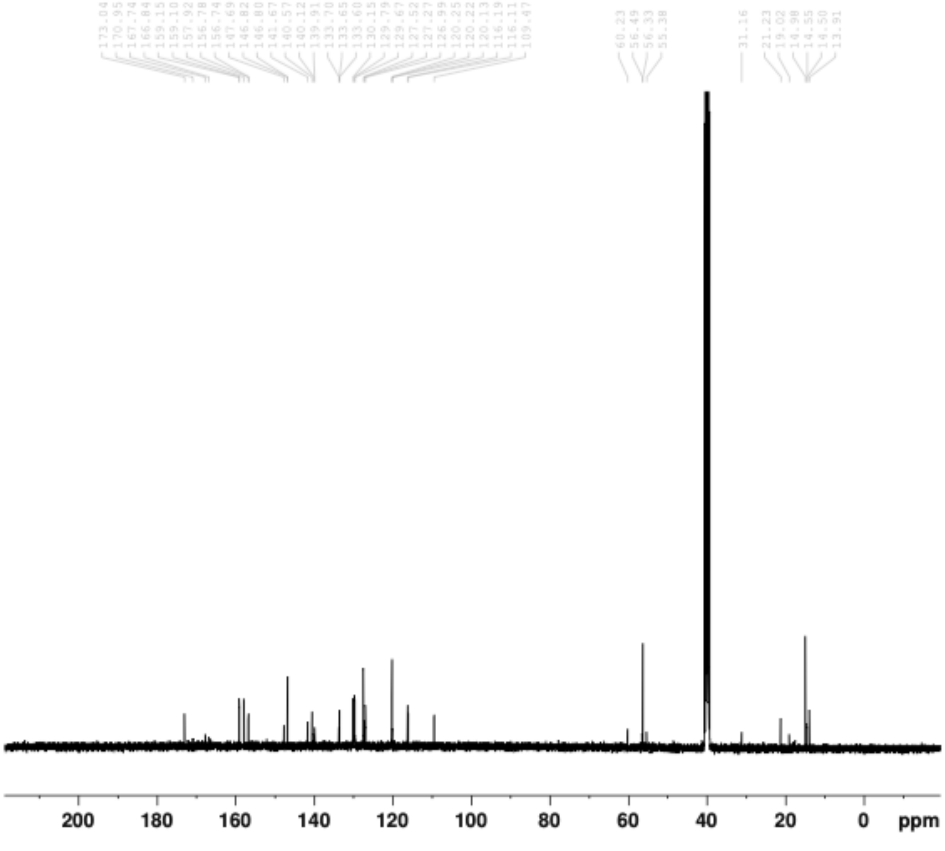

^{1}H NMR of compound **13l** (400MHz, DMSO-d_6)

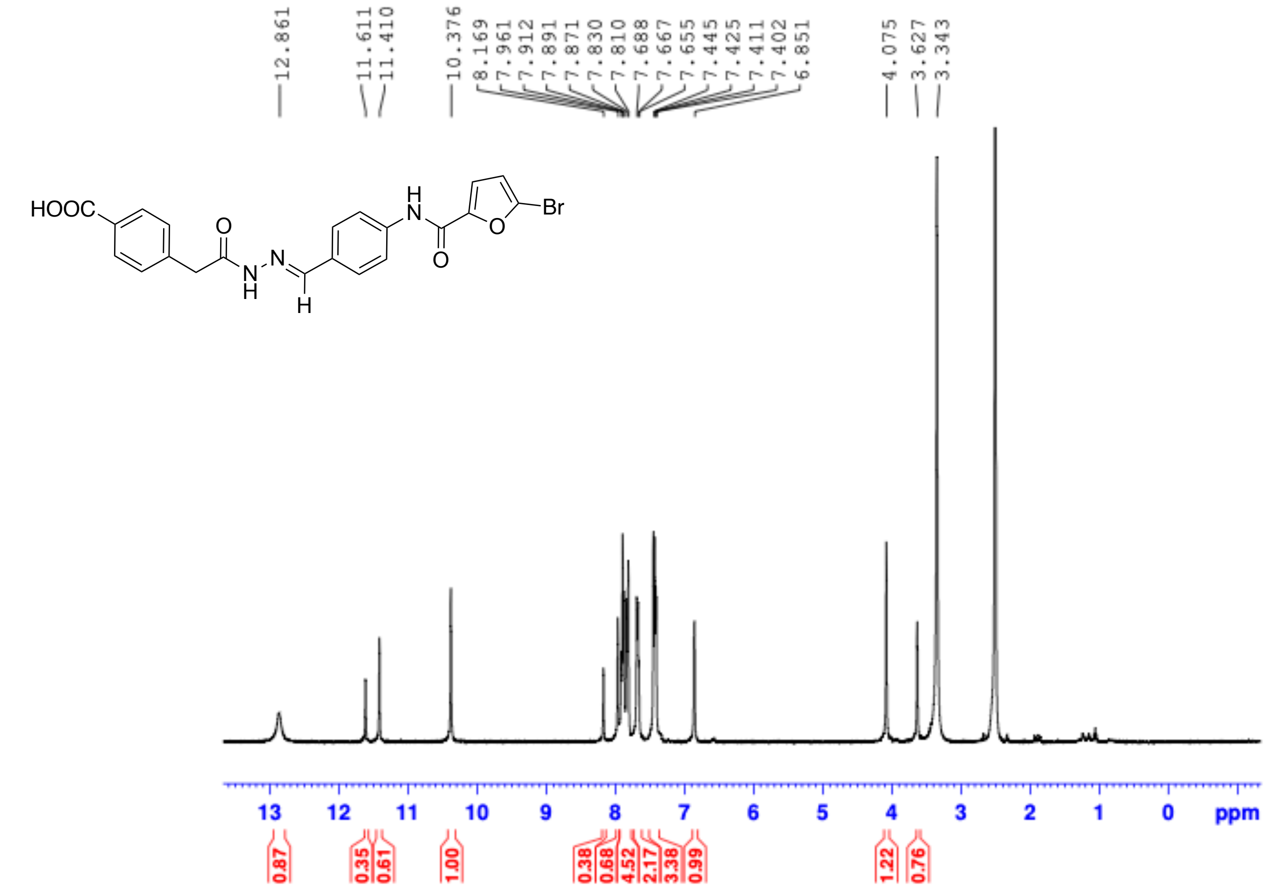

^{13}C NMR of compound **13l** (100MHz, DMSO-d_6)

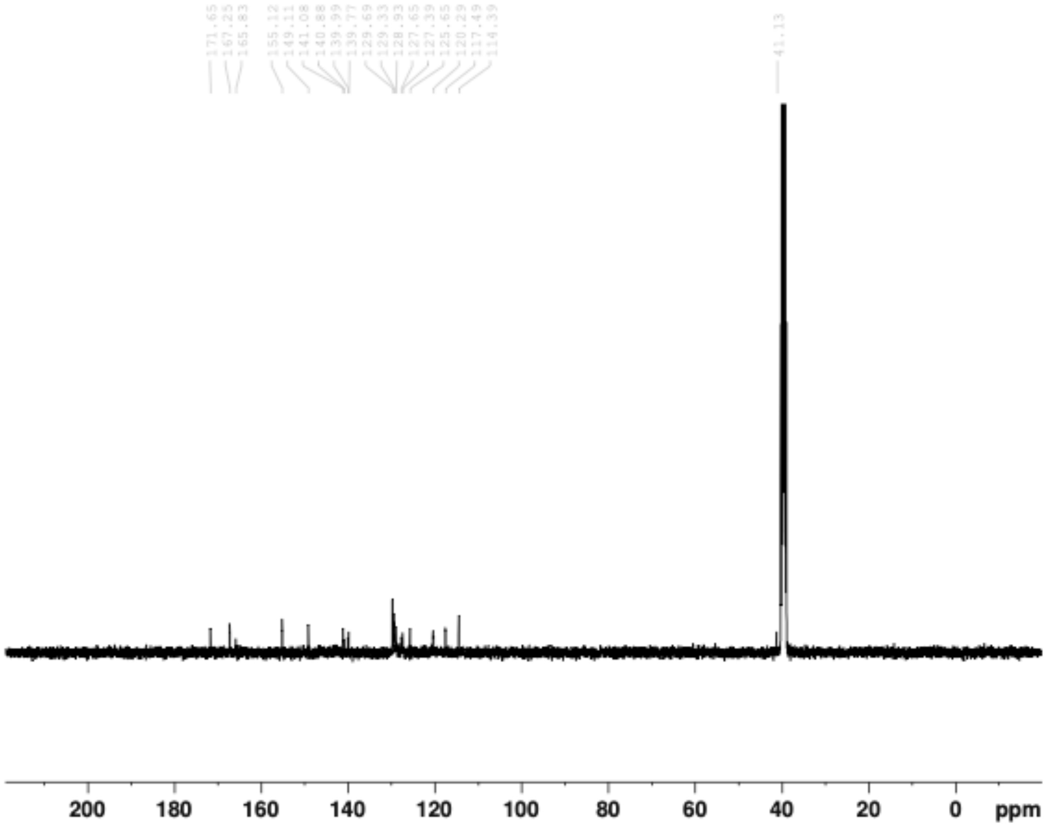

^{1}H NMR of compound **13m** (400MHz, DMSO-d_6)

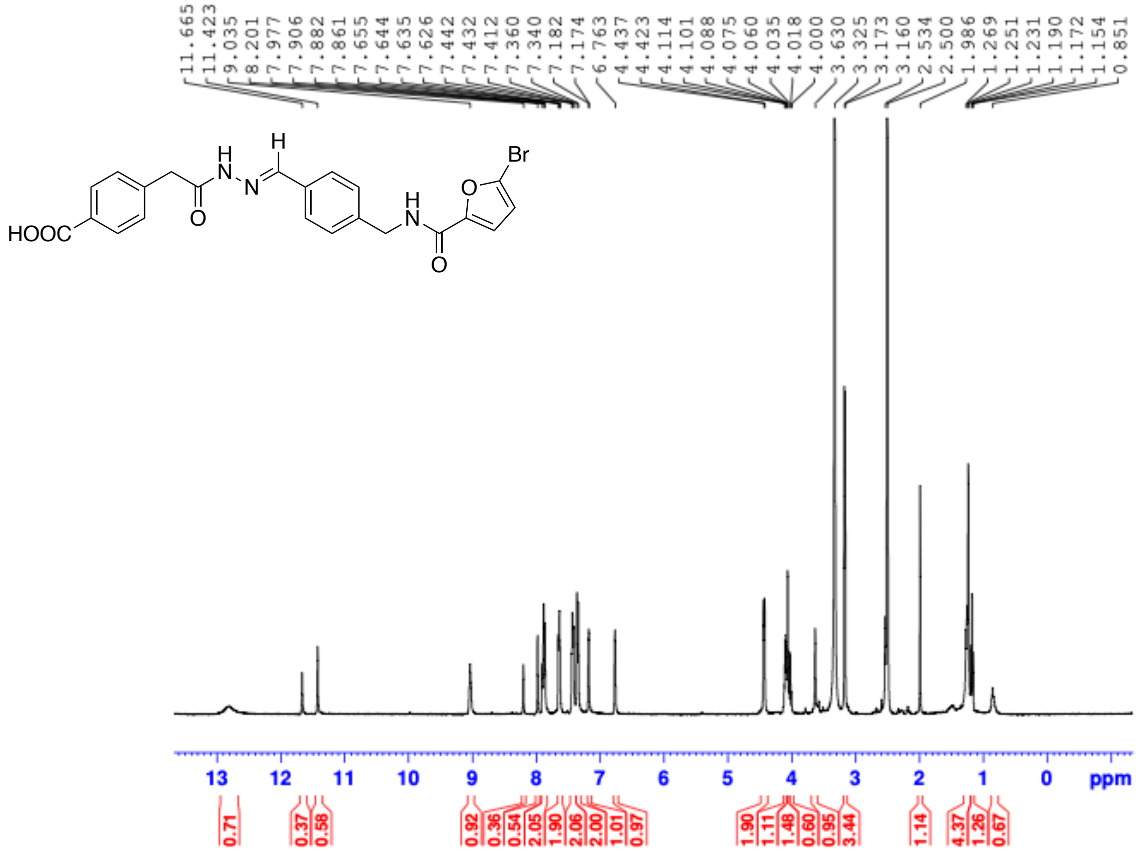

^{13} C NMR of compound **13m** (100MHz, DMSO-d_6)

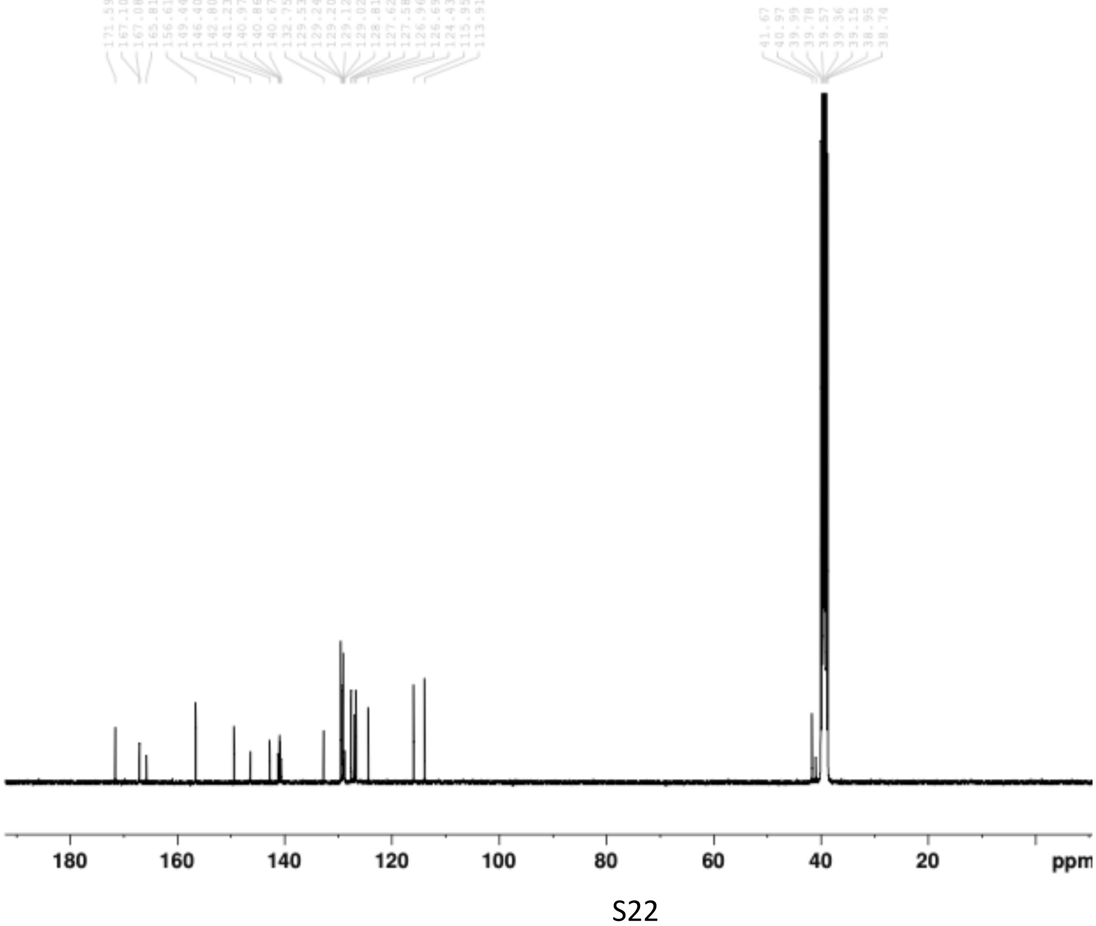

^{1}H NMR of compound **13n** (400MHz, DMSO-d_6)

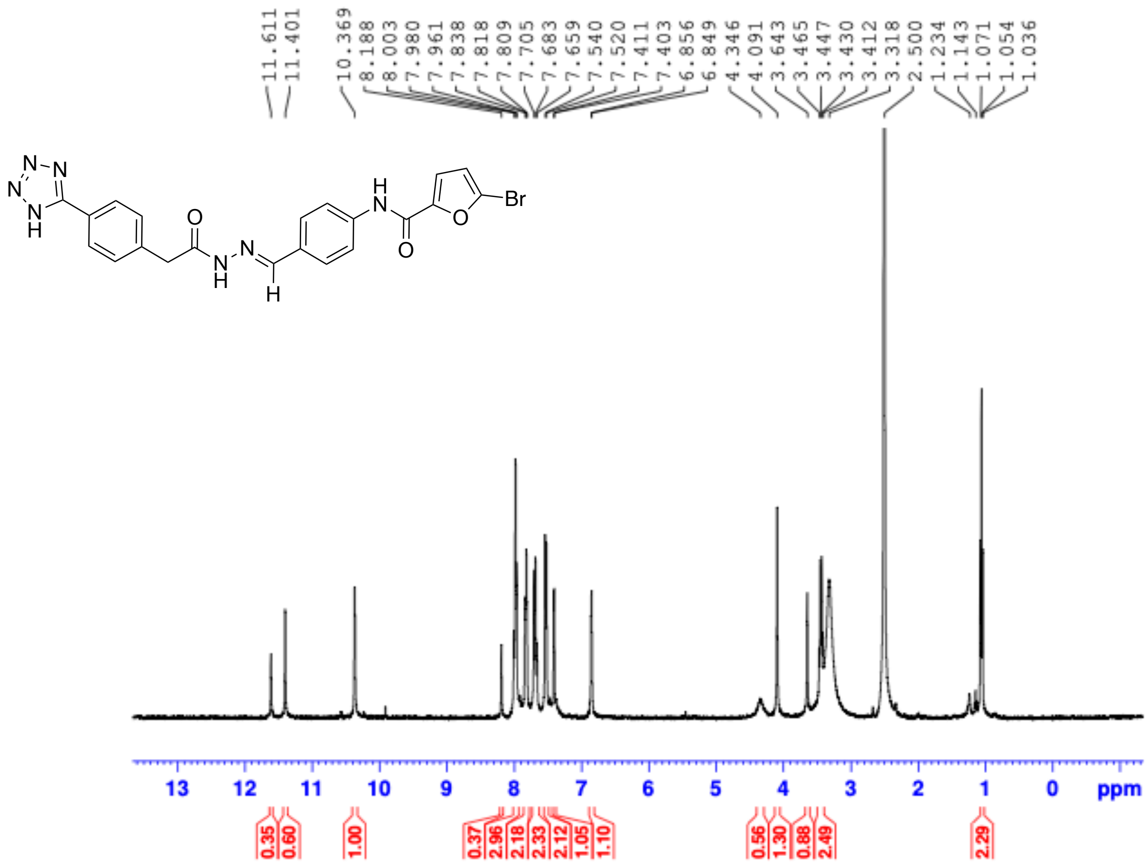

[13] C NMR of compound **13n** (100MHz, DMSO-d_6)

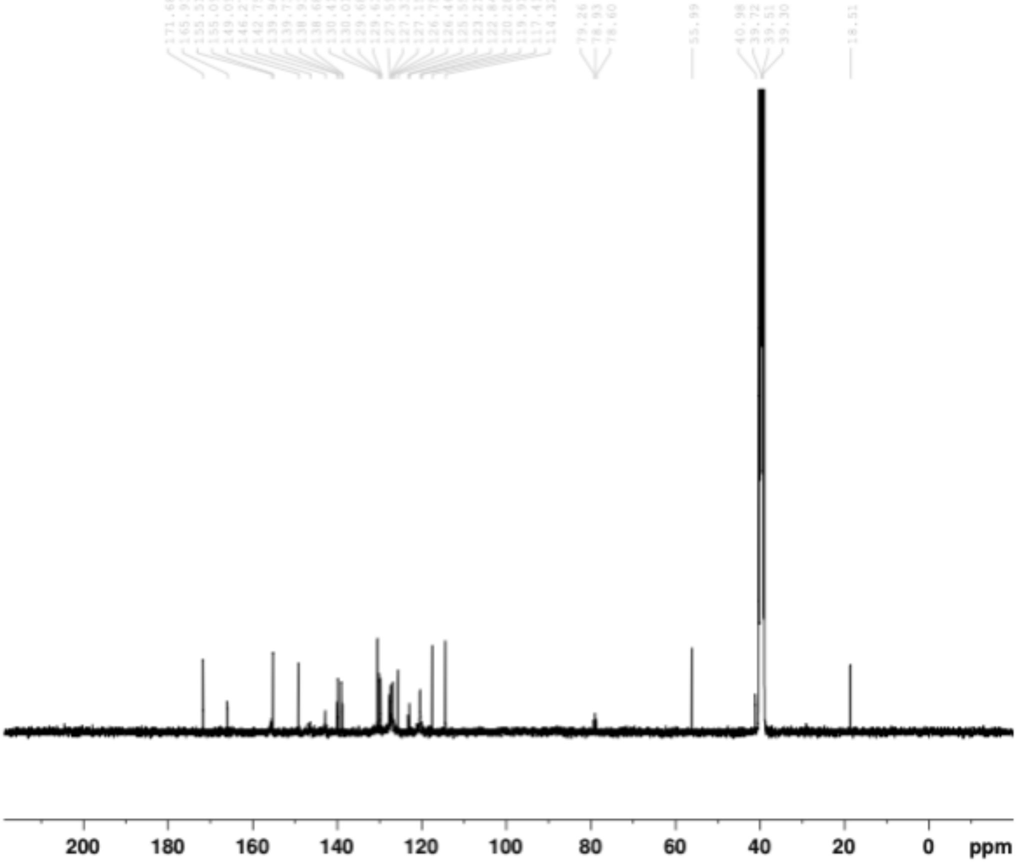

^{1}H NMR of compound **13o** (400MHz, DMSO-d_6)

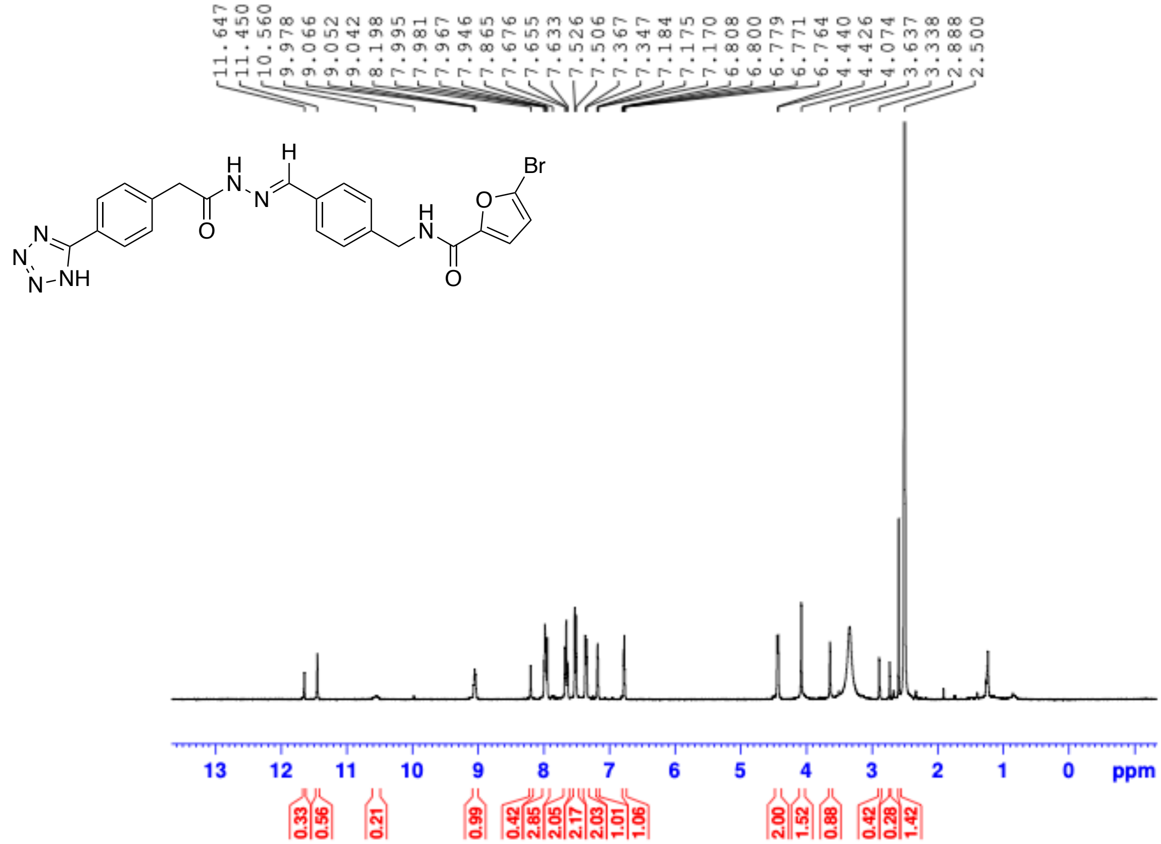

[13] C NMR of compound **13o** (100MHz, DMSO-d_6)

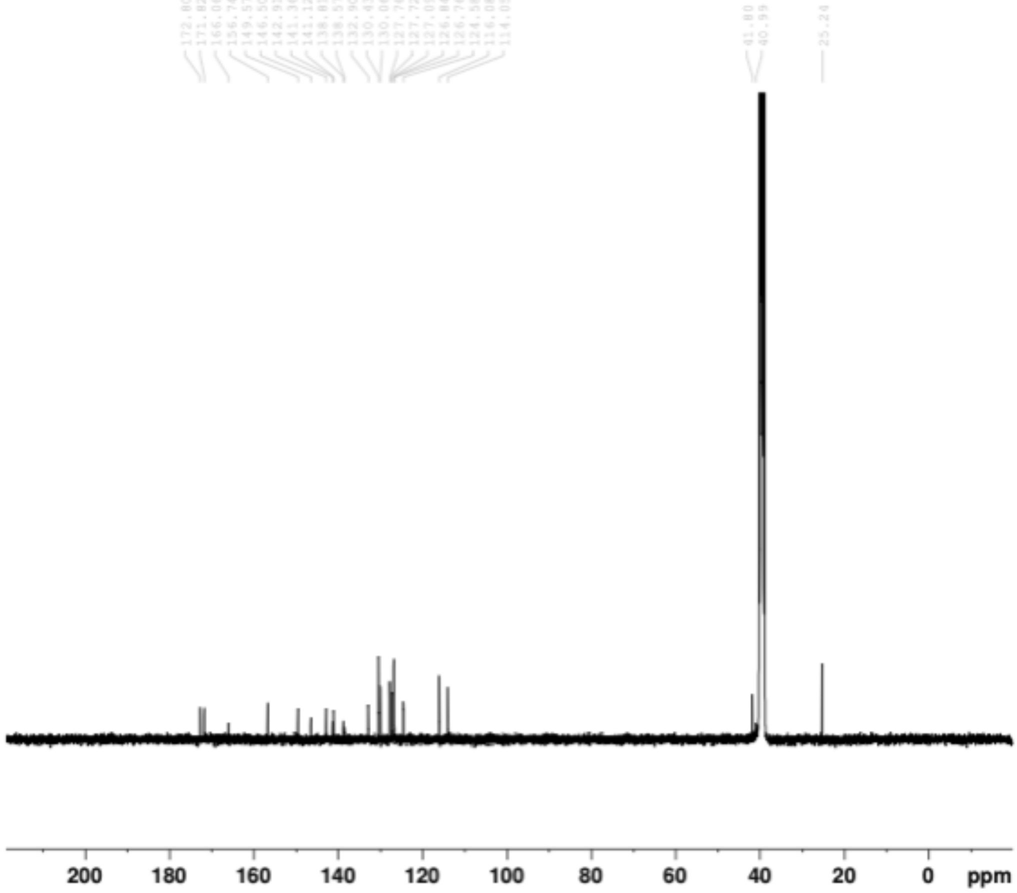

V. Representative HRMS spectra

HRSM spectrum of compound **13**

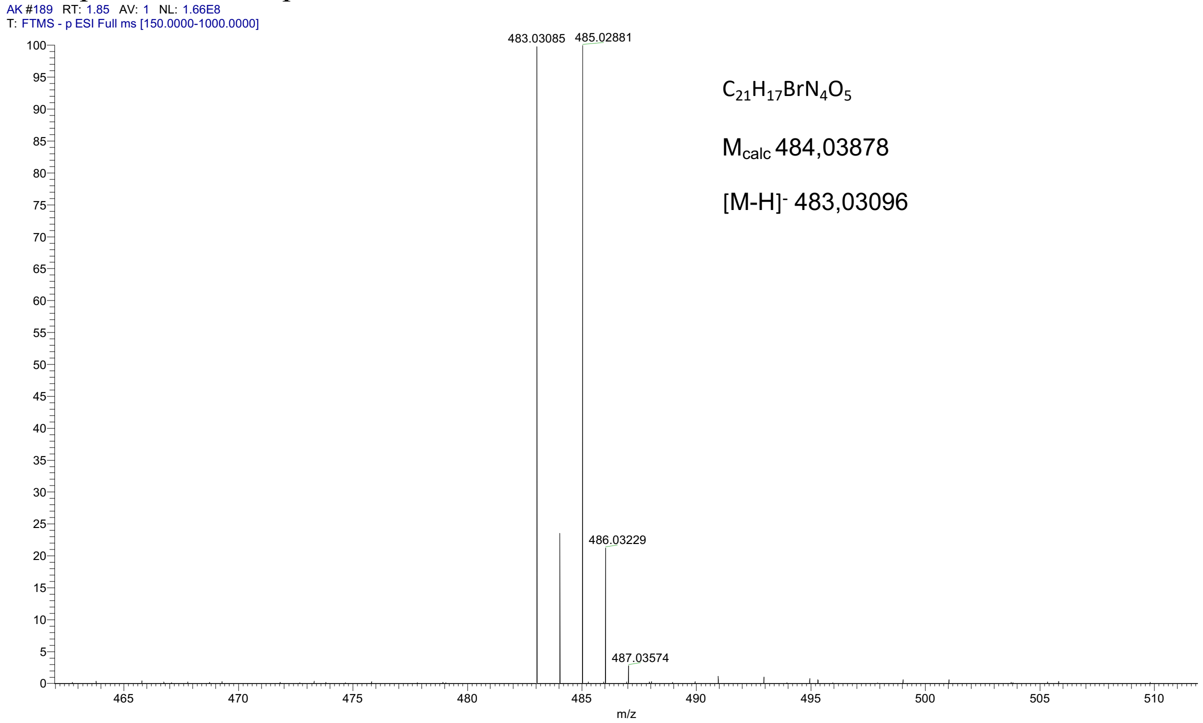

HRSM spectrum of compound **13a**

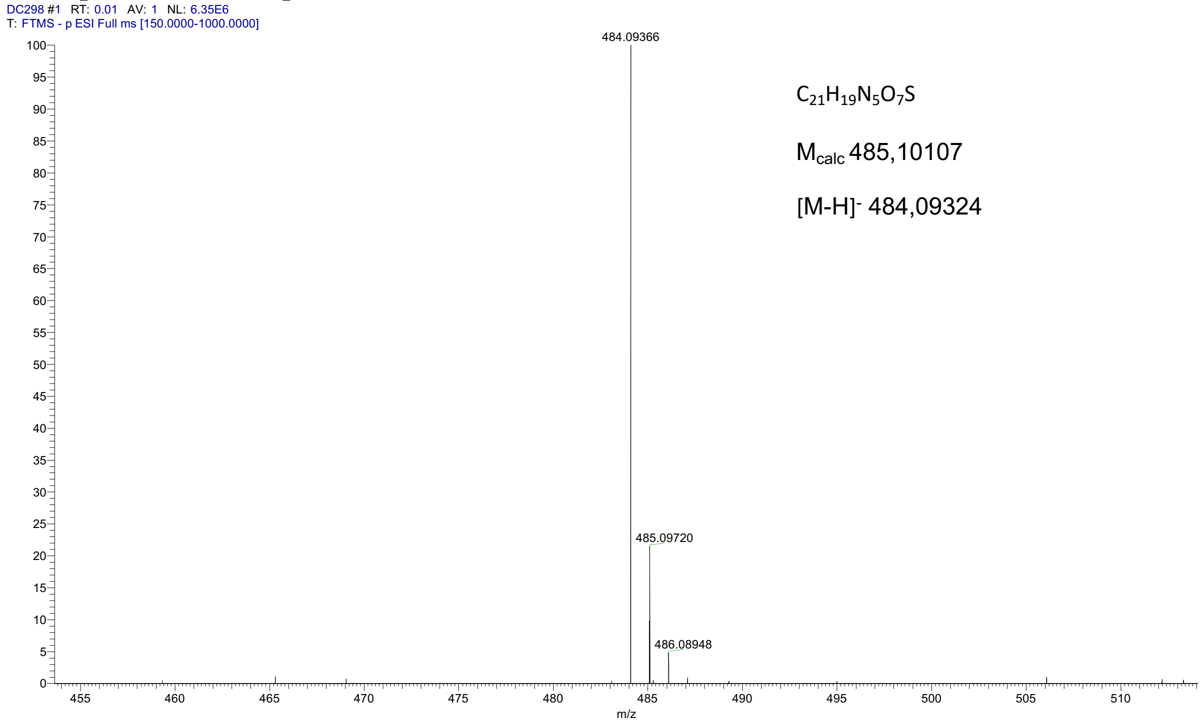

HRSM spectrum of compound **13b**

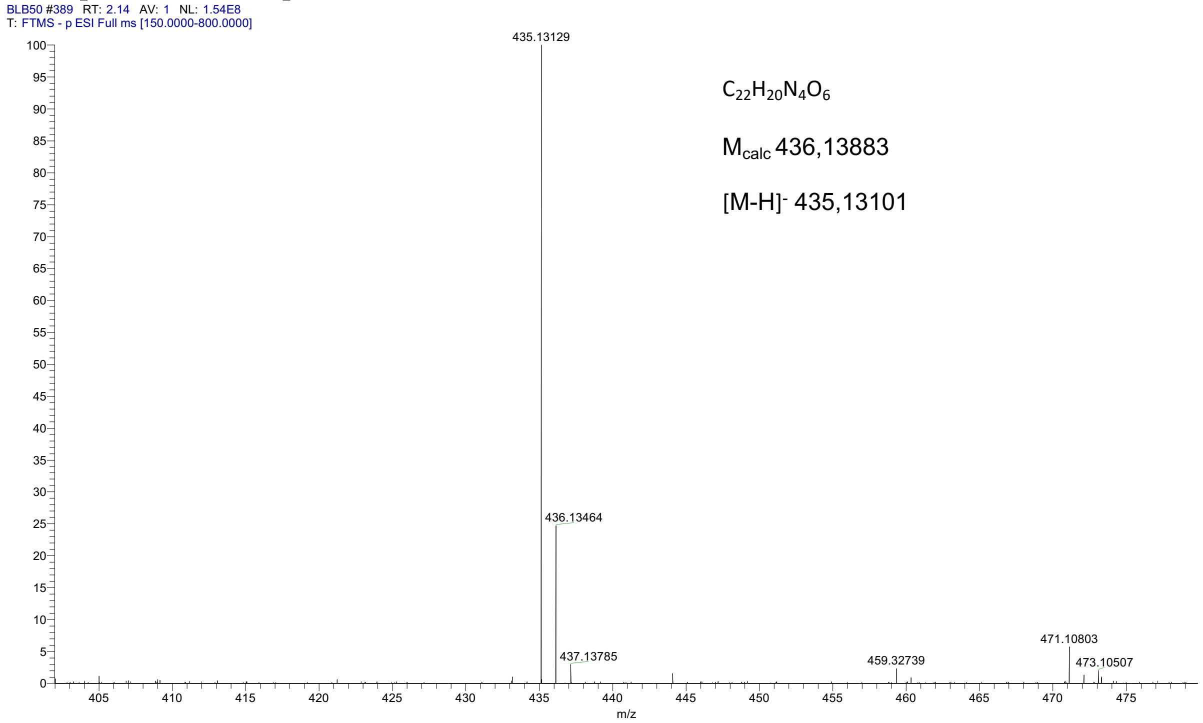

HRSM spectrum of compound **13c**

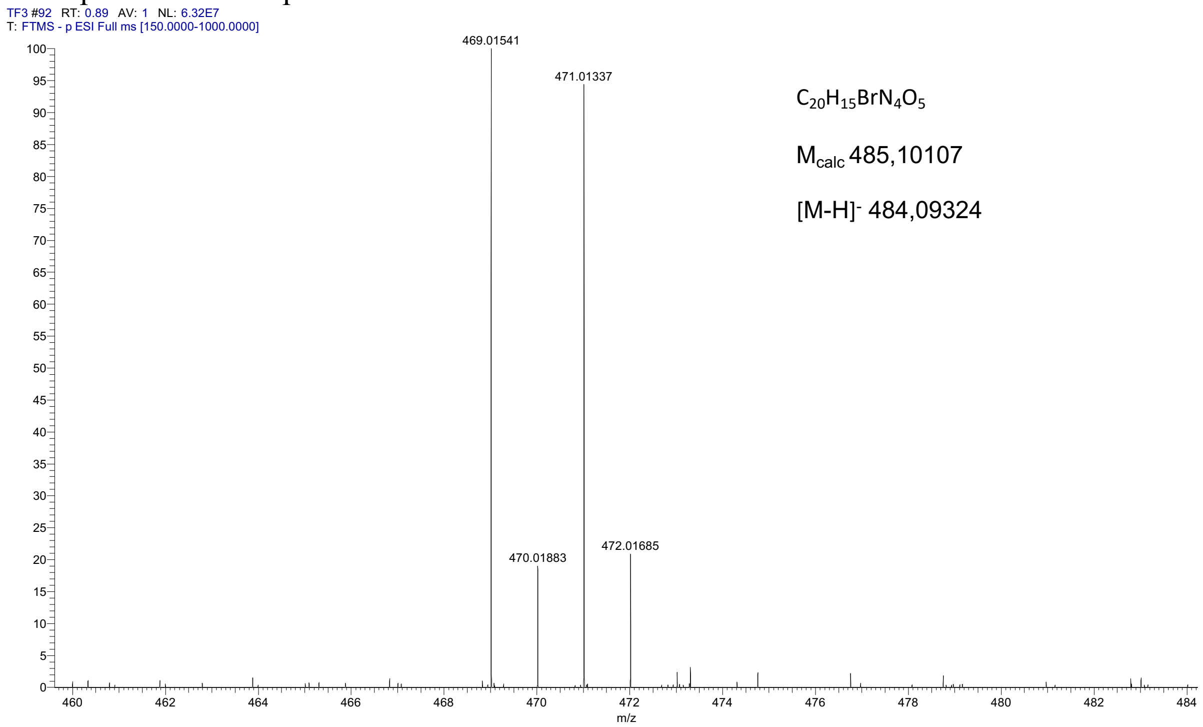

HRSM spectrum of compound **13d**

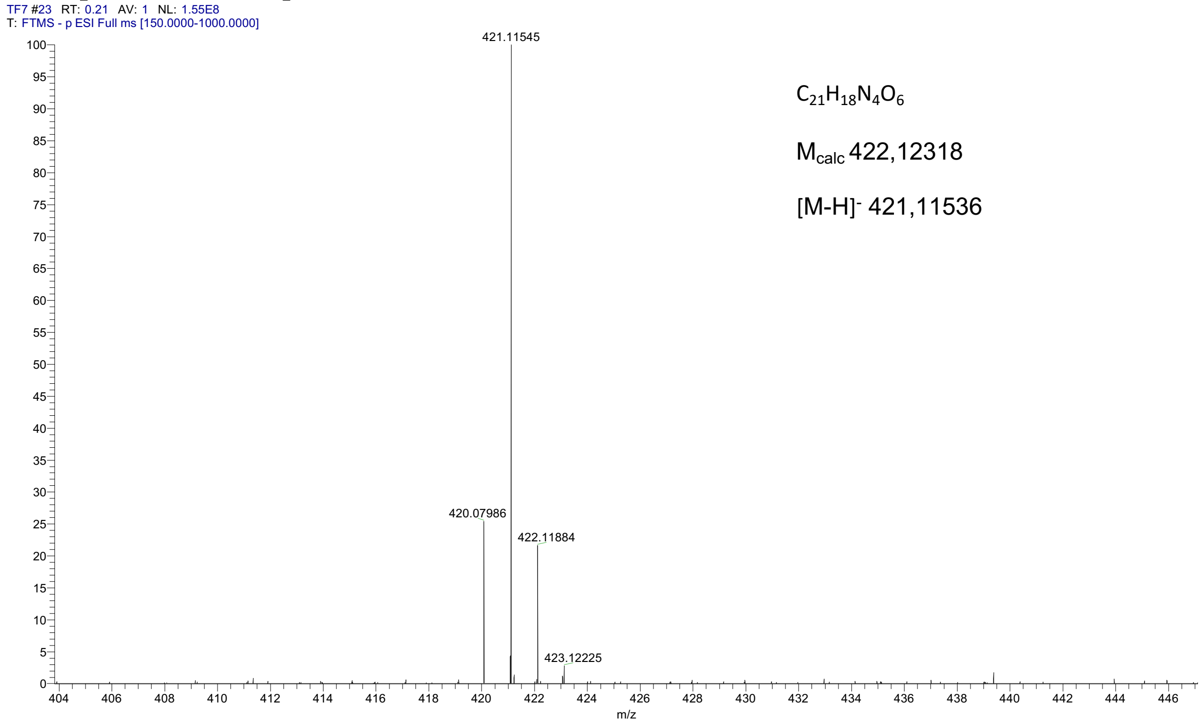

HRSM spectrum of compound **13e**

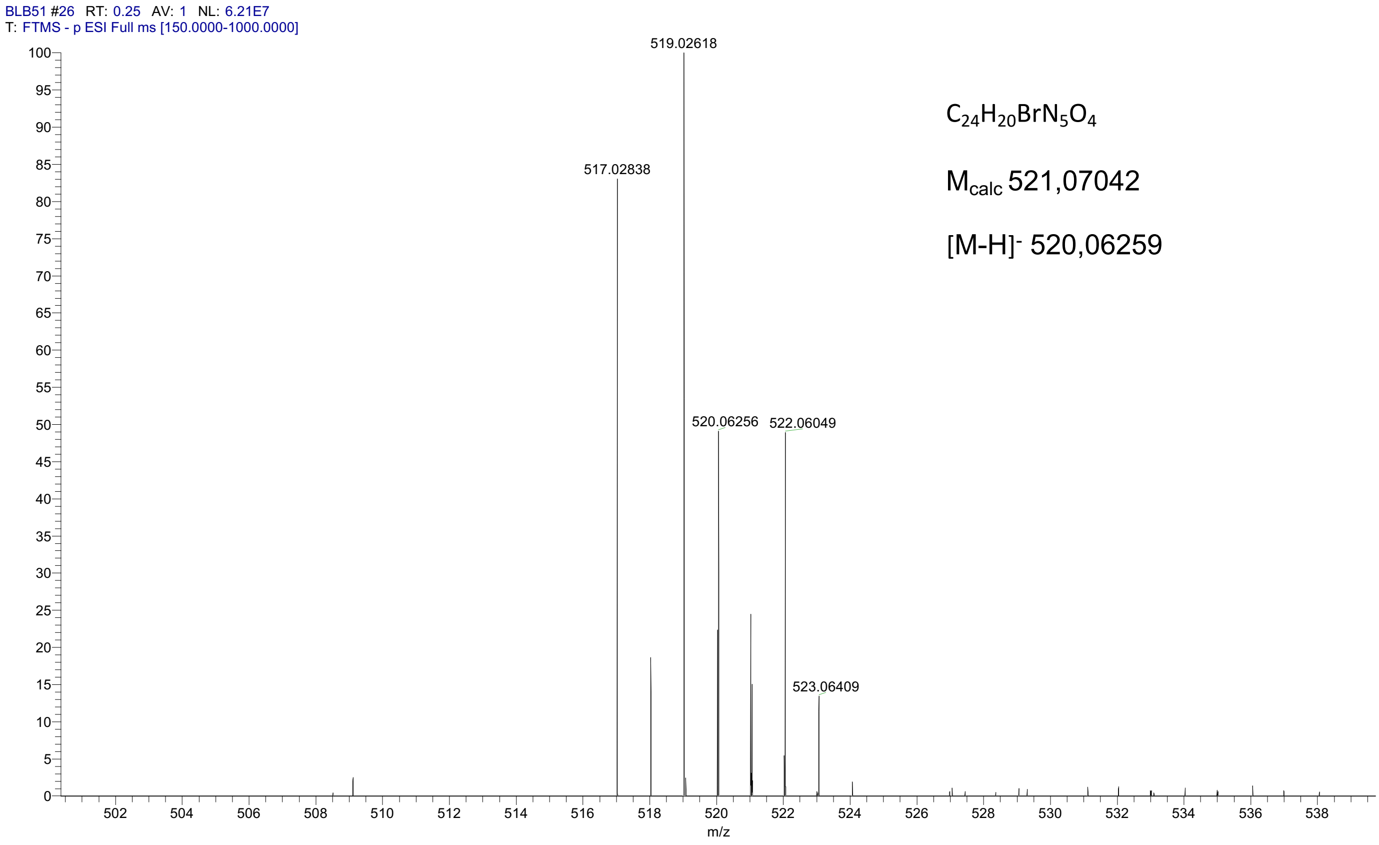

HRSM spectrum of compound **13f**

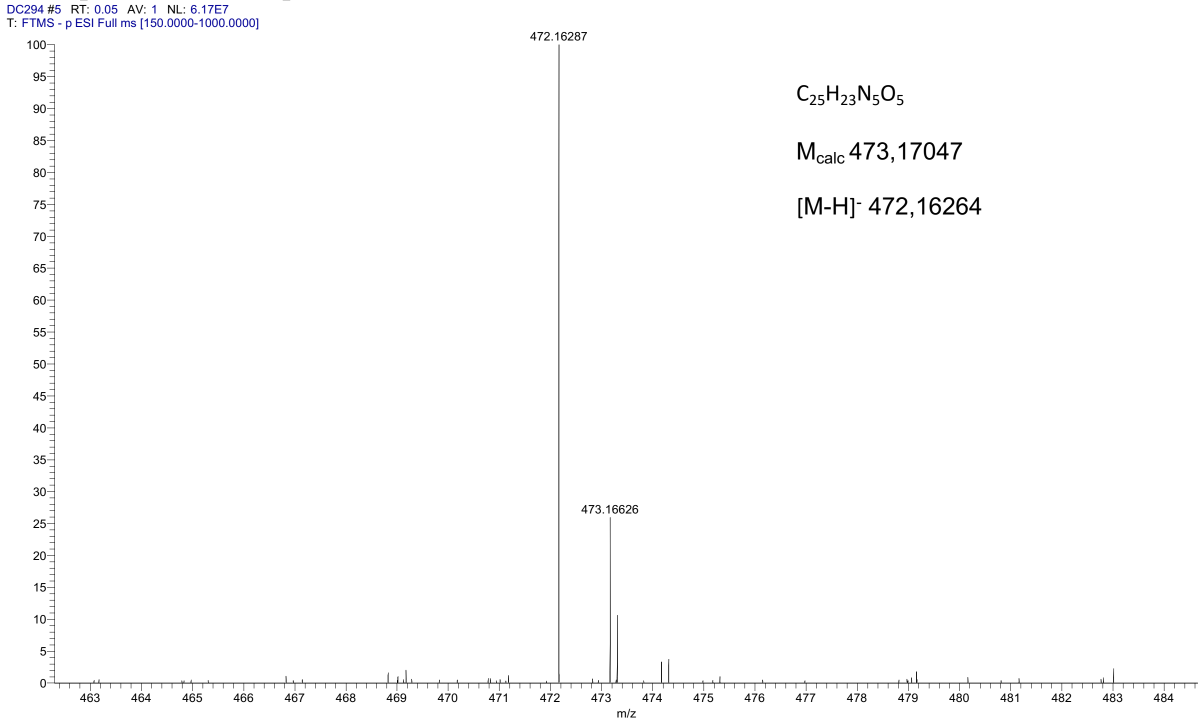

HRSM spectrum of compound **13g**

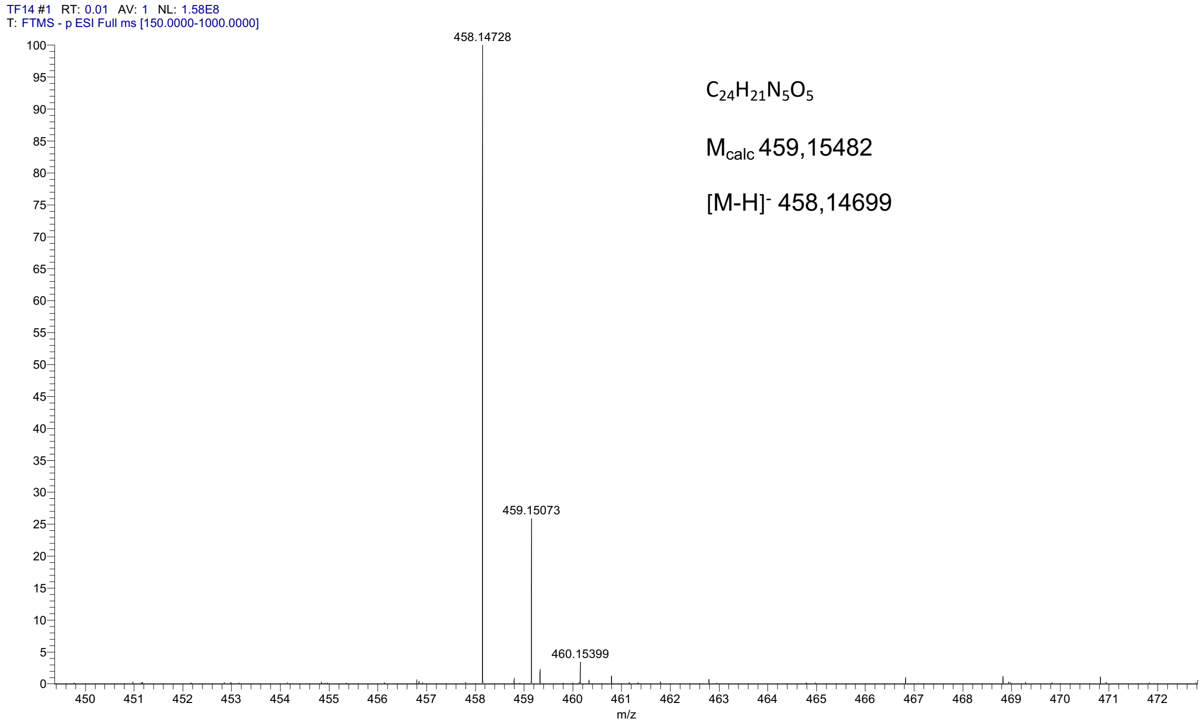

HRSM spectrum of compound **13h**

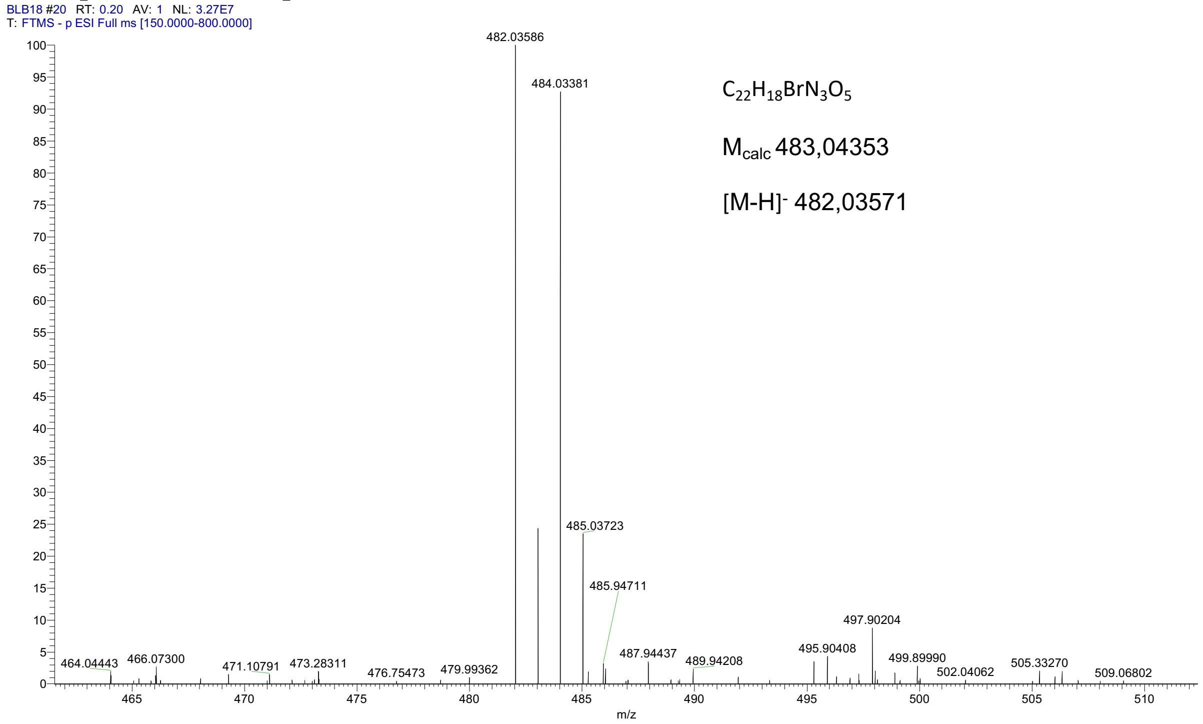

HRSM spectrum of compound **13i**

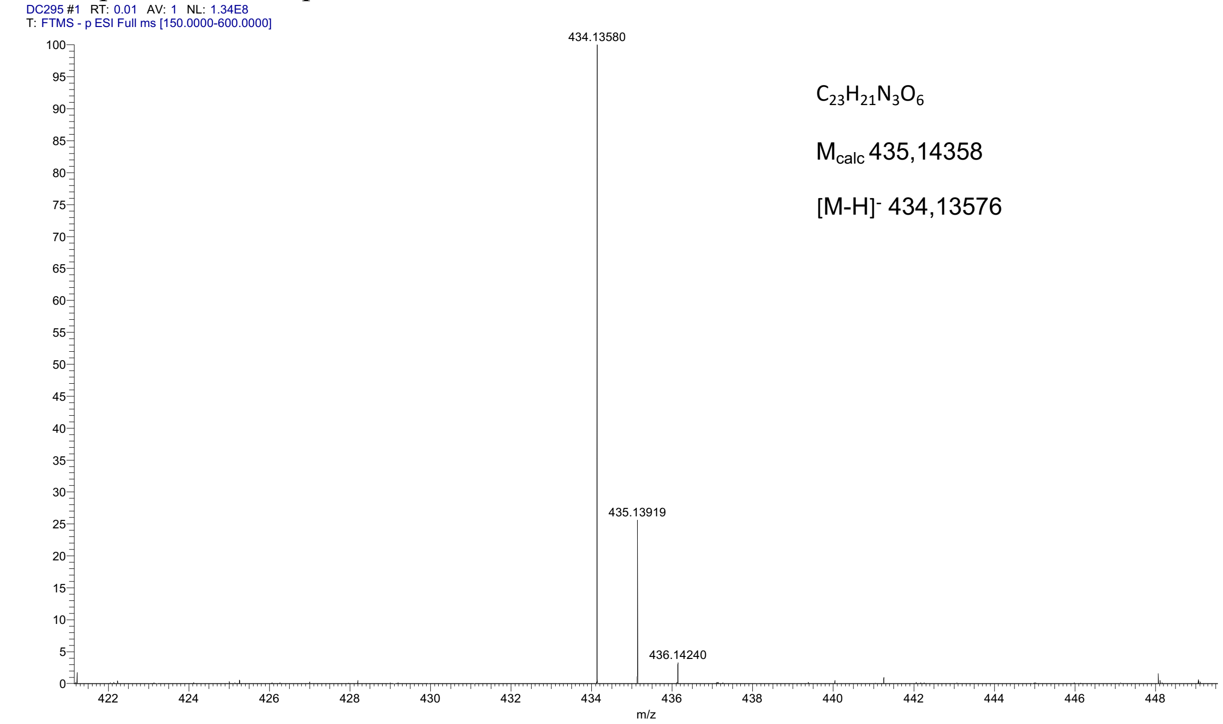

HRSM spectrum of compound **13l**

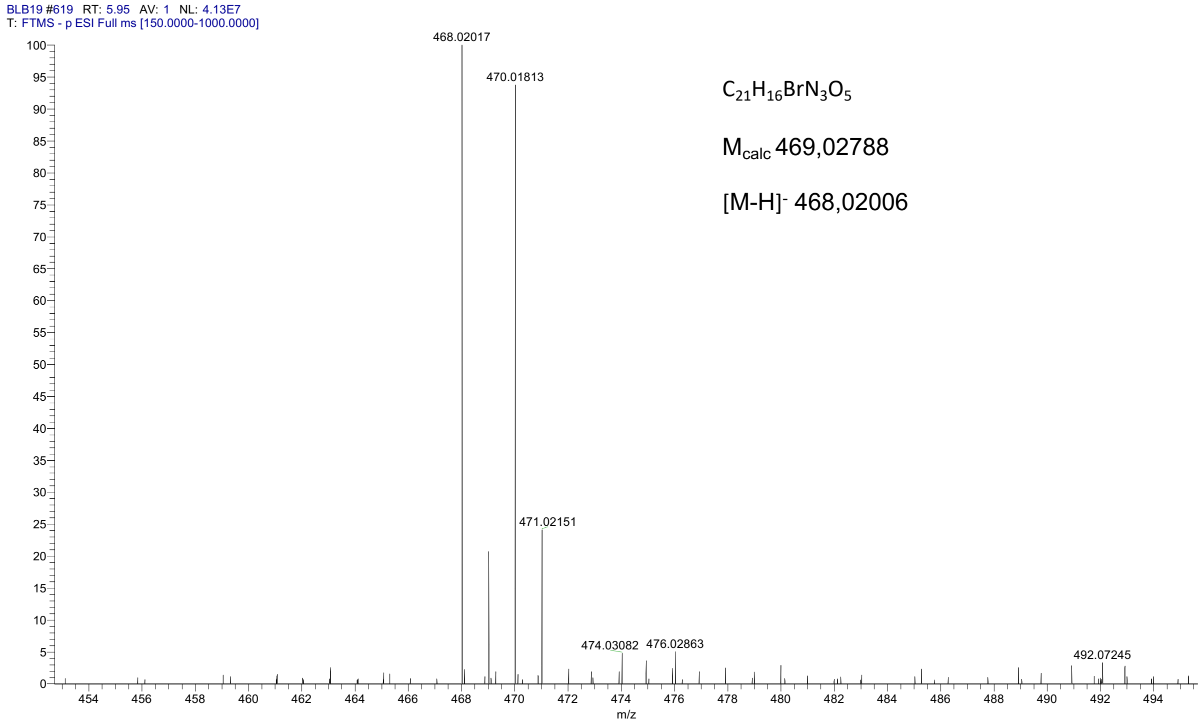

HRSM spectrum of compound **13m**

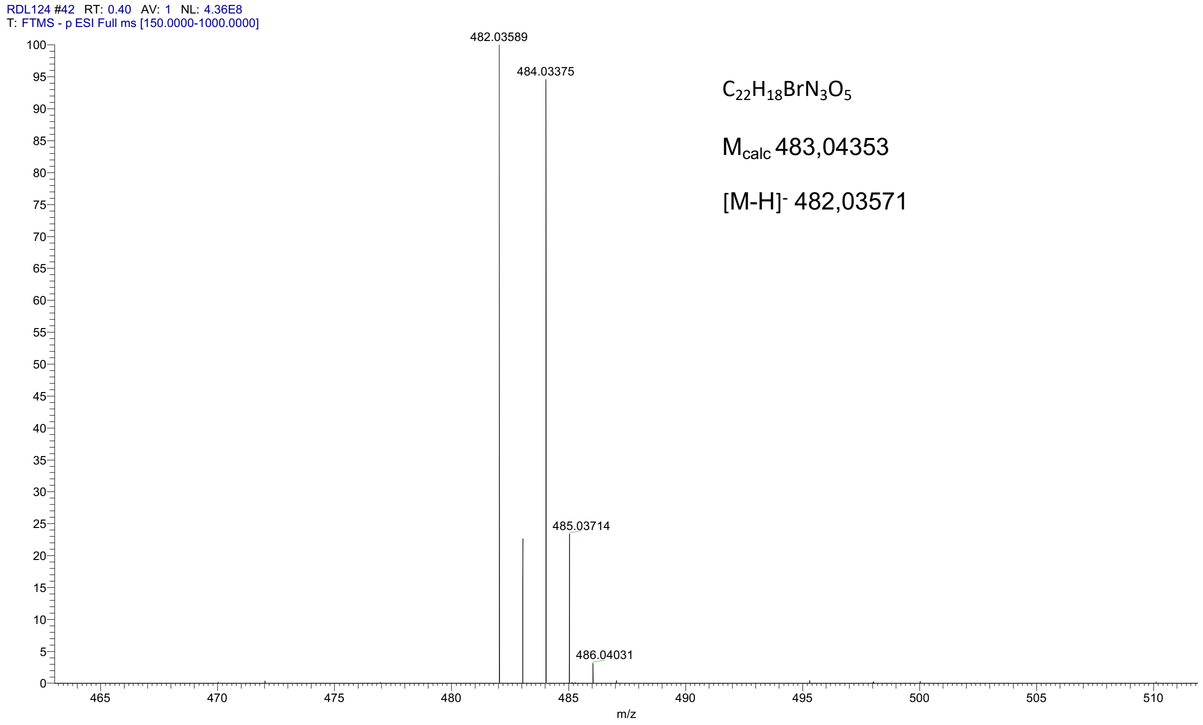

HRSM spectrum of compound **13n**

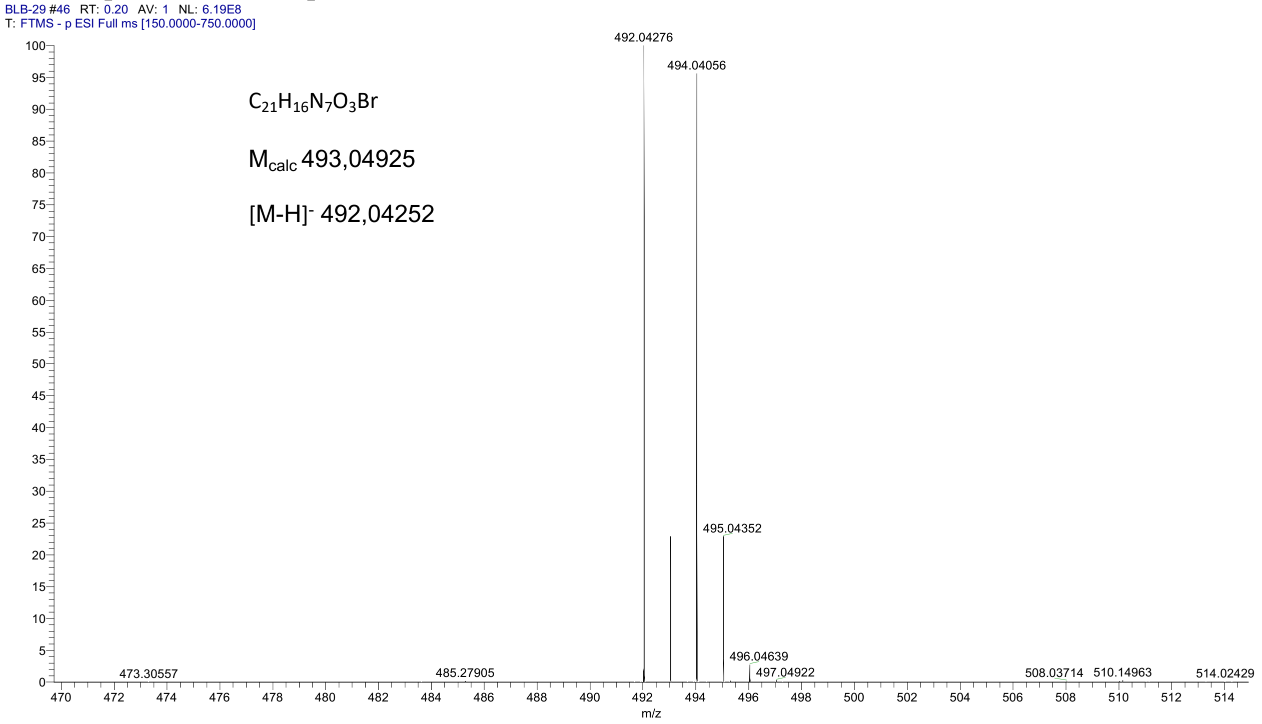

HRSM spectrum of compound **13o**

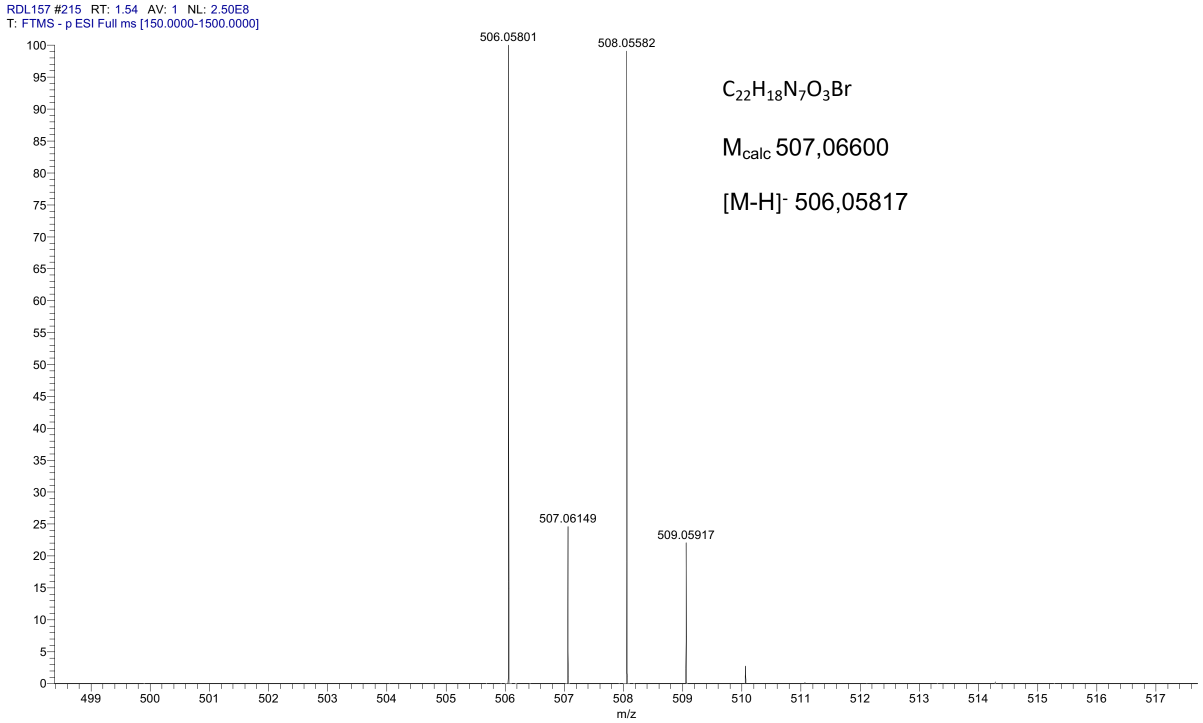

Supplement: Supplementary file 1 [file ijms-24-11098-s001.zip › ijms-2432125-supplementary.pdf]
